# MUFINS: multi-formalism interaction network simulator

**DOI:** 10.1038/npjsba.2016.32

**Published:** 2016-11-17

**Authors:** Huihai Wu, Axel von Kamp, Vytautas Leoncikas, Wataru Mori, Nilgun Sahin, Albert Gevorgyan, Catherine Linley, Marek Grabowski, Ahmad A Mannan, Nicholas Stoy, Graham R Stewart, Lara T Ward, David J M Lewis, Jacek Sroka, Hiroshi Matsuno, Steffen Klamt, Hans V Westerhoff, Johnjoe McFadden, Nicholas J Plant, Andrzej M Kierzek

**Affiliations:** 1 School of Biosciences and Medicine, Faculty of Health and Medical Sciences, University of Surrey, Guildford, UK; 2 Max Planck Institute for Dynamics of Complex Technical Systems, Magdeburg, Germany; 3 Graduate School of Science and Engineering and Faculty of Science, Yamaguchi University, Yoshida, Yamaguchi, Japan; 4 Molecular Cell Physiology, VU University Amsterdam, Amsterdam, The Netherlands; 5 MedImmune, Cambridge, UK; 6 Institute of Informatics, University of Warsaw, Warsaw, Poland; 7 Oncology DMPK, AstraZeneca, Alderley Park, Cheshire, UK; 8 Manchester Centre for Integrative Systems Biology, University of Manchester, Manchester, UK; 9 Synthetic Systems Biology, Netherlands Institute for Systems Biology, University of Amsterdam, Amsterdam, The Netherlands; 10 Simcyp Limited (a Certara Company), Sheffield, UK

## Abstract

Systems Biology has established numerous approaches for mechanistic modeling of molecular networks in the cell and a legacy of models. The current frontier is the integration of models expressed in different formalisms to address the multi-scale biological system organization challenge. We present MUFINS (MUlti-Formalism Interaction Network Simulator) software, implementing a unique set of approaches for multi-formalism simulation of interaction networks. We extend the constraint-based modeling (CBM) framework by incorporation of linear inhibition constraints, enabling for the first time linear modeling of networks simultaneously describing gene regulation, signaling and whole-cell metabolism at steady state. We present a use case where a logical hypergraph model of a regulatory network is expressed by linear constraints and integrated with a Genome-Scale Metabolic Network (GSMN) of mouse macrophage. We experimentally validate predictions, demonstrating application of our software in an iterative cycle of hypothesis generation, validation and model refinement. MUFINS incorporates an extended version of our Quasi-Steady State Petri Net approach to integrate dynamic models with CBM, which we demonstrate through a dynamic model of cortisol signaling integrated with the human Recon2 GSMN and a model of nutrient dynamics in physiological compartments. Finally, we implement a number of methods for deriving metabolic states from ~omics data, including our new variant of the iMAT congruency approach. We compare our approach with iMAT through the analysis of 262 individual tumor transcriptomes, recovering features of metabolic reprogramming in cancer. The software provides graphics user interface with network visualization, which facilitates use by researchers who are not experienced in coding and mathematical modeling environments.

## Introduction

During the last two decades, Systems Biology has established numerous approaches to represent molecular biology knowledge in the form of mechanistic molecular interaction network models. This is accompanied by a legacy of thousands of experimentally validated models. Stochastic kinetic simulations provide the most detailed quantitative description, where individual reactive collisions occurring at exact times in single cells are simulated by the Gillespie algorithm.^1^ The Ordinary Differential Equation (ODE) formalism applied to study the time evolution of average molecular concentration in cellular population is a workhorse of quantitative modeling.^2^ Although quantitative biology is developing rapidly, it is still not possible to parameterize quantitative dynamic models of whole-cell scale networks and simulate genotype–phenotype relationship. Rather, Constrained Based Modeling (CBM) has achieved spectacular success in modeling metabolism at the full genome scale.^3,4^ The metabolic network can be modeled at quasi-steady state owing to timescale separation from gene regulation. This enables exploration of metabolic flux distributions consistent with stoichiometric and thermodynamic constraints as well as flux measurements and constraints formulated according to ~omics data on enzymatic gene expression.^5^ Modeling of large-scale gene regulatory and signaling networks is much more challenging and a number of qualitative simulation approaches have been formulated, such as analysis of steady states in logical hypergraphs,^6^ enumeration of states in dynamic Boolean models,^7^ Monte Carlo exploration of the alternative molecular transition sequences constrained by network connectivity expressed in Petri Net formalism.^8,9^ Application of these methods has led to a legacy of models describing different levels of cellular organizations in different modeling frameworks. A large proportion of these models are already expressed in Systems Biology Markup Language (SBML)^10^ and over 1,000 literature-based models are available in the most recent version of BioModels.^11^ Notably, BioModels is attracting interest from the Physiologically Based Pharmacokinetic (PBPK) modeling field,^12^ where ODE models of substance concentrations in physiological compartments are routinely used to inform drug development in the pharmaceutical industry.

Given this state-of-the-art and the multi-scale nature of biological systems, the current challenge is integration of models expressed in different formalisms towards a multi-formalism simulation covering all scales of biological organization. The model of *Mycoplasma* is currently the most complete *in silico* cell,^13^ and demonstrates that mechanistic modeling of the genotype–phenotype relationship requires the integration of subsystem models describing different spatial and temporal scales constructed in different formalisms. The application of the multi-formalism approach toward modeling the relationship between genotype and human physiology is an emerging field and an important component of the personalized medicine challenge. For example, the integration of a PBPK model with human liver-specific GSMN has allowed robust prediction of therapeutic response in humans.^14^ In our recent work, we integrated a liver-specific GSMN with a qualitative model of a large-scale regulatory network,^9^ demonstrating how integration of gene regulation and metabolism in the context of physiological modeling can provide novel insights into toxicology, non-alcoholic liver disease and metabolic syndrome. This was achieved by the application of our novel Quasi-Steady State Petri Net (QSSPN)^9^ approach integrating CBM and qualitative, Monte Carlo simulation of a regulatory network represented as a Signaling Petri Net.^15^ Numerous alternate methods have been proposed to integrate GSMNs with ODE^16^ and logical^17^ dynamic models, as well as hybrid algorithms, bridging the gap between exact stochastic and ODE regimes in fully parameterized dynamic models.^18^ To fully realize the potential of computational modeling, it is now imperative to develop software packages that allow the development and simulation of multi-formalism models in a user interface that is approachable for experimental scientists.

Here, we present MUFINS (MUlti-Formalism Interaction Network Simulator) software and argue that it is the first general software with Graphics User Interface (GUI) capable of integrating models developed in all major modeling frameworks of Computational Systems Biology. This is demonstrated through Use Cases; first, a model of the mammalian macrophage where linear inhibitory constraints are for the first time used to integrate a logical model of cellular signaling with the GSMN for the mammalian cell; second, a model of the human hepatocyte, where an extended version of our QSSPN method is used to integrate a human GSMN, a detailed kinetic model of cortisol signaling and a PBPK model; third, the analysis of clinical transcriptome data in the context of human GSMN using a novel variant of ~omics data integration approach. The Use Cases involve laboratory experiments to demonstrate how experimental biologists can utilize the MUFINS GUI in the iterative cycle of model development, hypothesis generation, experimental validation and model refinement. Moreover, a comprehensive comparison with other software shows that MUFINS implements the largest number of CBM methods under a GUI with interactive network visualization. Thus, MUFINS is uniquely suited for the development and simulation of multi-formalism models by a wide user community including experimental scientists with no/limited experience with programmatic interfaces and mathematical modeling environments.

## Software overview

**Figure 1:**
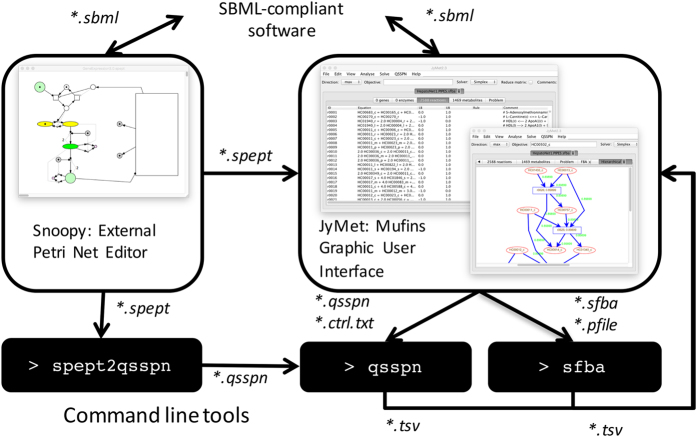
Overview of MUFINS. All the calculations are performed by two computational engines, which can be also run as stand-alone command line tools. The sfba implements CBM methods and qsspn performs QSSPN simulations. JyMet is a graphic interface to all methods providing spreadsheet representation of models and results as well as metabolic network visualization and plots. JyMet writes input files for computational engines, starts calculations, imports output files and displays results. In the case of QSSPN simulations, Petri Net connectivity can be graphically edited by Snoopy software, a standard Petri Net tool, which we use as external editor. JyMet imports Snoopy files and provides spreadsheet interface allowing editing of QSSPN parameters or independent creation of entire QSSPN model. Conversion of Snoopy files directly to qsspn engine is also possible with command line python script spept2qsspn. Both JyMet and Snoopy import and export SBML file providing connectivity to other SBML-compliant tools. The file formats used for software component communication are indicated by their default extensions and described in Supplementary File Formats. CBM, constraint-based modeling; MUFINS, MUlti-Formalism Interaction Network Simulator; SBML, Systems Biology Markup Language; QSSPN, Quasi-Steady State Petri Net.

Figure 1 provides an overview of the MUFINS architecture. All simulations are performed in *sfba* and *qsspn*, two computational engines written in C++, which are run either through a GUI or in command line mode. The *sfba* code originates from our SurreyFBA software^19^ and implements a comprehensive set of CBM approaches. The major new multi-formalism simulation feature added is linear inhibitor and activator constraints, which are described and validated in Use Case 1. In addition to the basic CBM methods available in SurreyFBA, the sfba engine of MUFINS implements a large number of ~omics data integration algorithms such as iMAT,^20^ GIMME,^20^ GIM3E,^21^ GNI^22^ and our DPA.^23^ The GNI and DPA features of sfba have been already used to study *Mycobacterium tuberculosis* metabolism.^23–25^ Furthermore, we include Fast iMAT a new variant of iMAT approach demonstrated in Use Case 3 below. *sfba* uses the GLPK library for Linear Programming (LP) and Mixed Linear Integer Programming (MILP) calculations. However, as MILP implementation in GLPK is inefficient, a version of *sfba* ready for use with Gurobi library is provided to facilitate the application of MILP-based protocols (e.g., iMAT) on GSMN models.

The qsspn is a computational engine for integration of dynamic and CBM models. It implements QSSPN approach,^9^ where a dynamic model constructed in Petri Net (PN) formalism^23^ is connected to a steady-state Flux Balance Analysis (FBA^4^) through PN places setting FBA bounds and requesting evaluation of objective functions. Previously, we integrated a qualitative PN model with a hepatocyte GSMN and explored its qualitative dynamic behaviors through Monte Carlo simulation.^15^ Here we present a new version of the engine, providing full support for continuous Petri Nets, implementing ODE models and stochastic Petri Nets representing exact stochastic simulations. Use Case 2 demonstrates the first integration of quantitative models of gene regulation with human Recon2 GSMN coupled to an ODE model of nutrients in physiological compartments. The qualitative simulation features are also further extended by implementation of *QSSPNclient*, a version of qsspn, which speeds up sampling of alternative qualitative trajectories by executing FBA on a server that tabulates repeated evaluations of GSMN objective functions for re-occurring sets of bounds. The new version of the QSSPN algorithm, *qsspn* solver and *QSSPNclient* are described in detail as Supplementary Information (page 32).

Connectivity of a Petri Net representing the dynamical part of the QSSPN model and its interactions with GSMN can be graphically defined using the Petri Net editor Snoopy:^25^ while Snoopy provides PN simulation, it does not implement QSSPN and thus is used here exclusively as an external editor of PN connectivity. Parameters specific to *qsspn* simulation can be provided either through the Comments section of place and transition objects, or added later through the MUFINS GUI. The Snoopy XML file is parsed by the *spept2qsspn* Python script, which generates the *qsspn* input file in *qsspn’s* native, human readable, text-file format. To facilitate future integration with other interfaces, a JSON-based input file format is also available.

JyMet is the GUI of MUFINS, and provides an interface for multi-formalism simulations to users who are not familiar with programming, mathematical modeling environments or working in command line. JyMet code is written in Jython (Java/Python), and originates from SurreyFBA.^19^ Here, it has been significantly extended through addition of QSSPN simulation and improved visualization of metabolic networks. JyMet integrates all elements of the MUFINS environment (Figure 1). It loads a Snoopy file defining PN connectivity and provides a table interface for definition of QSSPN specific parameters: place and transition types, lookup tables linking dynamic and GSMN variables, and arithmetic formulas describing complex rate laws. We note that users can build dynamic model within the QSSPN spreadsheet interface of JyMet without using Snoopy, but this is unlikely to be a preferable solution, due to the advantages of PN graphical modeling. Both sfba and qsspn can be run from JyMet and results are loaded to spreadsheets and plotting functions. Each table in JyMet can be exported in tab-separated format for further analysis with other software. Full features of JyMet are described in detail in software documentation and tutorials (MUFINS1.0_Doc/). Use Case 1 shows application of network visualization for exploration of alternative model solutions during the iterative cycle of network reconstruction, simulation, experimental validation and refinement.

The *sfba*, *qsspn* and *spept2qsspn* tools can be run as stand-alone command line tools without external dependencies. Thus MUFINS is ideal for integration with web and desktop interfaces as well as computational pipelines. Use Case 3 shows integration of *sfba* engine with computational pipeline for analysis of clinical transcriptome data. Previous version of *sfba* engine^19^ has been already used in web interfaces supporting development and publication of bacterial pathogen GSMN models^26,27^ and as one of computational engines in METEXPLORE web environment.^28^

MUFINS is an open source software, distributed under the GNU GPL license. It can be run on OS X, Windows and Linux sand the majority of calculations can be run without dependencies. The methods applying MILP to genome-scale models are likely to be very computationally expensive unless the Gurobi library is installed.

## Use Cases

Table 1 summarizes Use Cases illustrating the multi-formalism simulation abilities within MUFINS, including previously published works.

### Use Case 1: whole-cell metabolic reprogramming by signaling and gene regulatory networks in the mammalian macrophage

An important innovation in MUFINS is the ability to include stimulation and inhibition reactions within the genome-scale metabolic network. To demonstrate the utility of this approach to derive biological insights we present a use case entailing integration of gene and signaling regulatory networks with genome-scale metabolism for the mammalian macrophage.

**Figure 2:**
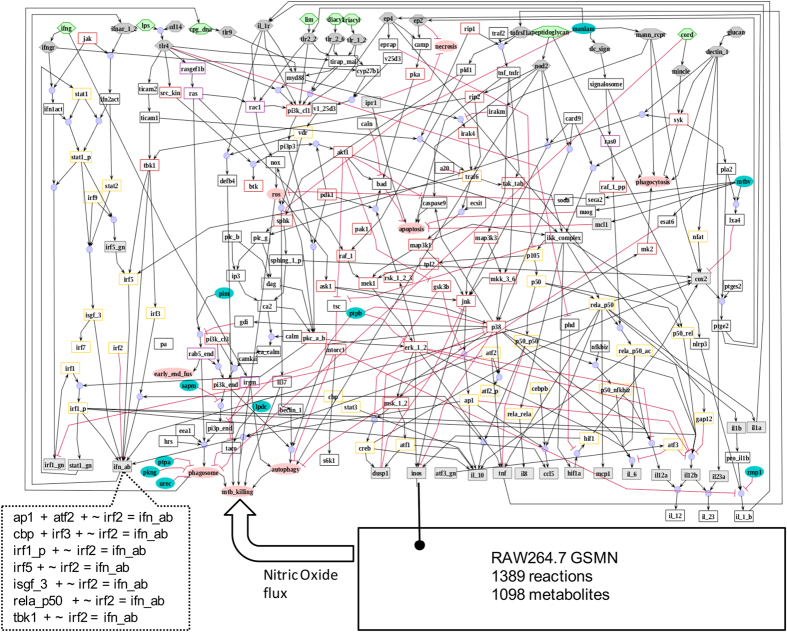
The model of cell signaling, gene regulation and whole-cell metabolism in RAW264.7 macrophage. A signaling and gene regulatory network of 286 interactions between 205 species, created in logical hypergraph formalism is shown. This network was subsequently converted to FBA formalism with linear inhibitory constraints and coupled to the RAW264.7 GSMN through regulation of the *iNOS* gene. Nitric oxide synthesis, a major metabolic flux in RAW264.7 macrophages responding to a pathogen, was then simulated using constraints derived from both stoichiometry of whole-cell metabolism and logical rules within a large-scale regulatory network. The inset shows the conversion of logical hyperedges determining the fate of ifn_ab to reaction formulas with linear inhibitor constraint: For all reactions producing ifn_ab, the molecule irf2 is added, preceded by the ‘~’ sign to indicate an inhibitor. This is parsed by MUFINS to mean that the reaction flux is inhibited (i.e., 0) if ifr2 is present. FBA, Flux Balance Analysis; GSMN, Genome-Scale Metabolic Network; *iNOS*, inducible nitric oxide synthase; MUFINS, MUlti-Formalism Interaction Network Simulator.

We applied MUFINS to integrate a logical hypergraph^6^ model of the large-scale regulatory network responsible for the pathogen response of mammalian macrophage with the published GSMN of the mouse RAW264.7 macrophage cell line.^29^ A signal transduction network of 286 interactions and 205 species was reconstructed in *CellNetAnalyzer* using logical hypergraph formalism:^6^ A manually created graph image is shown in Figure 2, with full description of model construction described in Supplementary Information, and a detailed description of species, logical formulas and literature references in Supplementary Table 1. Briefly, species within the regulatory network represent protein kinases, transcription factors, genes, antigens, cytokines and cellular behaviors (e.g., apoptosis) involved in the response of macrophages to bacterial pathogens.

To integrate signaling and metabolic networks, we have translated the logical hypergraph to a stoichiometric model with inhibitory constraints. Representation of inhibition in the CBM framework has always been a challenge, with proposed solutions generally being computationally expensive methods based on MILP.^30^ We have extended the approach of Vardi and colleagues^31^ and represented inhibition by linear constraints, enforcing a reciprocal relation between inhibitor production and inhibited flux. Differences between the original formulation and our extended implementation are presented as Supplementary Information. An example of the logical hypergraph conversion to MUFINS reaction formulas is shown in Figure 2. At the software level this is achieved by exporting CellNetAnalyzer logical rules as a text format and then using the text-replace function in Excel to change the formula format and create a MUFINS reaction table that can be opened by the JyMet GUI. We note these steps do not require programming experience. Subsequently, the model was edited in JyMet to define input fluxes. Flux Variability Analysis was undertaken to identify spurious activations in the model with all input fluxes constrained to 0. The details of these steps are given in Supplementary Information (page 7), where we also compare our model pre-processing steps with the much more complex, MILP-based protocol used by Vardi *et al*.^31^

**Figure 3:**
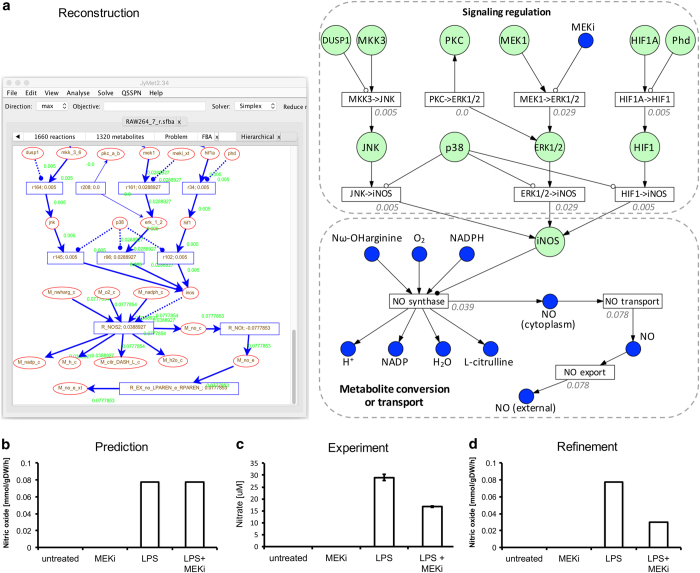
Mechanistic interpretation of experimental data on perturbation of whole-cell metabolic function by signaling network input and inhibitor. MUFINS was used to integrate a genome-scale metabolic model of the mouse macrophage (RAW264.7) with a large-scale regulatory network. Perturbation of whole-cell metabolism was simulated through activation and inhibition of the signaling network with external production of nitric oxide set as the objective function. Predicted data was then compared with the experimental data. (**a**) The left panel shows a screenshot of the JyMet interface, demonstrating on screen visualization of the reconstruction, created by automatic hierarchical layout with manual adjustment. Hatched lines are used to indicate regulatory signals, representing inhibition (circle end) or stimulation (arrow head). The right panel is a manually created image representing the pathway examined through JyMet; arrows represent signal flux, while open and filled circles represent inhibition and stimulation, respectively. The visualization depicts where signaling pathways converge on the *iNOS* gene, which is required for nitric oxide (NO) production in the whole-cell stoichiometric model. Flux rates for an example FBA solution are displayed on the network diagram; on the right panel only flux rates for each transitions are presented for clarity, while the left panel also shows the contribution of each substance to the flux. (**b**) The original reconstruction was able to predict the increase in NO production following stimulation with LPS, but not the impact of a MEK inhibitor, when compared with the experimental data of nitrate levels in RAW264.7 cell-conditioned medium (**c**). Nitrate can only be produced by non-enzymatic conversion of NO produced by RAW264.7 cells, and as there is no nitrate consumption in the medium, nitrate concentrations are proportional to nitric oxide production flux. (**d**) Refinement of the signaling network led to agreement between *in silico* prediction and *in vitro* measurement. The refinement was based upon two mechanistic hypotheses: (i) ERK1/2 is a more potent transcriptional activator of the *iNOS* gene than JNK and HIF1, and (ii) MEK1 is a more potent ERK1/2 kinase than PKC. FBA, Flux Balance Analysis; *iNOS*, inducible nitric oxide synthase; LPS, lipopolysaccharide; MUFINS, MUlti-Formalism Interaction Network Simulator; QSSPN, Quasi-Steady State Petri Net.

A unique feature of MUFINS is reconstruction of models that combine GSMNs and regulatory networks with linear inhibitory constraints. To demonstrate this capability we integrated the signal transduction network model described above with the published GSMN of the RAW264.7 macrophage cell line.^29^ We have focused on nitric oxide production, a major metabolic function of macrophages interacting with bacterial pathogens. Our regulatory network model describes the regulation of the inducible nitric oxide synthase (*iNOS*) gene, which we have added as an activator for the NO synthase reaction in the GSMN (reaction id: R_NOS2). We used a linear activator constraint, as described in Supplementary Information (page 31), to ensure that stoichiometry of the R_NOS2 reaction is not affected. Figure 3a shows a portion of the total regulatory network, specifically the immediate signaling pathways regulating *iNOS* gene expression. We used this integrated model to simulate nitric oxide production in response to lipopolysaccharide (LPS). Following Bordbar and colleagues,^29^ we constrained biomass reaction flux to 0.0281/h, which reproduces experimentally measured growth rates. We calculated the maximal extracellular nitric oxide production when LPS input flux to the regulatory network was opened or closed and when phosphorylation of ERK by MEK1 was inhibited or not. Results obtained from these four simulations are shown on Figure 3b, and demonstrate that nitric oxide is produced only when LPS activates the regulatory network, while the inhibitor does not influence results. The maximal flux through R_NOS agrees with the value of 0.0399 mmol/gDW/h reported in original publication, thus verifying SBML import of the GSMN model to JyMet. We note that while in this Use Case, the regulatory network regulates only one enzyme, this is an example of major global metabolic reprogramming. The production of large amounts of nitric oxide in response to pathogen requires both precursors and energy, and the GSMN model accounts for stoichiometry of all reactions linking medium nutrients to metabolic output. Moreover, the GSMN assures that the cell satisfies other metabolic demands, such as the demand for biomass production whereby the GSMN model accounts for global stoichiometry of providing cellular components and maintaining energy during induced nitric oxide production.

To compare the model predictions with the experimental data, we treated RAW264.7 macrophages with LPS and a MEK inhibitor and measured nitrate concentration in the medium. As nitrate can be produced only by the non-enzymatic conversion of nitric oxide from cells, and there is no nitrate consumption in the medium, concentrations are proportional to the nitric oxide production flux. Figure 3c shows that the model correctly predicts that LPS is obligate for the production of nitric oxide in RAW264.7 macrophages. However, the model did not predict the decrease in nitric oxide production caused by MEK inhibition. To explore this inconsistency, we used the interactive network visualization available in JyMet (Figure 3a) to examine example FBA solutions. Multiple pathways lead to *iNOS* activation, some of which are not dependent on MEK. In the model, the ‘iNOS’ substance representing activity of the *iNOS* gene is produced by three reactions representing the activity of ERK1/2-, HIF1- and JNK-dependent regulation of iNOS gene expression. Each of these regulators is, activated by different upstream signaling cascades. We used JyMet to interactively simulate and visualize different scenarios and concluded that the experimental results can be replicated if the following assumptions are made: (i) ERK induces a more potent activation of the *iNOS* gene than JNK and HIF1 (ii) MEK1 is a more potent ERK kinase than PKC. These assumptions were introduced to the model by setting flux bounds of (0, 0.005) for the transitions ‘JNK-> iNOS’, ‘HIF1->iNOS’ and ‘PKC_a_b=ERK1/2’ (Figure 3a, right panel). The upper bound is arbitrary, and selected to ensure that the flux towards ‘iNOS’ via ‘HIF1’ or ‘JNK’ reaches only a fraction of value required for maximal activation of R_NOS, with the remaining activation occurring via ERK1/2 regulation. This refined model is now able to reproduce the decreased, but not complete inhibition, of nitric oxide production by a MEK inhibitor (Figure 3d). A full description of this cycle of prediction, experimental testing and model refinement is presented as Supplementary Information (page 11), detailing how MUFINS and JyMet aid the iterative refinement cycle required during model development. These data support the assumptions above being one possible mechanistic solution to reproduce the observed biological phenotype. However, we note that further experimental confirmation is required to confirm the predicted biological insight. In a full iterative cycle of prediction, experiment and model refinement, multiple molecular targets would be subject to independent experiment verification before the model was validated. Here, we show one full cycle of simulation and experiment to demonstrate how the JyMet interface is used in this iterative model development process.

To summarize, we present the first linear model for steady-state simulation of networks integrating signaling, gene regulation and whole-cell metabolism in a mammalian cell. Moreover, we present the first simulation of perturbation of a global metabolic output by a signaling network inhibitor, and demonstrate this is consistent with experimental data. The ability to formulate hypotheses in terms of continuous ‘regulatory strength’ is demonstrated. This offers significant advantages over MILP-based approaches such as SR-FBA,^32^ where the regulatory network is used exclusively to formulate Boolean, on/off constraints. Finally, the graphics user interface JyMet allows an interactive exploration of combined signaling and metabolic flux distributions that is easily approachable by non-specialists. Together, these tools provide an ideal platform for non-specialists to generate mechanistic hypotheses based upon the interaction of gene and signal regulatory networks with genome-scale metabolism. These hypotheses can then drive experimental testing, enhancing our ability to identify novel biological insights.

### Use Case 2: kinetic model of cortisol signaling integrated with dFBA simulation of human GSMN

An important challenge in computational biology is the generation of large-scale models that are able to reproduce diverse biological functions. One approach to achieve this aim is the integration of validated small models (or modules) to form larger networks. A major consideration in such integration is the ability to combine models across different modeling formalisms and biological scales. In this Use Case, we demonstrate the utility of MUFINS for the generation and simulation of such multi-formalism, multi-scale models.

**Figure 4:**
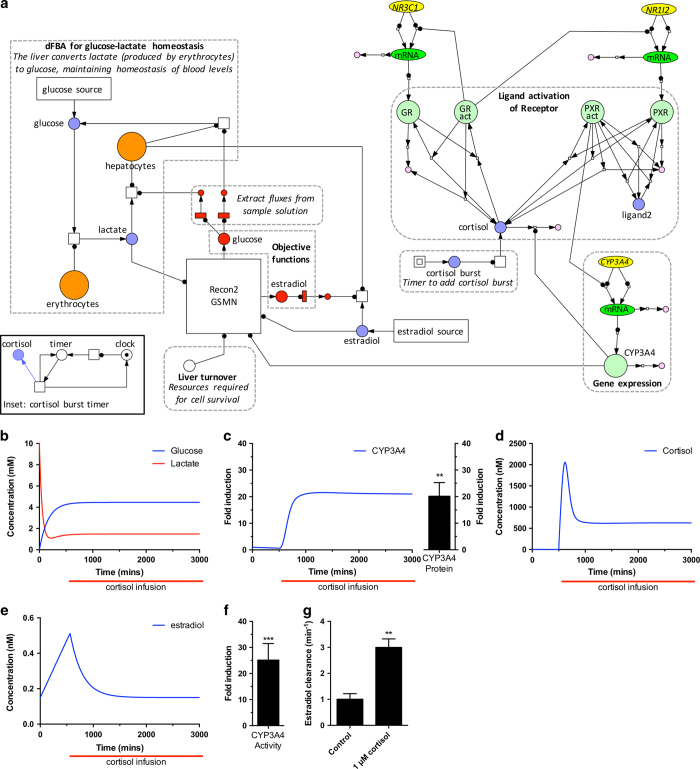
Multi-formalism simulation integrating cortisol signaling with the human Recon2 GSMN reveals a drug interaction with estradiol clearance. (**a**) The Petri Net diagram of network connectivity created in the Snoopy editor, with overlaid comments for clarity. Color and symbol size has been manually set to match SBGN molecule types and transition types specific to QSSPN. The PN connectivity to implement a timer for administering a network perturbation (cortisol burst), is contained within a coarse transition and shown as an insert. (**b**) Simulation of glucose and lactate dynamics in the blood physiological compartment, demonstrating a convergence to physiologically realistic steady states. Perturbation of the system through a simulated cortisol infusion starting after 500 min elicits a dynamic alteration in the signaling network, resulting in (**c**) a predicted increase in CYP34A protein levels, which is confirmed in primary human hepatocytes. The increased expression of CYP3A4 protein is predicted to increase flux through reactions catalyzed by this enzyme, leading to: (**d**) degradation of excess cortisol and establishment of new steady state; (**e**) a drug–drug interaction for a second CYP3A4 substrate (estradiol), contained within the GSMN, leading to a decrease in it’s steady-state level. The predicted increase in CYP3A4 activity following cortisol exposure is confirmed in primary human hepatocytes (**f**), as is the enhanced rate of estradiol clearance (**g**). FBA, Flux Balance Analysis; GSMN, Genome-Scale Metabolic Network; mRNA, messenger RNA; QSSPN, Quasi-Steady State Petri Net. **P<0.01, ***P<0.001.

Cortisol acts as an important signaling molecule within the body, with roles in circadian biology and the response to stress episodes. The level of cortisol in the body is interpreted at the cellular level through interaction with three nuclear receptors: the glucocorticoid receptor; the mineralocorticoid receptor; the pregnane-X receptor. These signals are integrated and produce a global metabolic shift corresponding to the current cortisol level. To reproduce such a complex biological phenomenon, it is necessary to combine multiple signaling cascades with genome-scale reconstructions of metabolism. Here, we demonstrate the application of MUFINS for such a multi-formalism simulation, integrating a detailed kinetic model of cortisol signaling in the liver, a genome-scale model of liver metabolism, and an ODE model of glucose and lactate dynamics in the blood (Figure 4). This is the first simulation integrating a human GSMN, physiological level ODE model and detailed kinetic model of an intracellular regulatory network. Thus, this Use Case demonstrates that MUFINS provides a unique tool for the integration of PBPK models with the mechanistic models of molecular networks operating in mammalian tissues.

The model is depicted in Figure 4a. We represent our previously published kinetic model of cortisol signaling in liver^33,34^ as a Petri net (PN) using Snoopy software.^25^ Size and color of place/transition symbols was used to mirror Systems Biology Graphical Notation^35^ molecule types as well as QSSPN specific place/transition types. The PN transition rates were defined using the ODE terms of the kinetic model. The PN places represent molecular concentrations. This dynamic model of cortisol signaling was linked to the human GSMN Recon2, with the CYP3A4 enzyme used as the QSSPN constraint place. The details of the cortisol signaling model and its coupling to GSMN are available in Supplementary Information (page 16).

To model whole-cell metabolism of hepatocytes, we used the community-based Recon2 GSMN.^36^ This model incorporates liver-specific reactions from the HepatoNet1,^37^ but is a much more extensive reconstruction of cellular metabolism. Exchange fluxes were constrained using the HepatoNet1 Physiological Import and Export set. The objective function was set to glucose regeneration from lactate, a major physiological function of the liver, where blood glucose and lactate concentrations are ODE variables. The dFBA simulation is implemented using place and transition within the QSSPN, rather than coded as a separate approach. We have further capitalized on the flexibility of the PN representation to create a timer administering a cortisol infusion after 500 min. This demonstrates how multi-formalism simulations in MUFINS can include complex, time-dependent perturbations to the model such as boluses or cell division events. For clarity, the timer is contained within a coarse transition, with the sub-level shown as an inset to Figure 4a. A detailed description of QSSPN place and transition types is given as Supplementary Information (page 27), along with a detailed description of model construction (page 12).

Simulations of the systems response to cortisol infusion, plus experimental confirmation are shown in Figures 4b–g. As shown in Figure 4b, the glucose and lactate concentrations converge to their physiological levels of 4.45 and 1.48 mM, respectively^38^ and are maintained during cortisol infusion from 500 min onwards. Cortisol infusion from 500 min produces a number of effects, which are mediated through activation of the two cognate nuclear receptors for cortisol within the regulatory network, PXR and GR.^39,40^ The cortisol-mediated activation of PXR results in a predicted increase in expression of the CYP3A4 enzyme (Figure 4c), which is experimentally confirmed at the transcript, protein and activity levels *in vitro* using primary human hepatocytes (Figure 4d). CYP3A4 is one of the main enzymes responsible for the metabolic clearance of cortisol; thus, the combination of a constant infusion of cortisol, followed by increased metabolism, results in an elevated blood cortisol level (Figure 4e). We note that the transition to this new blood cortisol level is not instant, demonstrating a concentration spike consistent with the time delay caused by the *de novo* production (i.e., transcription and translation) of CYP3A4 protein. Finally, in Figure 4f, we demonstrate that the cortisol infusion propagates through the signaling and metabolic networks, leading to predicted changes in blood concentrations for other chemicals. Estradiol is an endogenous hormone important in a range of biological functions, including development of the secondary sexual organs in both sexes and proliferation during the menstrual cycle and pregnancy in women.^41,42^ It has considerable clinical application, most notably as a contraceptive, either as the native compound or synthetic derivatives.^43^ As shown in Figure 4e, following the cortisol infusion, predicted levels of blood estradiol drop rapidly, decreasing approximately by one-half within 500 min. This lower level is maintained throughout the period where CYP3A4 protein levels are elevated. To confirm this effect, we have measured the clearance rate for estradiol in naive primary human hepatocytes and compared it with hepatocytes pre-exposed to 1μM cortisol, demonstrating enhanced clearance in cortisol-exposed hepatocytes (Figure 4g). In addition, we note that activation of PXR has previously been linked with a number of drug–drug interactions with estrogens, demonstrating the extrapolation of these predictions to the clinical setting.^40,44^ It is important to note that estradiol concentration was not a variable of the detailed kinetic model, and was rather identified as a variable of interest by examination of perturbed GSMN fluxes. This demonstrates how integration of detailed kinetic models with GSMNs can lead to the identification of interactions of biological interest. As such, this approach has much potential for the prediction of clinically relevant drug-induced disruption of homeostasis, and drug–drug interactions.^40,44^

In summary, this Use Case demonstrates the utility of MUFINS to combine legacy models developed in different formalisms and link molecular network knowledge to quantitative data on substance concentration at physiological level. As such, MUFINS represents the first software to allow such multi-scale, multi-formalism simulations through a GUI approachable to the non-specialist.

### Use Case 3: analysis of a clinical transcriptome data to understand *in vivo* tumor metabolism

An important branch of CBM methodology^5^ is dedicated to using ~omics data to create tissue and/or condition specific GSMNs. MUFINS is equipped with state-of-the-art CBM methods in this area; in addition, we have developed Fast iMAT, a new variant of the iMAT approach that is applicable to large ~omics sample numbers, where iMAT becomes impractical. We recently reported a preliminary version of Fast iMAT,^45^ dedicated to the analysis of expression data discretized to two states (absent or present transcript). Personalized GSMNs for 2,000 breast tumors were generated, identifying a low prognosis cluster with active serotonin production—an important biological insight.^45^ The first distribution version of MUFINS provides a mature version of the Fast iMAT algorithm. To demonstrate its utility, we analyze 262 previously unexamined paired clinical transcriptome samples.^46^ We demonstrate the significant upregulation of kyneurenine synthesis in tumor compared with normal breast tissue, and important pro-survival phenotype.^47,48^

## Comparison of MUFINS with existing tools

A comparison of MUFINS with existing tools is presented as Supplementary Information. We conclude that MUFINS is currently the only software supporting integration of (i) exact stochastic, (ii) ODE, (iii) qualitative dynamic, (iv) logical steady state and (v) CBM models in a general software platform with GUI. The only alternative to achieve integration of this range of formalisms is coding of the model in mathematical modeling environment. Although this strategy has achieved success,^13^ it is not a plausible proposition for non-specialists who lack programming skills. Moreover, multi-formalism modeling in a mathematical language involves the implementation of a simulation algorithm dedicated to each model. In MUFINS, each model is run in the same QSSPN simulation algorithm, with multi-formalism functionality emerging from the interactions between the different types of Petri Net places and transitions that can be graphically assembled, leading to a combinatorial diversity of types of models that can be simulated. Using one algorithm and a few well-defined place and transition types provides clearer control and description of model assumptions than coding a different main simulation loop for each model. Also, the algorithms available in MUFINS are validated and optimized against the legacy of previous applications, while formulation, validation and description of a simulation algorithm dedicated to a particular model will take additional time. It is our experience that even scientists who can program will find it easier to implement complex multi-formalism models by connecting QSSPN places and transitions to off-the-shelf GSMN models imported to JyMet, rather then by the development of dedicated mathematical modeling code. Moreover, MUFINS provides a wide range of CBM methods that can be used to model GSMNs before their integration with dynamic models. As Supplementary Information, we perform the largest review of CBM methods conducted to date (165 methods and 30 software packages), demonstrating that MUFINS is the second most general CBM software after COBRA toolbox^49^ in terms of the number of methods implemented. However, it provides the largest number of CBM methods under GUI with interactive network visualization. Finally, all CBM methods can now be applied to models formulated with inhibitor and activator constraints, which again enables execution of new CBM protocols without the need of coding (e.g., iMAT applied to models involving steady-state regulatory network).

## Future directions

We will continue to develop MUFINS towards improved interoperability with other tools and model databases, a key for model integration. Although currently QSSPN can be simulated only in MUFINS, definition of this multi-formalism framework in SBML will motivate the development of alternative tools. As shown in Figure 1, QSSPN models can currently be exported into two separate SBML files representing the CBM and PN parts of the model. We intend to represent QSSPN lookup tables, reset transitions and flux monitors with existing SBML objects, or to develop a bespoke SBML package. Furthermore, we will work towards improving integration of SBML files imported from public repositories into multi-formalism models in the JyMet GUI. This will involve further work on network visualization in JyMet, providing a graph editor dedicated to connecting different mechanistic models by common variables. We also plan to develop interoperability between MUFINS and Garuda (http://www.garuda-alliance.org) to make full use of our multi-formalism simulation tool within this established alliance of systems biology software. This will be facilitated by design of our software (Figure 1) providing stand-alone simulation engines ideal for embedding in different interfaces.

### Conclusions

The multi-scale nature of complex biological systems is currently the major challenge preventing their computational understanding. A number of theoretical frameworks have achieved spectacular successes in mechanistically modeling different levels of cellular organization such as metabolic, signaling and gene regulatory networks. However, in a real cell, all these processes proceed simultaneously, and without multi-scale simulation the insight and predictive power provided by models will be limited. We present MUFINS, the first general software addressing this multi-formalism simulation challenge. Novel algorithms available in MUFINS provide solutions for three major technological challenges: (i) integration of CBM and hybrid stochastic/deterministic dynamic simulation; (ii) CBM of integrated signaling/metabolic models; (iii) analysis of large clinical transcriptome studies in the context of GSMN. This is demonstrated through three Use Cases, where we simulate models of mammalian systems composed of: GSMNs, logical hypergraph models of signaling, kinetic models of gene regulation and PBPK models. We experimentally validate model predictions and show how our software can aid experimental scientists through an iterative cycle of hypothesis generation, experimentation and model refinement. Because the need for a multi-scale, multi-formalism approach is currently most recognized in the context of Personalized Medicine and Quantitative Systems Pharmacology, we focused our Use Cases on mammalian cells. However, mechanistic simulation is a major tool in Synthetic Biology, where MUFINS will be ideal to integrate detailed models of genetic circuits with GSMNs and further extend molecular cell factory models to include bioreactor mass transfer. Therefore, we believe that multi-formalism simulation with MUFINS will find broad application in mechanistic modeling of biological systems.

### Software availability

MUFINS is free, open source software available under GNU GPL license from:

MUFINS home page: http://sysbio3.fhms.surrey.ac.uk/mufins/

GitHub repository: https://github.com/kierzek/MUFINS

## Figures and Tables

**Table 1:** Summary of simulation formalisms applied in Use Cases

*Use Case*	*Formalisms*	*Reference*
Use Case 1: linear inhibitory constraints	CBM, logical hypergraph, inhibitor and activator constraints	This work
Use Case 2: integration of regulatory networks and GSMNs	CBM, ODE, Gillespie, physiological compartment models	This work and ref. 9
Use Case 3: prediction of metabolic landscapes	CBM, congruency approach to analysis of ~omics data in the context of GSMNs	This work and ref. 45

Abbreviations: CBM, constraint-based modeling; GSMN, Genome-Scale Metabolic Network; ODE, Ordinary Differential Equation.

## Notes

Supplementary Information

GETTING_STARTED
