# Supplementary material for: MUFINS: multi-formalism interaction network simulator
Source: NPJ Syst Biol Appl. 2016 Nov 17;2:16032–. doi: 10.1038/npjsba.2016.32 (PMC5516860; doi:10.1038/npjsba.2016.32)
Supplement: Supplementary Information [file npjsba201632-s1.doc]

# Supplementary Information

**MUFINS: Multi-Formalism Interaction Network Simulator**

Huihai Wu^1^, Axel von Kamp^2^, Vytautas Leoncikas^1^, Wataru Mori^3^, Nilgun Sahin^4^, Albert Gevorgyan^5^, Catherine Linley^1^, Marek Grabowski^6^, Ahmad A. Mannan^1^, Nicholas Stoy^1^, Graham R. Stewart^1^, Lara T. Ward^7^, David J.M. Lewis^1^, Jacek Sroka^6^, Hiroshi Matsuno^3^, Steffen Klamt^2^, Hans V. Westerhoff^4,8,9^, Johnjoe McFadden^1^, Nicholas J. Plant^1,*^, Andrzej M. Kierzek^1,10*^

*corresponding authors, [a.kierzek@surrey.ac.uk](mailto:a.kierzek@surrey.ac.uk), [n.plant@surrey.ac.uk](mailto:n.plant@surrey.ac.uk)

E-mail addresses: Huihai Wu ([h.wu@surrey.ac.uk](mailto:h.wu@surrey.ac.uk)); Axel von Kamp ([vonkamp@mpi-magdeburg.mpg.de](mailto:vonkamp@mpi-magdeburg.mpg.de)); Vytautas Leoncikas ([v.leoncikas@surrey.ac.uk](mailto:v.leoncikas@surrey.ac.uk)); Wataru Mori ([porepole8@gmail.com](mailto:porepole8@gmail.com)); Nilgun Sahin ([nilguenyilmaz@gmail.com](mailto:nilguenyilmaz@gmail.com)); Albert Gevorgyan ([gevorgyana@MedImmune.com](mailto:gevorgyana@MedImmune.com)); Catherine Linley ([c.linley@surrey.ac.uk](mailto:c.linley@surrey.ac.uk)); Marek Grabowski ([grmarek@gmail.com](mailto:grmarek@gmail.com)); Ahmad A. Mannan ([a.mannan@abdn.ac.uk](mailto:a.mannan@abdn.ac.uk)); Nicholas Stoy ([n.stoy@surrey.ac.uk](mailto:n.stoy@surrey.ac.uk)); Graham R. Stewart ([g.stewart@surrey.ac.uk](mailto:g.stewart@surrey.ac.uk)); Lara T. Ward ([lara.ward@AstraZeneca.com](mailto:lara.ward@AstraZeneca.com)); David J. M. Lewis ([d.j.lewis@surrey.ac.uk](mailto:d.j.lewis@surrey.ac.uk)); Jacek Sroka ([j.sroka@mimuw.edu.pl](mailto:j.sroka@mimuw.edu.pl)); Hiroshi Matsuno ([matsuno@sci.yamaguchi-u.ac.jp](mailto:matsuno@sci.yamaguchi-u.ac.jp)); Steffen Klamt ([klamt@mpi-magdeburg.mpg.de](mailto:klamt@mpi-magdeburg.mpg.de)); Hans V. Westerhoff ([Hans.Westerhoff@manchester.ac.uk](mailto:Hans.Westerhoff@manchester.ac.uk); H.V.Westerhoff@uva.nl); Johnjoe McFadden ([j.mcfadden@surrey.ac.uk](mailto:j.mcfadden@surrey.ac.uk)); Nicholas J. Plant ([n.plant@surrey.ac.uk](mailto:n.plant@surrey.ac.uk)); Andrzej M. Kierzek ([a.kierzek@surrey.ac.uk](mailto:a.kierzek@surrey.ac.uk));

Institutional addresses: 1) School of Biosciences and Medicine, Faculty of Health and Medical Sciences, University of Surrey, Guildford, GU2 7XH, United Kingdom. 2) Max Planck Institute for Dynamics of Complex Technical Systems, Magdeburg, Germany. 3) Graduate School of Science and Engineering & Faculty of Science, Yamaguchi University, Yoshida, Yamaguchi 753-8512, Japan 4) Molecular Cell Physiology, VU University Amsterdam, Amsterdam, The Netherlands 5) MedImmune Cambridge, CB21 6GH, United Kingdom 6) Institute of Informatics, University of Warsaw, Warsaw, Poland 7) Oncology DMPK, AstraZeneca, Alderley Park, Cheshire, SK10 4TG, United Kingdom 8) Manchester Centre for Integrative Systems Biology, University of Manchester, Manchester, United Kingdom, 9) Synthetic Systems Biology, Netherlands Institute for Systems Biology, University of Amsterdam, Amsterdam, The Netherlands. 10) Simcyp Limited (a Certara Company), Sheffield, UK

Table OF CONTENTS

EXPANDED USE CASES [4](#__RefHeading___Toc457283025)

Use case 1: Whole-cell metabolic reprogramming by signaling and gene regulatory networks in the mammalian macrophage [4](#__RefHeading___Toc457283026)

General Introduction [4](#__RefHeading___Toc457283027)

Methods [4](#__RefHeading___Toc457283028)

1.1 Logical hypergraph model of macrophage signalling [4](#__RefHeading___Toc457283029)

1.2 Translation of the logical hypergraph model to a stoichiometric model with linear inhibitor constraints [5](#__RefHeading___Toc457283030)

Figure S1: Logical hypergraph conversion to MUFINS reaction formulas. [6](#__RefHeading___Toc457283031)

1.3 Definition of external metabolites in the linear signaling model [6](#__RefHeading___Toc457283032)

1.4 Simulation of the model [7](#__RefHeading___Toc457283033)

1.5 Pre-processing of the linear signaling model [7](#__RefHeading___Toc457283034)

Figure S2: Example problem files in JyMET [8](#__RefHeading___Toc457283035)

1.6 Visualization of simulation results within the JyMet GUI [9](#__RefHeading___Toc457283036)

1.7 Nitrate measurements in RAW264.7 cell line [10](#__RefHeading___Toc457283037)

Figure S3: Selection of non-zero flux rates for visualization [10](#__RefHeading___Toc457283038)

Figure S4: Example visualization of FBA results using JyMet. [11](#__RefHeading___Toc457283039)

Results [11](#__RefHeading___Toc457283040)

1.8 Iterative model refinement through comparison with experimental data [11](#__RefHeading___Toc457283041)

1.8.1 Prediction of NO production in human macrophages in silico [12](#__RefHeading___Toc457283042)

Figure S5: Initial Model Predictions [12](#__RefHeading___Toc457283043)

1.8.2 Experimental determination of NO production in RAW264.7 murine macrophages in vitro [12](#__RefHeading___Toc457283044)

Figure S6: Nitrate production of RAW264.7 macrophages [13](#__RefHeading___Toc457283045)

1.8.3 Refinement of the in silico model based upon experimental observations. [13](#__RefHeading___Toc457283046)

Figure S7: Refined model predictions [14](#__RefHeading___Toc457283047)

Use case 2: Kinetic model of cortisol signalling integrated with dFBA simulation of human GSMN [16](#__RefHeading___Toc457283048)

General Introduction [16](#__RefHeading___Toc457283049)

Figure S8: Overview of multi-formalism model of cortisol signalling in human liver. [16](#__RefHeading___Toc457283050)

Methods [17](#__RefHeading___Toc457283051)

2.1 Kinetic model of cortisol signaling [17](#__RefHeading___Toc457283052)

2.2 Preparation of continuous Petri Net representation of the kinetic model of cortisol signalling [17](#__RefHeading___Toc457283053)

2.3 Preparation of Recon2 human genome scale metabolic model [17](#__RefHeading___Toc457283054)

2.4 Integration of cortisol signaling model with the Recon2 human GSMN [18](#__RefHeading___Toc457283055)

Figure S9: Mapping of protein levels to reaction activity status for QSSPN simulation [19](#__RefHeading___Toc457283056)

2.5 Simulation of physiological compartment dynamics for glucose, lactose and estriadol [19](#__RefHeading___Toc457283057)

Figure S10: Generation of physiological compartment dynamics for QSSPN simulation [21](#__RefHeading___Toc457283058)

2.6 Simulation of physiological compartment levels for estradiol [21](#__RefHeading___Toc457283059)

2.7 Use of biomass constraint to represent cell turnover with an organ [21](#__RefHeading___Toc457283060)

2.8 Parameter values used within the model [21](#__RefHeading___Toc457283061)

Table S1. New parameter values used in Use case 2. [22](#__RefHeading___Toc457283062)

2.9 Preparation of control file for QSSPN [22](#__RefHeading___Toc457283063)

Figure S11: Generation of the control file for QSSPN simulation [23](#__RefHeading___Toc457283064)

2.10 Simulation of complete model [23](#__RefHeading___Toc457283065)

2.11 Treatment of primary human hepatocytes with cortisol. [24](#__RefHeading___Toc457283066)

2.12 Determination of CYP3A4 expression and activity levels [25](#__RefHeading___Toc457283067)

2.13 UPLC-MS detection of estradiol in cell lysates and conditioned medium [25](#__RefHeading___Toc457283068)

Use Case 3: Analysis of a clinical transcriptome study to understand in vivo tumour metabolism [27](#__RefHeading___Toc457283069)

General Introduction [27](#__RefHeading___Toc457283070)

Methods [27](#__RefHeading___Toc457283071)

3.1. Metabric datasource [27](#__RefHeading___Toc457283072)

3.2. Transcriptomic data pre-processing [28](#__RefHeading___Toc457283073)

Figure S12: Overview of fast iMAT analysis of metabolic reprogramming in cancer and healthy breast tissue [28](#__RefHeading___Toc457283074)

3.3. Prediction of metabolic landscapes [28](#__RefHeading___Toc457283075)

3.4. Identification of differentially expressed genes and functional classification [29](#__RefHeading___Toc457283076)

Results [29](#__RefHeading___Toc457283077)

Figure S13: Breast tumours have significantly higher flux toward kyneurenine that matched normal breast tissue. [30](#__RefHeading___Toc457283078)

TECHNICAL DESCRIPTION OF COMPUTATIONAL METHODS [31](#__RefHeading___Toc457283079)

Linear Inhibitor and Activator Constraints [31](#__RefHeading___Toc457283080)

Extended Quasi Steady State Petri Net (QSSPN) algorithm. [32](#__RefHeading___Toc457283081)

2.1. DEFINITION OF QUASI-STEADY STATE PETRI NET (QSSPN). [32](#__RefHeading___Toc457283082)

2.1.1. Petri Nets. [32](#__RefHeading___Toc457283083)

2.1.2. Flux Balance Analysis (FBA) [34](#__RefHeading___Toc457283084)

2.1.3. Quasi Steady-State Petri Nets (QSSPN) [35](#__RefHeading___Toc457283085)

2.2 QSSPN SIMULATION ALGORITHM [36](#__RefHeading___Toc457283086)

2.2.1 Pre-place activity. [36](#__RefHeading___Toc457283087)

2.2.2 Propensity function. [36](#__RefHeading___Toc457283088)

2.2.3 Transition types. [37](#__RefHeading___Toc457283089)

2.2.4 Setting of flux bounds (Function: setQSSFbounds()). [38](#__RefHeading___Toc457283090)

2.2.5 Evaluation of an objective function (Function: evaluateObjective()) [38](#__RefHeading___Toc457283091)

2.2.6 Marking of objective place (Function: updateObjectivePlace()) [38](#__RefHeading___Toc457283092)

2.2.7. Firing of reset transitions (Function: fireResetTransitions() new feature). [39](#__RefHeading___Toc457283093)

2.2.8. Firing of immediate transitions (Function: fireImmediateTransitions()). [39](#__RefHeading___Toc457283094)

2.2.9. Firing of continuous transitions (Function: fireContinuousTransitions(Δt_max_)). [39](#__RefHeading___Toc457283095)

2.2.10. Firing of delayed transitions (Function: fireDelayedTransition(t)). [39](#__RefHeading___Toc457283096)

2.2.11. Scheduling of delayed transitions (Function: scheduleDelayedTransition(m)) [40](#__RefHeading___Toc457283097)

2.2.12. Firing of flux transitions (Function: fireFluxTransitions()). [40](#__RefHeading___Toc457283098)

2.2.13 QSSPN hybrid simulation algorithm. [41](#__RefHeading___Toc457283099)

2.3. DISTRIBUTED QUALITATIVE SIMULATION WITH QSSPNclient. [42](#__RefHeading___Toc457283100)

Fast iMAT algorithm [42](#__RefHeading___Toc457283101)

COMPARISON OF TOOLS AVAILABLE WITHIN MUFINS WITH EXISTING SOFTWARE [45](#__RefHeading___Toc457283102)

Multi-formalism simulation in MUFINS [45](#__RefHeading___Toc457283103)

Comparison with major software [46](#__RefHeading___Toc457283104)

Table S2. Comparison of CBM features of MUFINS with other software. [47](#__RefHeading___Toc457283105)

References [48](#__RefHeading___Toc457283106)

# EXPANDED USE CASES

# Use case 1: Whole-cell metabolic reprogramming by signaling and gene regulatory networks in the mammalian macrophage

## General Introduction

The integration of gene and signalling regulatory networks with GSMNs requires the ability to incorporate stimulation and inhibitory signals into the reconstruction. It has long been recognized that activation of a signalling network by post-translational modifications can be modelled as a flux distribution constrained by a stoichiometric matrix (1). In this use case, we demonstrate the extension of this approximation to all activation edges in the hypergraph, including gene activation by transcription factor binding and activation of cellular responses. Representation of inhibition in the CBM framework has always been a challenge, with proposed solutions generally being computationally expensive methods based on MILP (2). In this use case we demonstrate an extension of the approach of Vardi and colleagues (3), representing inhibition by linear constraints enforcing a reciprocal relation between inhibitor production and inhibited flux. In the MUFINS implementation, an inhibited reaction is limited by the sum of irreversible reaction fluxes producing the inhibitor, rather than by a virtual flux connecting inhibitor to external metabolite without flux balance constraints.

## Methods

Complete preparation of the model is provided as a step-by-step description in the following sections. Additional tutorials for model simulation in the JyMet GUI are provided in the help documents associated with the software distribution (MUFINS_Docs🡺GETTING _STARTED). The final model is available within the MUFINS software distribution (MUFINS_Examples🡺RAW264_7_r).

### Logical hypergraph model of macrophage signalling

The signalling network applied in this use was developed to study the host-pathogen interaction between macrophages and *Mycobacterium tuberculosis* during infection. It is based on two large-scale signalling maps: the toll-like receptor (TLR) network from Oda and Kitano (4); the integrated map of macrophage pathogen recognition and effector systems by Raza et al. (5). Both maps were constructed by manual curation of the literature and hence represent robust reconstructions of the known biology. The signalling map reconstructed by Raza et al. (5) is cell-type specific, and hence more relevant to the current reconstruction. In addition, to information from both large-scale signalling maps, information from 243 articles was collated to extend the model with regard to endocytosis, phagocytosis, apoptosis, necrosis, autophagy, eicosanoid signalling, vitamin D effects and the interference to these processes posed by *M. tuberculosis.* We note that compared to the networks from Oda and Kitano (4) and Raza et al. (5), the current model contains less mechanistic detail, allowing a more compact representation.

The reconstruction consists of 205 species connected by 286 interactions whose logic is captured by a hypergraph representation of the network (6). The model was reconstructed using the MATLAB toolbox CellNetAnalyzer (7), which is available from https://www2.mpi-magdeburg.mpg.de/projects/cna/cna.html. A complete list of all species and interactions, together with comments, are presented as Supplementary Table 1. The first tab contains information on each of the species within the network. In this worksheet, the first column provides the name of each species, which may represent proteins, other molecules, genes or processes. The second column includes curation data for these species: source(s) mentioning a given species are referred to by PubMed Identifier (PMID), “Oda” for Oda and Kitano (4) or “Raza”/“Raza 2” for the 2008 and 2010 versions of Raza et al. (5) model, respectively; where appropriate, common synonyms for a species are listed are listed; where appropriate SwissProt identifiers (starting with either “P” or “Q” followed by five digits) are given. The second tab of Supplementary Table 1 contains information on the interactions between species. The first column provides information on the interaction, which are are denoted in one of three ways: first, direct activations are indicated as normal reactions (e.g. akt1=bad, meaning akt1 activates bad); second, direct inhibitions are indicated through the symbol “!” meaning logical NOT (e.g. !akt1=gsk3b, meaning AKT1 inhibits GSK3B); third, an AND combination of species (e.g. !gsk3b+msk_1_2=creb, meaning that GSK3B must be inactive and MSK1/2 active in order for CREB to be active). When several interactions have the same species as their target then an OR-combination of the interaction states is taken to set the state of the target species. The second column of this worksheet contains curation data for each interaction: source(s) mentioning a given interaction are referred to by PubMed Identifier (PMID); comments on areas for further model expansion are also noted, where appropriate

### Translation of the logical hypergraph model to a stoichiometric model with linear inhibitor constraints

To integrate the signaling and metabolic networks, we have translated the logical hypergraph to a stoichiometric model with inhibitory constraints. In the logical hypergraph, inhibitory edges end at substance nodes (e.g.: akt1 inhibits tsc), meaning that an inhibition edge connecting substance A and B can be represented by introduction of A as inhibitor of every flux producing B. To convert the logical hypergraph to MUFINS reaction formulas, the logical rules were exported from CellNetAnalyzer as a text format document. This file can then be opened in several programs (e.g. Excel) and the text replace function used to change the formula format, replacing “!” with “~”, which is read by MUFINS as an inhibition. Export from Excel as a tab-separated file (File🡺Save as) will then create a MUFINS reaction table that can be opened with the JyMet GUI (File🡺Open Model). We note that a user can perform these steps without programming experience. An example of this conversion is provided in Figure S1.

###
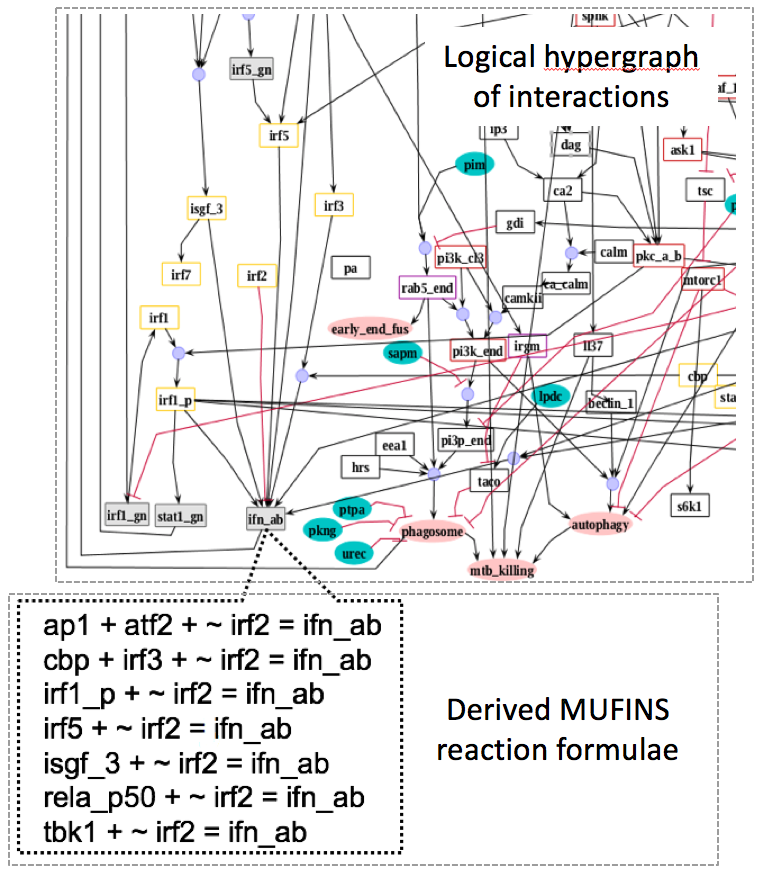
Figure S1: Logical hypergraph conversion to MUFINS reaction formulas.

All edges activating the node “ifn_ab” are represented as stoichiometric fluxes. This includes hyperedges representing the “AND” operator for joint action of “atf1 and ap2” as well as “cbp and irf3”. Subsequently, “irf2” inhibition of “ifn_ab” has been modeled by including “irf2” as an inhibitor of every reaction producing “ifn_ab”.

### Definition of external metabolites in the linear signaling model

In linear signaling models, external metabolites can represent both input signals (i.e. sources of signal flow) and effectors (i.e. sinks of signal flow). Within the use case model, external metabolites include molecular components by which *Mycobacterium tuberculosis* subverts macrophage defenses, and inhibitors used to experimentally perturb the signaling network. When the model is first opened in Jymet, the user is prompted to ‘Define the external metabolites’. All external metabolites are given a tag: For example, M_o2_e_xt defines this species are representing external oxygen. The external tag can be entered through “Solve🡺Externality tag”.

External metabolites are connected to the signaling model by a transport reaction, which we refer to as an input flux. To simulate the effect of a particular metabolic signal in the cellular environment, the corresponding input flux is set to (0, v_max_) if the signal is present, or (0, 0) if it is absent. In this use case a qualitative signaling model is presented, and the fluxes do not have physical units. Therefore, an arbitrary value of v_max_ = 100 was used. If quantitative data for reaction fluxes are available then these can be used to further constrain the model (see use case 2 for an example of constraint of the uptake of glucose). An example input flux can be seen by searching (Edit->Search) for the reaction “irf2_input” in the reactions tab. This reaction sets the input flux for the irf2 species, an inhibitor within the network. The bounds of the reaction are currently set to (0,0), meaning that no irf2 is present within the network, as the input flux is closed.

NOTE #1: if a search fails to identify “irf2_input”, then (i) ensure that all reactions are shown (View🡺Show🡺all), and (ii) select all reactions (ctrl-A or cmd-A) to ensure that you are not searching a sub-set of the reactions.

NOTE #2: For extracellular, but not intracellular inhibitors it is also required to introduce an efflux reaction, allowing mass balance to be achieved during flux balance analysis (FBA). This is achieved by setting input fluxes to (v_max_, v_max_) if the signal was present, or (0, 0) if it was absent. For intracellular reactions, bounds may be set as (0, v_max_).

Once the external metabolites have been set, the model can be saved (File🡺Save table). We recommend that when saving the tag .sfba is used rather than .txt, to denote that the file type more clearly.

### Simulation of the model

To simulate a model within JyMet, a problem file must be constructed, which sets the parameters for the simulation. At the simplest level, this problem file may contain only a single line, defining the objective function. For example, figure S2 shows a problem file to maximize (MAX!) the production of “M_no_e_xt”, the species that represents external nitric oxide. To enter an objective into a problem file, the species or reaction name is typed into the Objective box, a direction (max or min) selected, and then (Solve🡺Write) problem used to write the command. An additional command line containing “;” is automatically used to signify the end of the problem file. It is possible to enter multiple problems, each separated by a line containing “;”, and these will be solved sequentially.

More complex problem files can be constructed, containing, for example, commands to alter the bounds of reactions. To see an example of this use (File🡺Open Problem), and select “simulate.pfile”. This example problem file contains three problems, each of which maximizes for external nitric oxide, but with different levels of inhibitors and signaling molecules (Figure S2)

### Pre-processing of the linear signaling model

With external metabolites set as described above, the model was simulated using Flux Balance Analysis (FBA) in the JyMet GUI. The results of this simulation indicated a number of inhibitors (e.g. irf2) were not produced by any reaction in the system. These inhibitors were also assigned input and efflux reactions, treating them as external perturbations. After this, all inhibitors could be produced within the model.

To check for spurious activations in the model, JyMet was used to subject the final regulatory network model to Flux Variability Analysis (FVA) simulation with all input fluxes set to (0, 0). Twenty-seven reactions in the regulatory network were identified that could be active when there was no input. Examination of example FBA solutions demonstrated that these reactions participated in autocrine feedback loops involving TNF and IL1R, which can activate signaling pathways in the absence of other signals. As these are valid biological scenarios, there was no need to adjust the model to resolve these autocrine feedbacks.

We note that the optimization steps described above were relatively simple compared to the MILP-based optimization of the external metabolite set and depth-first search for spurious positive feedbacks that Vardi and colleagues (3) had to apply to pre-process their model. This is in part due to the high model quality of the input logical hypergraph, which was manually reconstructed and tested for consistency in CellNetAnalyzer (6). However, we also note that our modified formulation of inhibitory constraint does not introduce additional external metabolite for every inhibitor, thus simplifying model pre-processing.


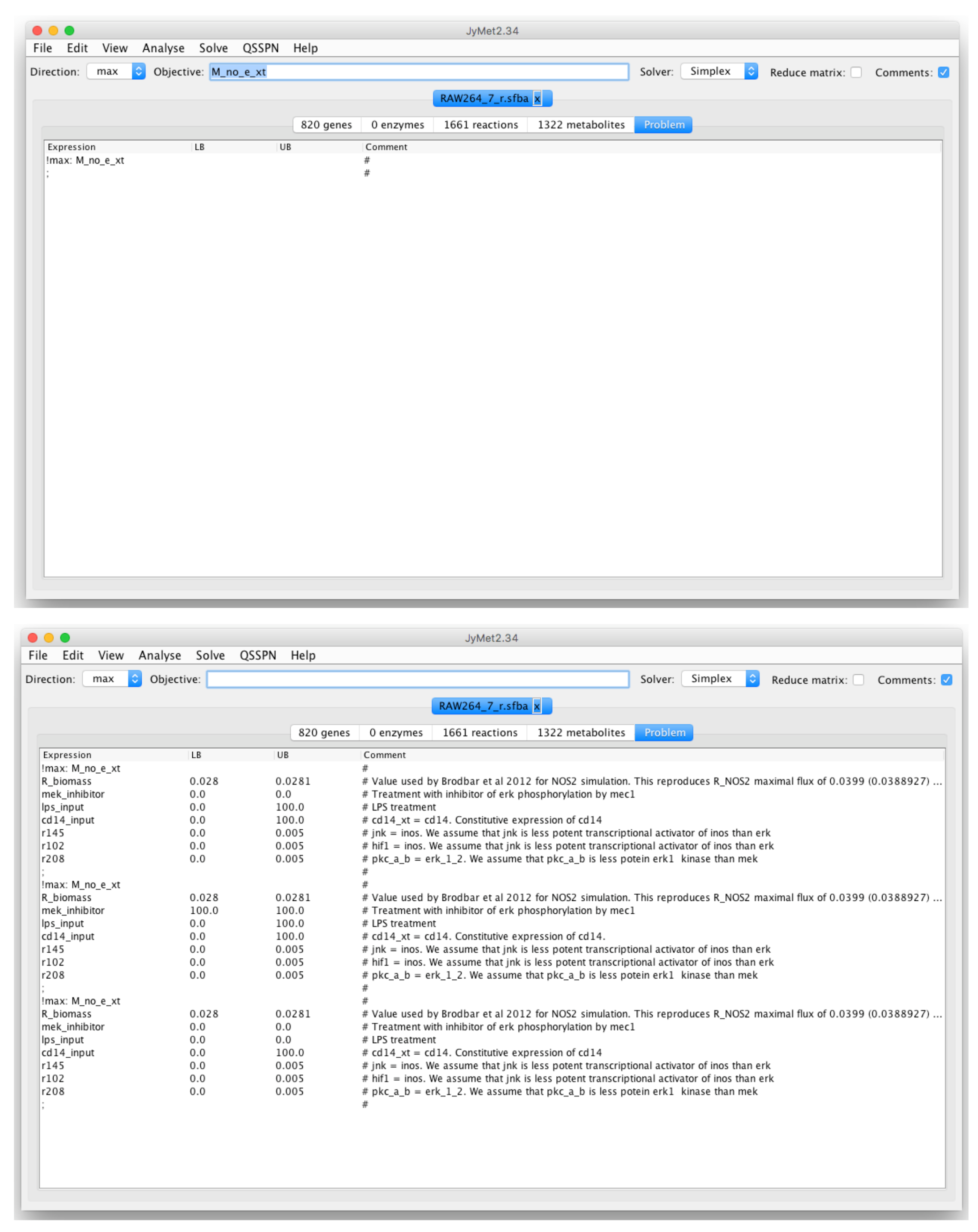


### Figure S2: Example problem files in JyMET

The top panel demonstrates a simple problem file, defining the objective function (M_no_e_xt) to be maximized (!max) during simulation. The lower panel demonstrates a more complex problem file specifying three separate problems to be solved sequentially. Each problem maximizes the same objective (M_no_e_xt), but with different combinations of inhibitors.

### Visualization of simulation results within the JyMet GUI

An important aspect of MUFINS is its presentation through JyMet, a graphical interface allowing more intuitive use for the non-specialist. One of the new features of JyMet, developed as part of MUFINS, is the ability to produce visualisations of regulatory networks, or parts therefore, and overlay these with simulation results (e.g. FBA flux values for visualised reactions). To demonstrate the utility of this we have used JyMet to visualise the section of the regulatory network that directly impinges on *iNOS* gene expression, and the subsequent production of external NO within the GSMN. An example FBA solution is overlaid, providing flux values for the individual reactions.

To undertake visualisation flux through the signalling network, first it is necessary to produce a simulation, the results of which can be overlain on the visualisation. Using the model and problem file previously described, an FBA simulation is undertaken by first setting the mode of analysis (Analyse🡺FBA) and the running the simulation (Solve🡺Solve). In this example, we will visualize the results from the first FBA, listed under “Record1” tab. Following simulation, those reactions within the signaling network need to be selected for visualization. A useful feature of JyMet is the comments sections, which can be used to tag reactions for later sorting. In this example, of reactions from the signaling network have the tag “@regnet”, and can be selected using the search option (Edit🡺Search🡺“@regnet”). As we are only interested in signaling reactions, which are active, the reactions can next be sorted by clicking on the “Transition rate” column, and only those with a non-zero flux selected (Figure S3).

There are a number of visualizations available within JyMet; figure S4 (left panel) provides a screenshot of a hierarchical layout for the selected reactions (View🡺Layout🡺Hierarchical). The network is visualized in Petri Net (bipartite graph) notation with rectangles representing transitions and circles representing places (Figure S4). Flux values are overlain, with line thickness also used to visualize fluxes. Right clicking on the visualization opens a pop-up window for zooming. This automatic layout can be manually adjusted, if desired, and saved (File🡺Save graph). Saved layouts can be reloaded through the command (View🡺Layout🡺Custom). When the layout is reloaded, JyMet identifies reactions in reaction tables and visualizes new simulation results. Thus, saved layouts can be used to quickly re-examine simulation results, and are an ideal tool for iterative examination of the effects of perturbation, and during model refinement.

We note that automatic layout in JyMet, even if manually adjusted, still does not produce a publication quality network diagram. First, there are constraints on molecular species names resulting from reaction formula syntax: spaces and special characters cannot be used. Also, the user cannot control the number display format, such as the number of significant digits. Finally, there are limits in graphical notation, colours and line shapes. However, such visualizations provide a template for the production of publication quality images. To illustrate these points, Figure S4 shows both a manually adjusted layout in JyMet (left panel) and a publication quality layout created in Snoopy Petri Net editor and further improved in MS Powerpoint (right panel). We note, that it would be very difficult and error-prone to create final figure based on results table alone as network connectivity would have to be created by examining reaction formulas rather than layout diagram, where it is already drawn. Improvement to visualization features towards generation of publication quality figures requires substantial effort and will be a subject of further development

In summary, network visualization within JyMet serves as tool for interactive examination of simulation results. We believe that quick, interactive visualization is key for model exploration, hypothesis generation and model refinement. Once results are examined and conclusions established, publication quality figures based upon these visualizations could then be created using dedicated desktop publishing software.

### Nitrate measurements in RAW264.7 cell line

The experimental results shown on Figure 3C of main manuscript have been obtained using the following protocol. The murine macrophage cell line RAW 264.7 was cultured in the presence of the MEK1 inhibitor, PD98059, to investigate the effect it had on the production of nitrate following stimulation with LPS. Briefly, RAW cells were plated out in 24 well plates at 0.5x10^6^ cells/ml and cultured overnight in DMEM medium (Sigma Aldrich, Poole, UK) supplemented with 10% FCS and 2mM glutamine. Subsequently the medium was replaced and the cells were cultured for an hour in the presence or absence of 4µl/ml of PD98059 (NEB, Hitchin, UK). Following this 10ng/ml of LPS (Enzo, Exeter, UK) was added for a further 24 hours before the culture media was collected and the level of nitrate present assessed using the Griess reagent system according to the manufacturer’s instructions (Promega, Southampton, UK). Readings were made on Emax precision microplate reader at 540nm (Molecular Devices, Wokingham, UK).


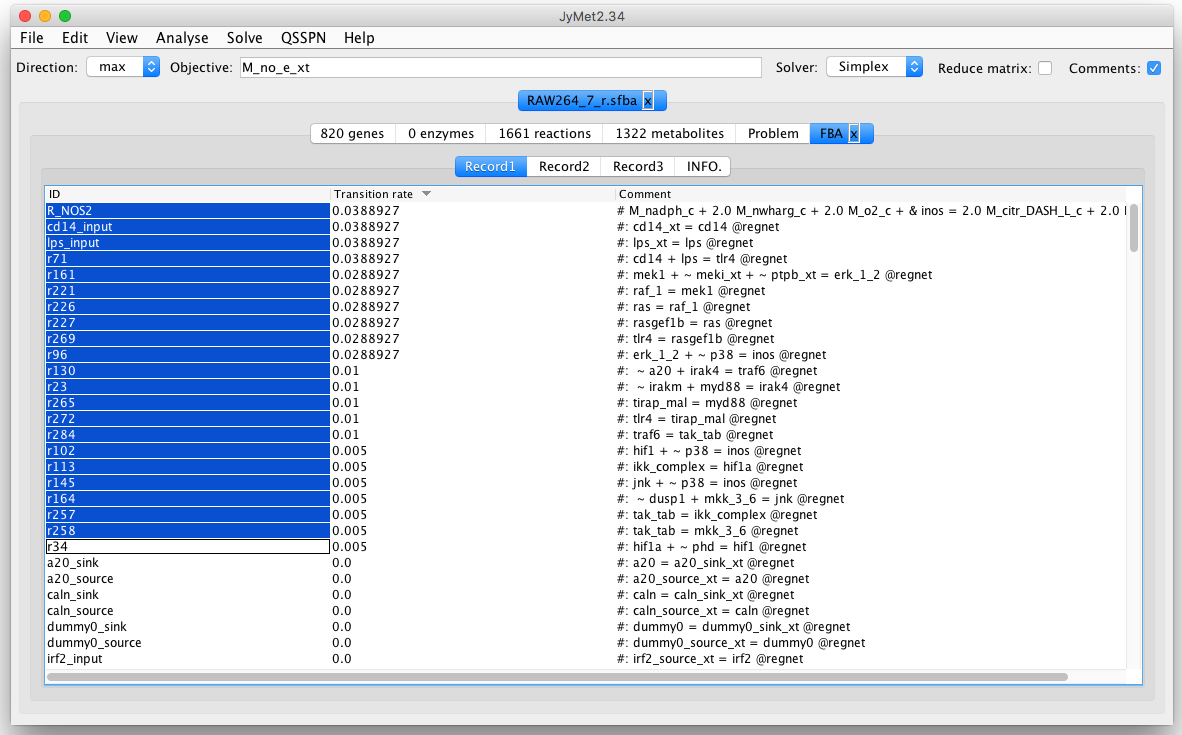


### Figure S3: Selection of non-zero flux rates for visualization


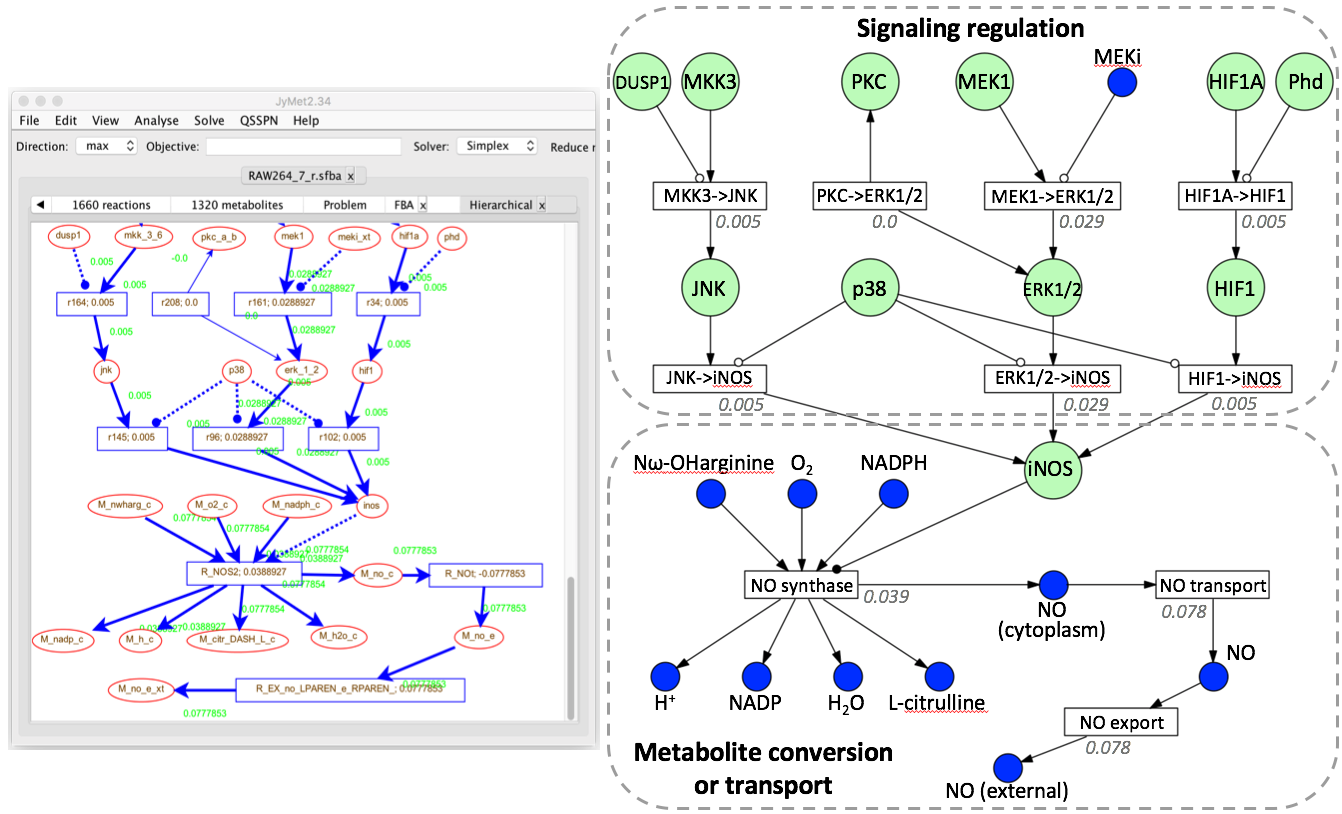


### Figure S4: Example visualization of FBA results using JyMet.

The left panel shows a screenshot of the JyMet interface, demonstrating on screen visualization of the reconstruction, created by automatic hierarchical layout with manual adjustment. Rectangles represent reactions and elipsoids molecular entities, which are joined by solid arrows to represent the direction of signal flow. Hatched lines are used to indicate regulatory signals, representing inhibition (circle end) or stimulation (arrow head). The right panel demonstrates a publication-quality figure of the relevant regulatory pathway for presentation of final conclusions. The visualization depicts where signalling pathways converge on the iNOS gene, which is required for nitric oxide production in the whole-cell stoichiometric model. Production of NO_external_ was set as the objective functions, and FBA undertaken. Flux rates for an example FBA solution are displayed on the network diagram; on the right panel flux rates in grey represent the overall transition flux rate while those in blue represent the flux rate for individual molecular species required to maintain stoichiometry.

## Results

### Iterative model refinement through comparison with experimental data

The interactive network visualisation implemented in JyMet is an ideal tool for iterative model refinement. It allows comparison of model predictions with experimental data, leading to model adjustments that improve the congruency between predicted and observed conditions. These changes to the model constitute mechanistic hypotheses, which can be used to direct future work towards understanding of the complex biological system under investigation. To illustrate this process, we have conducted one iteration within Use Case 1, whereby: i) simulation was used to generate a testable prediction ii) the prediction was experimentally examined iii) discrepancies between the simulation and observation were used to generate new hypothesis, which could direct further experimental work. The final model generated through this process is presented in Figure 3 of the main manuscript, with expanded details for the stages of Prediction (1. 8. 1), Experiment (1. 8. 2) and Refinement (1. 8. 3) presented below. We stress that we present only a single iteration of the model-driven research process to illustrate how the software technology features of MUFINS can facilitate this process, and that new insights into macrophage biology would require further experimental and modelling work.

### Prediction of NO production in human macrophages in silico

The model developed in Use Case 1 describes the generation of a dynamic model of the mammalian macrophage; more specifically, the regulation of nitric oxide production (NO), a major output of the whole-cell metabolic network. The production of NO is regulated by a large scale network comprising signalling and gene regulatory interactions. To test whether the generated model was capable of predicting the effects of regulatory network perturbations on NO production, we calculated the maximal NO production in the original, “untreated”, model and in models constrained to represent three experimental treatments i) LPS, ii) a MEK inhibitor, and iii) LPS plus a MEK inhibitor. Treatment with LPS is represented by setting the bounds of the “*lps_input*” flux to (0, 100), where 100 is an arbitrary value used across the qualitative regulatory network model to represent maximal availability. Since LPS is an activator, we allow the upper bound to be the maximal value, but change to the lower bound is not required – the linear programming solver in MUFINS will determine the maximal value within these constraints needed to achieve maximal NO production rate. The MEK inhibitor treatment is modelled by setting bounds of the “*mek_inhibitor*” flux to (100, 100). Here, we force the flux rate to 100 by setting both maximum and minimum values to 100. This is to prevent the linear programming solver from considering a solution where the inhibitor flux is inactive. Moreover, by setting the value to the maximum value used within the qualitative regulatory network model, we exclude scenarios where NO is produced at the expense of the essential metabolic demands of the cell: the ability to account for global metabolic requirements and limitations is a major advantage of using genome scale metabolic networks, instead of smaller, pathway models. To achieve this, the growth rate (Reaction “*R_biomass*”) is constrained to 0.0281, a literature value reported for RAW264.7 macrophages producing NO, and used by the authors of the metabolic part of this model (18). The resulting model predictions are shown in Figure S5:

### Figure S5: Initial Model Predictions

Nitric oxide production was predicted for untreated RAW264.7 macrophages (untreated), plus cell cultures treated with a MEK inhibitor (MEKi), LPS (LPS), or both LPS and MEK inhibitor (LPS + MEKi).

As demonstrated in Figure S5, the model predicts that NO synthesis requires stimulation of cells with LPS treatment, and that co-treatment with a MEK inhibitor does not alter the rate of NO production.

### Experimental determination of NO production in RAW264.7 murine macrophages in vitro

To test the model predictions, we measured nitrate production in RAW264.7 macrophages *in vitro*. Nitrate is formed through the non-enzymatic conversion of nitric oxide produced by RAW264.7 cells; as there is no nitrate consumption in the medium, nitrate concentrations are proportional to the cellular nitric oxide production flux. As detailed in section 1.7, RAW264.7 cells were exposed for 24 hours to i) medium alone, ii) LPS, iii) the MEK inhibitor PD98059, or iv) LPS and PD98059 combined (Section 1.7). Nitrate levels Experimental results are shown on Figure S6:

### Figure S6: Nitrate production of RAW264.7 macrophages

RAW464.7 macrophages were culture in DMEM and exposed to 10ng/ml LPS, 4µl/ml PD98059, or a combination of both chemicals for 24 hours. Nitrate levels in the medium were determined and compared to medium conditioned by untreated cells.

The experimental results demonstrate that NO production in untreated RAW264.7 cells is below the detection limit of the assay, equivalent to 2.5 µM nitrate in the medium. Exposure to the MEK inhibitor PD98059 has no significant impact on nitrate levels in the medium, but exposure to LPS for 24 hours leads to a significant increase to approximately 30 µM. Since the non-enzymatic conversion of the NO produced by RAW264.7 cells is the only source of nitrate in the medium, this demonstrates that LPS treatment is required for NO production, consistent with the published literature. Co-treatment of RAW264.7 cells with both LPS and PD98059 resulted also resulted in detectable nitrate levels in the medium, but these were significantly lower than observed with LPS alone. This data is consistent with MEK inhibition resulting in a perturbation, but not ablation, of the LPS-induced NO production.

### Refinement of the in silico model based upon experimental observations.

Comparison of the model predictions with the observations from the experimental data allows identification of areas for refinement within the model. The initial model is able to correctly predict that untreated macrophages do not produce significant levels of NO, and that treatment with a MEK inhibitor does not affect this. In addition, the model correctly predicts the increase in NO production associated with LPS treatment. However, the model fails to predict the observed decrease in NO production when cells are exposed to both LPS and The MEK inhibitor PD98059. To address this discrepancy, we used the interactive visualisation available in JyMet to identify additional constraints to the model that would explain the decrease, but not complete abolishment, of NO production upon co-treatment with LPS and a MEK inhibitor.

As shown in Figure S4, NO is generated in the model through the action of NO synthase, a gene encoded by the iNOS gene, which is represented by the iNOS molecular entity in the model represents the iNOS gene. iNOS is directly regulated by three reactions in the model, all of which activate gene expression: ERK1/2->iNOS, HIF1->iNOS, JNK->iNOS: these reactions represent the action of the transcription factors ERK1/2, HIF1 and JNK. MEK1 acts as an activator of ERK1/2, being represented in the model as the reaction MEK1->ERK1/2. As such, MEK1 is an indirect positive regulator of iNOS activity, and ultimately NO production. Inhibition of MEK1 is represented by the molecular entity MEKi, which acts on the reaction MEK1->ERK1/2, thus limiting availability of active ERK1/2. MEKi does not affect in any way the availability of HIF1 and JNK; this was confirmed by running simulations where HIF1 and JUN were set as objective functions. The experimental observation that MEKi has a significant impact on NO production can only be produced if HIF1 and JUN activate iNOS to lesser extent than ERK1/2. Only in this case does decreased availability of ERK1/2 impact on the level of iNOS activation and NO production flux. When either HIF1 or JUN can fully activate iNOS, loss of ERK1/2 has no impact on NO production.

The simplest possible explanation for the observed results is that the impact of HIF1 and JUN on iNOS transcriptional activity is less than the impact of ERK1/2. We refined the model through introduction of constraints limiting the impact of HIF and JNK: the reactions HIF1->iNOS and JNK->iNOS were constrained to (0,0.005) while ERK1/2->iNOS was left as (0,100). However, these flux limitations, or even complete inactivation of both reactions, still did not result in a lower NO flux in the presence of MEKi.

Further examination of the network connectivity revealed that ERK1/2 can be phosphorylated not only by MEK1 but also by PKC and PKC. Therefore, any impact of MEKi on ERK1/2 phosphorylation will be negated if ERK1/2 is efficiently phosphorylated through these routes. As the experimental data demonstrates that MEK inhibition reduces NO production, but does not ablate it, the most plausible explanation is that MEK1 is a more potent ERK1/2 kinase than PKC. To examine this, we further refined the model such that PKCA/B->ERK1/2 was constrained to (0,0.005). Simulation of the maximal NO production under these conditions are shown below (Figure S7) and in Figure 3D of the main manuscript.

### Figure S7: Refined model predictions

Nitric oxide production was predicted for untreated RAW264.7 macrophages (untreated), plus cell cultures treated with a MEK inhibitor (MEKi), LPS (LPS), or both LPS and MEK inhibitor (LPS + MEKi). The bounds of reactions JNK-> iNOS, HIF1->iNOS and PKC A/B -> ERK1/2 were set to (0, 0.005) according to our mechanistic hypothesis that: i) ERK1/2 is a more potent transcriptional activator of the iNOS gene than JNK and HIF1, and ii) MEK1 is a more potent ERK1/2 kinase than PKC.

As demonstrated in Figure S7, incorporation of three constraints into the regulatory network allows the theoretical predictions to qualitatively reproduce the experimental data: LPS activation is required for NO production and MEK inhibition decreases, but does not fully abolish, NO production. The decrease in NO production flux relative to LPS treatment alone, can be adjusted by altering the upper bound for the reactions: JNK-> iNOS, HIF1->iNOS and PKC A/B -> ERK1/2. The assigned upper bound (0.005), closely reproduces the experimental NO production rate, although a formal quantitative fitting was not undertaken. Thus, through an iterative cycle of prediction, experimentation and refinement we have developed two mechanistic hypotheses for future testing, namely: **i) ERK1/2 is a more potent transcriptional activator of the *iNOS* gene than JNK and HIF1, and ii) MEK1 is a more potent ERK1/2 kinase than PKC**.

To summarize, we demonstrate a single iteration of simulation, experimental validation and subsequent model refinement based on discrepancies between the initial predictions and experiments. This results in both an improved concordance between the model prediction and observed biology, and the generation of mechanistic hypotheses for future testing. We stress that this single iteration of the systems biology workflow is not sufficient to fully validate the genome scale model of metabolic reprogramming in mammalian macrophage presented here. Many more iterations would be required with experiments examining a large number of outputs within both the signalling and metabolic networks. Rather, the purpose of this Use Case is to illustrate the software features available within MUFINS, and how they may be implemented in this prediction-experimentation-refinement cycle. We show that MUFINS is currently the only tool that can be used to perform the computational section of the systems biology workflow using a constraint based model that integrates a signalling network with inhibitory interactions and a genome scale metabolic model. Moreover, the JyMet GUI makes this sophisticated computational approach accessible to experimental scientists. We believe that enabling experimental biologists to perform iterations of hypothesis generation, validation and refinement is necessary not only for future completion of this model of metabolic reprogramming in the mammalian macrophage, but also for the creation of mechanistic models describing molecular biology of human tissues at the genome scale. Such mechanistic rather than statistical models of the genotype-phenotype relationship in human tissues will allow us to fully capitalize on current molecular biology knowledge and offer unprecedented predictive power in model-based drug development and personalised medicine.

# Use case 2: Kinetic model of cortisol signalling integrated with dFBA simulation of human GSMN

## General Introduction

An important challenge within the modelling community is the integration of existing models to produce larger-scale reconstructions that are able to simulate larger areas of biology. The MUFINS framework is ideally suited to this as it allows the integration of models from a diverse range of frameworks, allowing the exploitation of the legacy of models available in repositories such as BioModels. To demonstrate this, in this use case we merge an existing model of nuclear receptor signalling, with the human Recon2 GSMN, plus a dynamic FBA constraint to represent the requirement of liver cells to maintain glucose and lactate levels within the blood. An overview of the model is presented in figure S8, below, with a full description provided within the subsequent methods section.


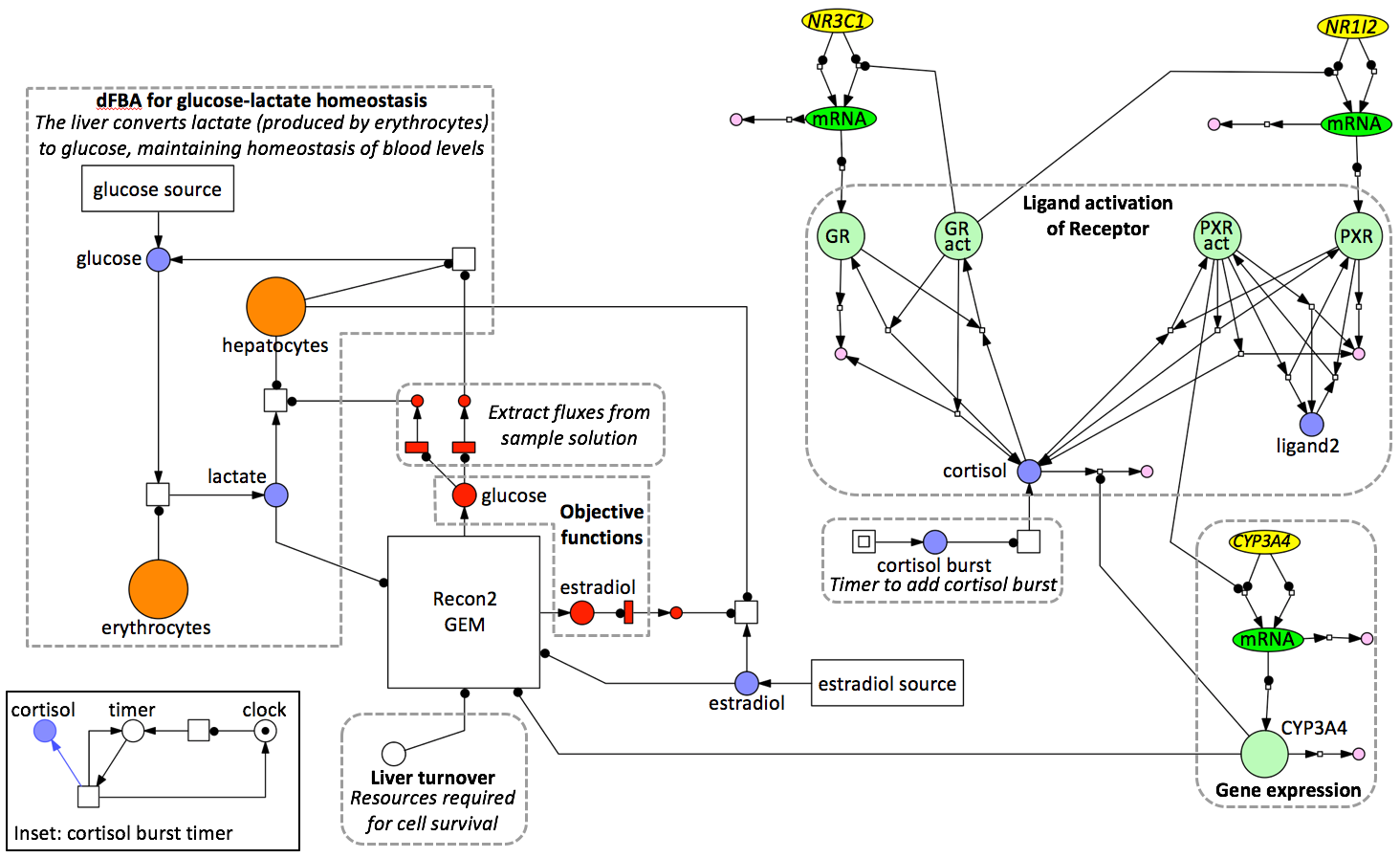


### Figure S8: Overview of multi-formalism model of cortisol signalling in human liver.

The model is constructed of several functional units, indicated by the grey-hatched boxes. *Gene expression* is represented as both transcription and translation reactions, with associated degradation of the mRNA and protein products. Read arcs (line with filled circle) are used as both transcription and translation do not consume their substrates (i.e. gene and mRNA). *Ligand activation of receptors* represents the interaction of cortisol with the glucocorticoid receptor (GR) and pregnane X-receptor (PXR). Here, directed arcs (line with arrow head) are used to represent movement of species during reactions. In addition, the presence of a second ligand for PXR (ligand2) is simulated, allowing the examination of drug-drug interactions. Finally, a cortisol infusion can be simulated through a timer; for clarity this timer is hidden within a coarse transition (double squared box), with the timer shown as an inset to the main diagram. *Liver turnover* is used to set a constraint on the biomass reaction, representing the minimum level of resources (e.g. amino acids, ATP, nucleotides etc.) required for the hepatocyte to be able to replenish itself. *Objective functions* set the reactions or metabolites that will be maximised during FBA. In this case, the external metabolites for glucose and estradiol are used. From each FBA solution we *extract fluxes* that can be used to monitor the behaviour of the system. Finally, a *dFBA for glucose-lactate homeostasis* is included. This adds an additional constraint whereby the GEM must maintain glucose and lactate levels in the blood at 4mM and 1.5mM, respectively. Transitions denoted F1-F4 are specifically mentioned in the methods section.

## Methods

Complete preparation of the model described in figure S4 is detailed below. In addition, the final model is available within the MUFINS software, within: MUFINS_ExamplesNR_Recon2.

### Kinetic model of cortisol signaling

The development and validation of a model of the nuclear receptor-mediated cortisol signaling network has been described in detail in our previous work (8, 9). Briefly, the model simulates concentration changes of 42 molecular species describing the interactions between cortisol and two of its cellular receptors, the glucocorticoid (GR) and pregnane X (PXR) receptors. Activation of GR by cortisol results in a negative feedback on GR gene expression, and a positive feedforward on both PXR and tyrosine aminotransferease (TAT) gene expression. Activation of PXR by cortisol results in positive regulation of CYP3A4 gene expression, leading to enhanced degradation of cortisol by this enzyme. Transcription, translation, degradation, cortisol-protein and protein-DNA interactions are modelled with Ordinary Differential Equations (ODEs), using either mass action kinetics or complex reaction mechanisms (e.g. Michaelis-Menten), as appropriate. Justification for quantitative parameters of the equations and model validation are provided in our previous paper (8).

Here, we use final version of the model deposited in the BioModels database (10) under accession BIOMD0000000576. The SBML file of the model as well as tables with equations and parameters are available from BioModels entry BIOMD0000000576.

### Preparation of continuous Petri Net representation of the kinetic model of cortisol signalling

The kinetic model of cortisol signalling was downloaded as an SBML file from BioModels, and imported into SNOOPY as a continuous Petri net using (File🡺Import). The imported model was then manually curated to (i) remove species that are not required for the current simulation, and (ii) alter visualization to match the SBGN format (Figure S8). Neither step is an absolute requirement for simulation, but both aid in both understanding simulation output and presentation of the model in a manner visually understandable to non-specialists. As the original kinetic model is composed of species and reactions and ODEs were defined as sums of reaction rates, import into the Petri Net representation did not require any changes to the model equations or parameters. Molecular species are represented as places and reactions as transitions, with propensity functions defined by the rate equations. Once manual curation of the model is complete, it can be saved as a SNOOPY file using (File Save)

NOTE: Depending on the version of SNOOPY used, files will be tagged *.spept (older versions) or *.xpn (v1.2 and above). MUFINS is able to work with both file formats.

### Preparation of Recon2 human genome scale metabolic model

To represent human metabolism we have used Recon2 – the community-based reconstruction of generic human metabolism (11). The SBML file from the supplementary material of the original publication (recon2_model.xml) was downloaded and imported into JyMet using the ‘File-Import SBML’ command. We note, that Recon2 is also available from BioModels (MODEL1109130000). In its native state, Recon2 has all reaction bounds set to full open (1000 or -1000 for forward and reverse direction, respectively). To constrain the solution space for flux balance analysis, and hence improve the robustness of biological predictions, it is important to add additional constraints to the reconstruction. This was achieved by: first, constraining exchange reactions to model the physiological conditions of a liver cell; second, further constraining the PIPES exchange reactions to mimic experimental data on metabolite exchange.

To constrain the exchange reactions to model the physiological conditions of a liver cell, we used the Physiological Import, Physiological Export Set (PIPES) from the HepatoNet1 human hepatocyte GSMN model. As HepatoNet1 (12) is integrated into Recon2 with reaction names maintained, identification of relevant reactions was straightforward. All exchange reactions were identified using the search function in JyMet (Edit🡺Search) using the string ‘R_EX_’, which is the identifier for exchange set reactions. Any Recon2 exchange reactions not listed in the PIPES were set as closed (i.e. set to 0, 0). All Recon2 reactions listed in the PIPES were set as open using the following bounds: (-1000,0) for import reactions, (0,1000) for export reactions and (-1000, 1000) for reversible reactions.

To further constrain the PIPES exchange reactions, increasing the quantitative accuracy of the model, we used experimental data to further constrain the exchange set bounds for reactions within the PIPES. Jain et al (13) measured consumption and release of 219 metabolites across the NCI-60 cancer cell lines, which we refer to as the CORE flux set. We assumed that none of the transport reactions under physiological conditions carries flux larger than the largest flux observed in cancer cell lines. This assumption is justified by the fact that cancer cell lines *in vitro* divide faster than healthy tissue and thus have larger nutritional requirements. Hence, the *in vivo* healthy tissue nutrient uptake/release is very unlikely to be larger than the largest (absolute value) flux observed across the cancer cell lines. Thus, the CORE flux set provide an excellent resource of experimentally determined bounds. The CORE flux values for all 219 metabolites across the 60 cell lines were imported into Excel, and the maximal flux value for each metabolite determined. This value was then used to replace the relevant exchange bound within Recon2. For example, the maximal consumption flux for glucose across the NCI60 cell lines is 1.723855 mmol/ gDW /h. Therefore, the glucose exchange reaction (R_EX_glc_LPAREN_e_RPAREN_) has bounds set to (-1.723855, 1000), denoting a constraint on uptake, but unlimited efflux of glucose. Where a PIPES exchange reaction was not examined in Jain et al., flux values were left fully open (i.e. -1000,0 or 0,1000 or -1000,1000, as required).

The final GSMN model is available in the MUFINS1.0_Examples/NR_Recon2 directory of software distribution as MUFINS reaction table file recon2_xt.PIPES.CORE.v1.sfba.

We note that while exchange reactions were constrained to reflect knowledge nutritional environment of liver cell, we did not use a tissue specific liver model. We chose to use Recon2 rather than the liver-specific HepatoNet1 because we are convinced that community based development of further versions of Recon GSMN is the future direction in modelling human metabolism. In addition, Recon2 is substantially larger than HepatoNet1, incorporating 7440 reactions compared to 2539, and thus has the potential to reveal more biological insights through greater interconnectivity. Generation of tissue- or context-specific GSMNs, which entails setting bounds for all reactions present within the GSMN using ~omics data allows further constraint of the solution space for FBA. This approach is fully discussed in Use Case 3: Analysis of a clinical transcriptome study to understand in vivo tumour metabolism.

### Integration of cortisol signaling model with the Recon2 human GSMN

Once both the signalling network and GSMN have been prepared, they can be integrated in SNOOPY for subsequent QSSPN simulation.

Within SNOOPY, Recon2 GSMN is represented as a transition named ‘Recon_2’. Within the comments section of the transition, two command lines are added: first ‘MODEL Recon2_PIPES’ set the transition type as a link to a model; second, ‘EXT_TAG _xt’ specifies the external tag used within the GSMN.


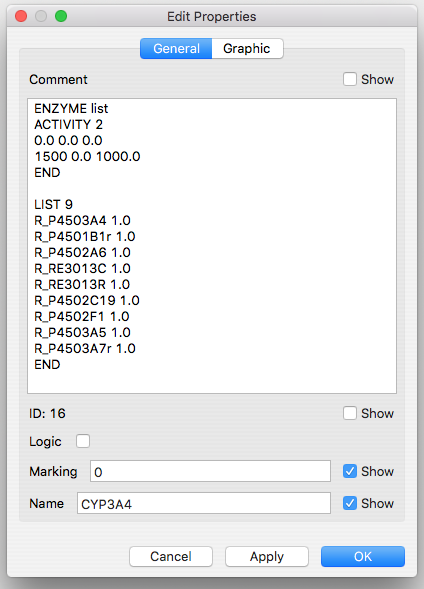
Connection between the continuous PN representing the kinetic model of cortisol signalling and the GSMN occurs through the QSSPN constraint place representing the CYP3A4 protein, as denoted by the read arc between the CYP3A4 protein place and the Recon2 transition. To provide a conversion between the expression level of CYP3A4 protein in the continuous Petri net and bounds for all reactions catalysed by CYP3A4 within Recon2 a lookup table is added to the comment section of the CYP3A4 protein place (Figure S8). As shown in the figure, this lookup table is composed of two parts: a list of all reactions that the protein catalyses within Recon2, and a set of activity values. Where only a single reaction is mapped to a protein, then this can be entered directly next to ‘ENZYME’, but where multiple reactions are effected a list is used (as shown here). The number next to each reaction (e.g. 1.0) sets the relative impact of the activity list on each reaction, allowing differential control of reactions by a protein, if desired. The activity list sets the bounds for these reactions at any given token status for the protein place. In this case, the flux bounds of all nine reactions are closed (0, 0) for CYP3A4 concentrations smaller than 1500 nM and fully open (0, 1000) at higher concentration. This threshold is sufficient parameterisation for the conclusions presented in this use case, but we note that lookup tables with more levels could be constructed to interpolate rate equation of CYP3A4 enzyme and quantitative parameters.

### Figure S9: Mapping of protein levels to reaction activity status for QSSPN simulation

To link token status within a Petri net representation of a gene or signalling regulatory network with reaction bounds within a GSMN, comments are added to the QSSPN constraint place (e.g. place representing a metabolic enzyme). The comments specify, (i) which reactions are linked to the constraint place, and (ii) the activity of these reactions for any given token status of the constraint place.

### Simulation of physiological compartment dynamics for glucose, lactose and estriadol

Multi-formalism simulations, where GSMNs are integrated, with Physiologically Based Pharmacokinetic (PBPK) models is an important future direction in mechanistic modelling for pharmaceutical applications (17). PBPK models use ODEs to describe changes in substance concentrations in physiological compartments such as blood, liver, brain *etc.*, and take into account factors including drug absorption, distribution, metabolism and excretion. The integration of GSMN and PBPK models allows the mechanistic connection between physiological-level effects and genotype (14). To demonstrate how MUFINS can be used to integrate PBPK constraints with a metabolic reconstruction we have constructed a dynamic FBA model that quantitatively constrains the Recon2 GSMN through an ODE model of lactate and glucose concentration in blood. A major physiological function of liver is to achieve homeostasis for a range of chemicals within the body, including glucose and lactate. Erythrocytes (red blood cells; RBCs) produce lactate during normal cellular respiration, which is then excreted into the surrounding blood. As elevated lactate levels may be deleterious, lactate is taken up by the liver and converted where it can be converted to glucose. Excretion of glucose by the liver is important to maintain blood glucose concentrations for use throughout the body.

We reconstructed lactate production by RBCs and subsequent regeneration of glucose by the liver using QSSPN. The graphical, PN representation of the model is shown in Figure S5: glucose and lactate in blood were modelled as PN places, with continuous marking representing concentration in mM. Lactate is formed from glucose in the blood via transition F2: to scale lactate production to a physiological level, a additional PN place was connected to F2 via a read arc, representing the total number of RBCs in the blood (Figure S8). The produced lactate concentration in the blood was modelled as QSSPN constraint place, representing the availability of lactate for the lactate exchange reaction in the Recon2 GSMN. As shown in figure S5, if lactate concentration was below 0.001 mM, the exchange reaction was constrained to (0, 1000), preventing uptake of lactate. Otherwise, the lactate exchange reaction flux was set to (-0.0565, 1000) representing saturating lactate uptake rate of 0.0565 mmol/gDW/h (see below for parameter determination).

The metabolic utilisation of lactate to form glucose in the liver has been represented by setting the FBA objective function to maximisation of external glucose. This objective function has been represented by the QSSPN objective place glucose (Figure S8), with commands placed within the comments section to specify the metabolite to optimise (external glucose), plus an activity table to prevent the use of this objective if it is infeasible (Figure S10). To extract flux values from the FBA solution we use QSSPN flux transitions. These are connected to the objective function via a read arc, and access values of the FBA solution for the specified flux fluxes. A directed arc transfers this flux value to a place, where it can be read (Figure S8). The token values for these flux places are then used as inputs for rate equations within transitions F1 and F3, representing glucose production and lactate consumption by the hepatocyte, respectively. To scale the simulated flux values to a physiological level, F1 and F3 reaction fluxes are modified by a PN place representing the total number hepatocytes, connected via a read arc (Figure S8). These involved in production of lactate, with continuous marking representing cell density in gram dry weight per litre (gDW/L). Finally, to represent the net contribution of the rest of the body to glucose blood levels, a glucose source transition is added. This transition adds glucose to the blood at the rate required to achieve the known steady-state glucose blood concentration (-2.76 mmol/L/min).


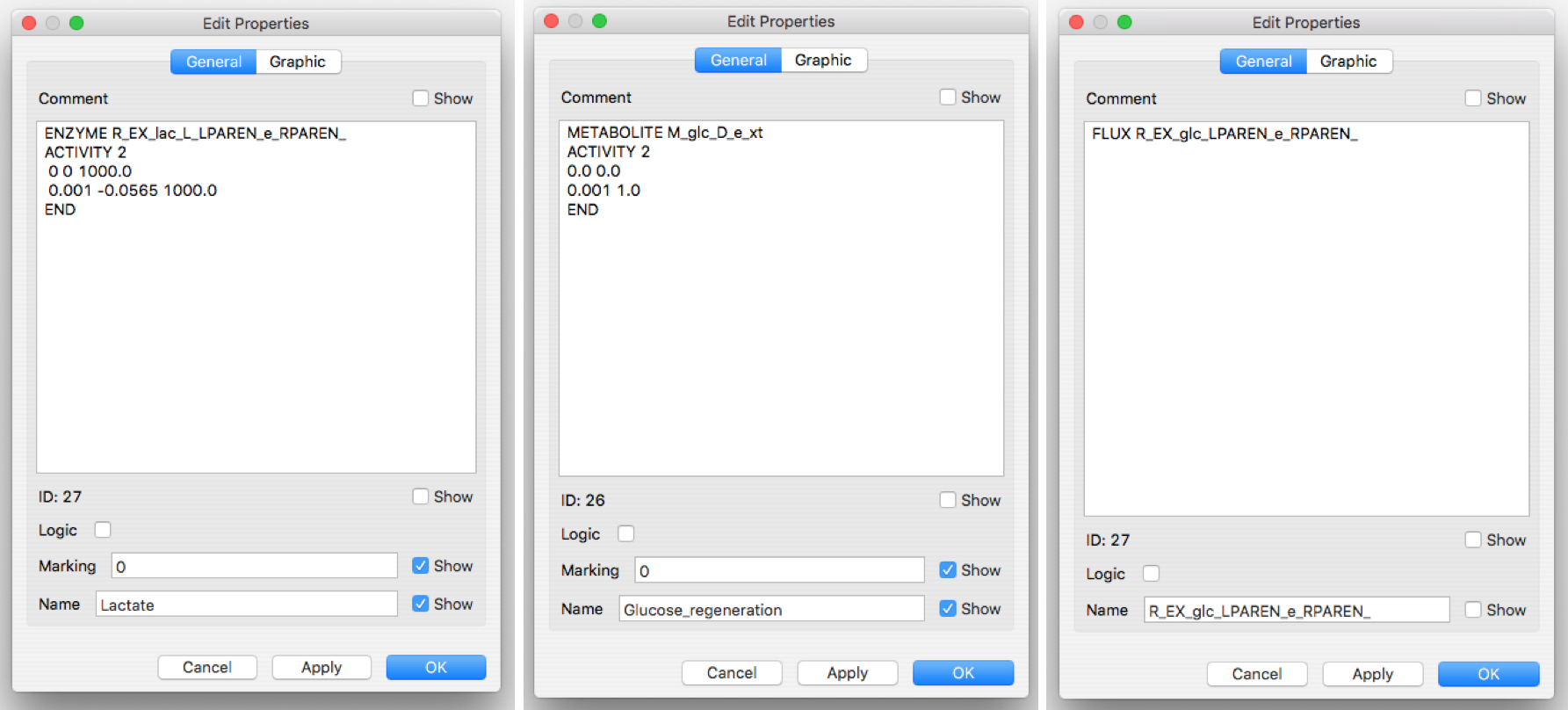


### Figure S10: Generation of physiological compartment dynamics for QSSPN simulation

Properties are shown for: QSSPN constraint place for lactate consumption (right), QSSPN objective place to set the objective function for FBA as maximisation of production of external glucose (middle), and QSSPN flux transition to extract flux value for glucose exchange from the FBA solution in the pre-place.

### Simulation of physiological compartment levels for estradiol

Flux Variability Analysis was used to examine the influence of reactions catalysed by CYP3A4 on the exchange reactions of Recon2. Through this method, estradiol, a physiologically important substance, was identified as having its exchange affected. To allow simulation of the impact of the signalling network on intracellular concentrations of estradiol, we added a second QSSPN objective function that maximises production of estrogen within the cytosol (M_C05301_c). The flux rate of estradiol external production is extracted from the FBA solution, and then scaled to whole organ level through transition F4, as described for glucose and lactate.

### Use of biomass constraint to represent cell turnover with an organ

For prokaryotes and eukaryote tumour cells, a major biological objective is cell division. As such, increase in biomass is commonly used as the objective function for FBA. This objective is usually represented as a composite biomass equation, which encompasses production of all the components required to replicate cells (e.g. nucleotides, amino acids, ATP *etc.*) in their required ratios. The majority of mammalian organs do not actively divide, and hence an objective of biomass is inappropriate. However, organs such as the liver, while not actively growing, must replace cells continuously to maintain their mass. We represent this requirement as a QSSPN constraint place (Liver turnover), which sets a minimal value of biomass flux that must be achieved.

### Parameter values used within the model

Parameters for the cortisol signaling network are those used in the original paper (8), and are not described herein. All other quantitative parameters of the model were determined as follows: The RBC cell density was set to 400 gDW/L based on 2 kg of RBCs in 5 L of blood (John Baynes, Marek H. Dominiczak, Medical Biochemistry, Elsevier Health Sciences, 24 Jan 2014). As the glucose and lactate concentrations were calculated for volume of blood compartment, we used the blood volume to calculate the hepatocyte cell density. The density of hepatocytes metabolising lactate and glucose from litre of blood was set to 19.7 gDW/L, based upon measurements of human hepatocellularity (1.96546 10^11^ hepatocytes in 1414 g of liver; (15)) and a cell density of 500 pg/cell, as used in Recon2 (11). The rate of glucose conversion to lactate by RBC has been experimentally determined as 0.1 mmol of glucose being converted to 0.2 mmol of lactate per day (John Baynes, Marek H. Dominiczak, Medical Biochemistry, Elsevier Health Sciences, 24 Jan 2014). Physiological levels of lactate and glucose are 1.5 mM and 4.5 mM respectively, and were obtained from MOPED (16). The maximal value of glucose production, calculated using Recon2 constrained with CORE fluxes is 8.5 mmol/gDW/h. Using these data, we were able to determine remaining parameters of the model (Table 1 below). Note that the rate of transition representing net glucose exchange with other physiological consumption is negative, consistent with glucose consumption rather than production by other tissues.

### Table S1. New parameter values used in Use case 2.

| **Parameter** | **Value** | **Comment** |
| --- | --- | --- |
| RBC cell density | 400 gDW/L | Dry weight per 5 L of blood |
| Hepatocyte cell density | 19.7 gDW/L | Dry weight per 5 L of blood |
| Transition F1 rate constant | 0.016666667 h/min | Time unit conversion |
| Transition F2 rate constant | 1.54 10^-5^ L/gDW/min | Lactate production per litre of blood per minute. Based on textbook rate of 0.2 mmol of lactate per day (John Baynes, Marek H. Dominiczak, Medical Biochemistry, Elsevier Health Sciences, 24 Jan 2014). |
| Maximal lactate uptake rate (constraint place “Lactate”). | -0.0565 mmol/gDW/h | Calculated based on physiological steady state of lactate and maximal glucose regeneration calculated by Recon2. |
| Net glucose exchange | -2.57 mmol/L/min | The rate required to balance glucose at physiological steady state. |

### Preparation of control file for QSSPN

Before a QSSPN simulation can be undertaken, a control file must be created as a text file, setting the rules for the simulation. A control file contains the following components

MODEL: this defines the model to be simulated. As this is placed in the same folder as the control file, it’s location is designated by ./name.sfba

NUMBER_OF_SAMPLES: Sets the number of times that a simulation is run (Default=1)

SEED: Sets the seed for the random number generator used during stochastic transitions

TIME_MAX: simulation time

MAXIMAL_TIMESTEP: Sets the timestep for the simulation

MAX_CHANGE: Allows an adaptive timestep to be introduced if necessary. A value or 0.01 indicating a 1% change in token status of a palace

OUTPUT: Specifies the file that stimulation results are written to. Usually an Excel file within the current directory (e.g. ./name.xls)

LOG: Specifies the file for any log entries to be written to. Usually a txt file within the current directory (e.g. ./name.txt)

MONITOR: Sets the number of interations between writing of data to the output file. This parameter can be altered to achieve sufficient granularity in the simulation output

INITIAL STATE: Sets the token state for places within the Petri net

PROPENSITY_FUNCTION: Allows algebraic formulae to be used to define the transition propensity. Must finish with END

RESET_FUNCTION: Sets the value of the post-place based upon the pre-place (e.g. to be exactly the pre-place value, or 1/20 *etc.*). Must finish with END.

PETRI_NET_MONITORS: sets which places have their token status written to the output file

/* … */: Used to enclose comments

NOTE: values in the control file will overwrite any values entered into places or transitions within the SNOOPY file. For best practice, we recommend that simple (i.e. mass action kinetics) rates are placed within the SNOOPY file, while complex (e.g. Michaelis-Menten kinetics) rates are placed within the control file. All initial values that are not default (i.e. 1 or 0) are best placed within the control file.

An example control file is shown below (Figure S11). This is a shortened version of the control file used in Use Case 2.

###
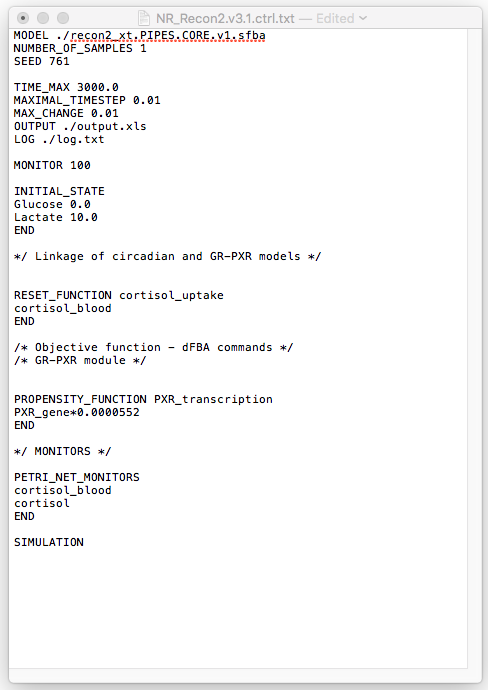
Figure S11: Generation of the control file for QSSPN simulation

### Simulation of complete model

Once the model and control file have been created, the model can be simulated. This can be achieved either through the command line, or using the JyMet interface. In both cases, the algorithm files must be present within a sub-folder (bin) of the folder that contains the model file to be simulated. Hence, for a simulation, the top folder must contain the model file (*.spept or *.xpn SNOOPY file), the control file (*.ctrl.txt), the GSMN (*.sfba), and the folder containing the simulation algorithms (bin). Examples of this layout can be seen in the MUFINS_Examples folders.

#### Running simulations in the command line

Under a Mac OS, start terminal, and navigate to the folder containing the simulation files and enter the following commands

*./bin/spept2qsspn name.spept x > name.qsspn*

this converts the named *.spept or *.xpn file to a qsspn input format. x specifies the largest possible number used in the simulation. For qualitative simulation, this can usually be set as 2, but for quantitative simulation we recommend 1e9 (a value unlikely to be exceeded by any species within the network).

*./bin/qsspn name.qsspn name.ctrl.txt*

this executes the simulation. After a brief pause, simulation progress will be seen in the terminal window, with each number representing the simulation time for each MONITOR step.

For Windows OS, the above can be used in the Windows Command Line, using Windows syntax. A python interpreter is required to run spept2qsspn. In general, we recommend running simulations using JyMet under Windows OS

For Linux OS, user should execute two command lines using either binaries available in bin or go to MUFINS**_Source/QSSPN/ and run compile.sh, install.sh to create new binary in MUFINS**_Source/QSSPN/bin . Both qsspn and spept2qsspn do not require any libraries or environmental variables and can be moved to any convenient location on the system, such as /usr/local/bin.

Once the simulation has been completed, the output file can be opened in Excel to view the simulation results.

#### 2.10.2 Running simulation in JyMet

The implementation of a JyMet interface to QSSPN simulations is major new feature of MUFINS. Upon starting JyMet from, three files must be imported. The first is the GSMN model, designated as name.sfba, and can be openend via (File🡺Open Model). Second, the SNOOPY file must be imported (QSSPN🡺Import SPEPT). During this process, the program prompts for to “Set default maximal number of tokens”. As commented above, this is best left at 2 for qualitative simulations and 1e9 for quantitative simulations (a value unlikely to be exceeded by any species within the network). Finally, the control file must be loaded (QSSPN🡺Load control).

Once all the necessary files have been imported, simulation can be undertaken. To start the simulation, select the command (QSSPN🡺Run). Once the simulation has finished, the “Output” tab will appear, containing data from all Petri net places specified in the control file. These can be visualized by selected them and then using (QSSPN🡺Plot Trajectory). A results file is automatically written in the simulation folder, with the name specified under ‘OUTPUT’ in the control file. This can be opened in spreadsheet programs such as Excel for further examination.

### Treatment of primary human hepatocytes with cortisol.

Primary human hepatocytes were a kind gift of GlaxoSmithKline. Cells were seeded at 2.4 x 10^5^ cells/cm^2^ in 25 cm^2^ flasks using Lebovitz L-15 medium with L-glutamine and amino acids, plus 10% foetal bovine serum, 100x non-essential amino acids, and allowed to attach overnight. Cells were exposed to vehicle (0.1% DMSO) or 1μM cortisol (Sigma Aldrich, Poole, UK) for 24 hours in serum-free medium, and then total RNA and protein isolated from quadruplicate samples. Total RNA was isolated using the RNeasy Mini kit (Qiagen, Crawley, UK) and quantified using a Nanodrop Agilent 2100 Bioanalyser, while total protein was extracted in RIPA buffer (1xPBS, 1% Nonidet P40, 0.5% sodium deoxycholate, 0.1 % SDS, protease inhibitor cocktail).

For measurement of estradiol clearance, estradiol was prepared at 1 mg/ml with 0.1 % DMSO (v/v) in optima grade methanol, and then further diluted to 1 µg/ml oestradiol with 2 mM sodium glycocholate in optima grade methanol to enhance solubility. The sample was dried with nitrogen and re-suspended in HBSS buffer supplemented with 10 mM 2-(N-morpholino) ethanesulfonic acid. Cells were exposed to vehicle or cortisol as previously described. At the end of the 24 hour incubation, medium was refreshed with the addition of 1000nM estradiol. Samples of conditioned medium were taken at 0, 15, 30, 45, 60, 90, 120, 150, 180, 210, 240 minutes and then hourly until 8 hours. Conditioned medium samples were prepared for UPLC-MS analysis as follows: conditioned medium was vortexed for 30 seconds, left for 30 minutes at room temperature, and vortexed again. Acetonitrile (1:1 v/v) was added, the sample vortexed for 30 seconds and left at room temperature for 30 minutes. Samples were then vortexed one final time, and centrifuged at 10,000 xg for 15 minutes.

- 1. Determination of CYP3A4 expression and activity levels

For transcript measurement, primers and TAMRA/FAM dual labelled probe specific for CYP3A4 and 18S were designed using the Primer Express software (Applied Biosystems, Warrington, UK) and were purchased from Eurofins Wolverhampton, UK). Total RNA was treated with RNase-free DNase (Promega, Southampton, UK) to remove genomic contamination. Reverse transcription was primed with random hexamers and carried out by Superscript II (Invitrogen) as per the manufacturer’s instructions. To ensure that DNase treated samples were free from genomic contamination an RT- control (lacking enzyme) was carried out for every RNA sample. cDNA generated from 50 ng (PXR, GR, TAT and CYP3A4) or 50 pg (18S rRNA) of total RNA was amplified using TaqMan Universal PCR Master Mix with 400 nM primers and 200 nM fluorogenic probe in a total reaction volume of 25 µl. Quantitative polymerase chain reaction (Q-PCR) reactions were run on the ABI7000 SDS instrument and quantified out using the ABI proprietary software against a standard curve generated from human genomic DNA (Promega).

For protein measurement, total protein extracts (10 μg per lane) were resolved on 12 % SDS-polyacrylamide gels and then transferred electrophoretically to Hybond ECL nitrocellulose membranes (Amersham Biosciences, Little Chalfont, Bucks, UK). Membranes were blocked (1 hour) in 5 % fat free dried milk and then probed with primary antibodies against human CYP3A4 (SAB2105781, 1:250; Sigma-Aldrich) or α-actin (sc1616, 1:500; Autogen Bioclear), followed by anti-rabbit IgG (sc2030, 1:10000; Autogen Bioclear) or anti-goat IgG (sc 2020, 1:20000; Autogen Bioclear) respectively. Bound antibodies were visualised using enhanced chemiluminescence reagents according to the manufactures instructions (Amersham Biosciences).

CYP3A4 enzyme activity was determined using the P450-Glo activity system (Promega, Southampton, UK), as per the manufacturer’s instructions. Briefly, Luciferin-PPXE substrate was mixed with S9 protein extract from primary human hepatocytes, along with an NADPH regeneration system and detection reagent. Following 20mins incubation, luminescence was quantified using a Packard Lumicount.

### UPLC-MS detection of estradiol in cell lysates and conditioned medium

An Acquity UPLC system coupled to a Xevo triple quadropole mass spectrometer was used for LC-MS analysis. An Acquity UPLC BEH C8 1.7 µm, 2.1 x 100 mm column was used for chromatographic separation coupled to an Acquity UPLC vanguard column of the same chemistry. The method was based on the methodology of Guo et al. (17) with modifications. The separation of metabolites were carried out under reverse phase conditions using Fisher optima grade water as mobile phase A and optima grade methanol as mobile phase B. Capillary, cone and collision voltages were set to 1.6 KV, 30 V and 32 V, respectively. The parent ion for oestradiol was detected at 269.2 m/z, while daughter ions were detected at 145.8 m/z and 161.1 m/z.

# Use Case 3: Analysis of a clinical transcriptome study to understand in vivo tumour metabolism

## General Introduction

CBM has achieved unprecedented success in mechanistically modelling the relationship between genotype and metabolic phenotype at whole-genome scale. The GSMN of the organism of interest can be used as an off-the-shelf component of a multi-scale model, describing biosynthetic precursor and energy conversion engine of the cell. In Use Cases 1 and 2 we have shown how this can be done in MUFINS, both in the case of steady state and dynamic models. An important branch of CBM methodology (18) is dedicated to using ~omics data on metabolic gene activity to create tissue and/or condition specific GSMNs. We are convinced that constraining GSMNs by condition, tissue or patient specific ~omics data will be an important step in many mechanistic modelling projects. Therefore, we have not only made sure that MUFINS is equipped with state-of-the art CBM methods in this area, but have also developed Fast iMAT, a new variant of the iMAT congruency approach that is applicable to large ~omics sample numbers, where iMAT becomes impractical.

A preliminary version of the Fast iMAT approach, where gene expression data were discretised to two levels (absent transcript, present transcript) has been already used to analyse 2000 individual breast tumour transcriptomes from the Metabiric dataset (19). This has led to discovery of poor prognosis cluster with active serotonin production – an important biological insight (20).

Here, we present the mature version of Fast iMAT, where expression data can be discretised to three levels, including up-regulated expression. To illustrate this feature we have applied MUFINS to analyse a previously unexamined transcriptome datasets from Metabric (19). For 131 patients, two samples were collected: one from a breast tumour, and a second from a matched-normal region of breast tissue, leading to 262 transcriptome samples. This is ideal for analysis of how tumour differs from normal tissue, while our published analysis focussed on personalised differences between different tumours.

## Methods

Figure S12 shows our computational pipeline using the sfba engine of MUFINS to analyse 262 personalised, clinical transcriptome samples in the context of human Recon2 GSMN.

### Metabric datasource

As part of the Metabric (Molecular Taxonomy of Breast Cancer International Consortium) study, transcriptomic data from over 2000 breast tumours was generated, with full details of the patient population available in the accompanying publication (19). The data is available through the European Genome-phenome archive (<https://www.ebi.ac.uk/ega/home)>, and uses the Illumina HumanHT 12v3 beadchip microarray platform. In Use Case 3 we use dataset EGAD00010000212 containing tumour and matched healthy tissue transcriptomes for 131 patients. The human genome scale metabolic network (GSMN) Recon2 has been downloaded from its public repository (<http://humanmetabolism.org/>) (11).

### Transcriptomic data pre-processing

Raw transcriptomic data was first pre-processed to remove data from bad quality probes, normalize expression levels across bead arrays and summarize probeset data to derive expression levels for each target gene. All data manipulation and analysis were performed using scripts written in the R statistical programming language and the Bioconductor (21) and RStudio (22) interfaces.

The Bioconductor package *beadarray* was used to import raw images, generate quality assessment information, carry out image processing to adjust for spatial artefacts, and undertake background correction. Probes were then filtered by detection score, with a cut off p-value set as 0.05 (21, 23)


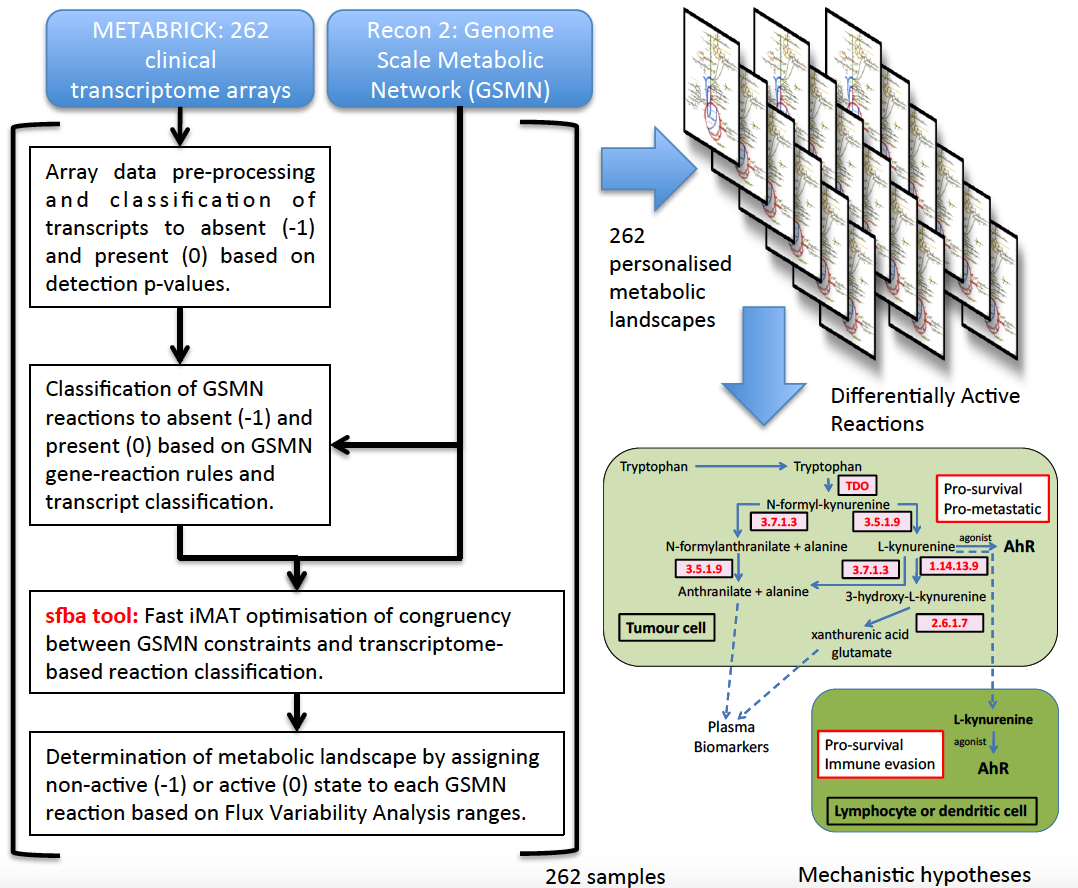


### Figure S12: Overview of fast iMAT analysis of metabolic reprogramming in cancer and healthy breast tissue

The METABRIC dataset contains transcriptome data for 131 individual tumours paired with healthy tissue samples from the same patient. The sfba tool has been incorporated into the computational pipeline shown above. The Fast iMAT algorithm allowed calculation of personalised metabolic landscapes for each of 262 transcriptome samples. From these metabolic landscapes, differentially activated reactions can be identified, leading to mechanistic hypotheses.

### Prediction of metabolic landscapes

Once presence or absence of transcripts was established the gene-reaction association rules of Recon2 model were used to find classify reactions as absent or present. In the case of multiple genes associated with the reaction, Boolean expressions of the Recon2 rules were used to determine whether reaction is operational, given presence/absence of the transcripts. Reactions, which were declared to be absent were assigned the state -1, remaining reactions were assigned the state of 0. Thus for each transcriptome, we obtained a vector assigning -1 or 0 state to each of 7440 reactions in Recon2. These vectors constituted input of Fast iMAT algorithm. For each of the input vectors, Fast iMAT algorithm identified flux distributions such that i) all stoichiometric and thermodynamic constraints were satisfied ii) maximal number of absent reactions (state -1) carried no flux. The Fasti MAT algorithm is formulated in Supplementary Methods section 3.

To directly compare Fast iMAT with original approach, we have analyzed transciptome data for the NCI-H23 cell line using both iMAT and Fast iMAT algorithms implemented in MUFINS. Both simulations were run with the sfba tool on a 2.2 GHz AMD Opteron^(tm)^ Processor 6174 on Linux. The Fast iMAT algorithm completed the task in 752 seconds, while iMAT calculations took 59725 seconds. Thus Fast iMAT is nearly 80 times faster (79.4). Supplementary Table 3 shows results of both simulations. Results of both methods are very similar; the prediction accuracy of iMAT result by Fast iMAT is 92%, which is statistically significant (p-value 4.3E-20).

### Identification of differentially expressed genes and functional classification

Standard eBayes analysis with BH multiple testing correction were used as implemented in limma. The functional descriptors overrepresented in the list of differentially expressed genes were identified using DAVID. Results available in Supplementary Table 2 show that features of metabolic reprogramming could not be identified with standard analysis of Differentially Expressed Genes.

## Results

Comparison of the predicted metabolic landscapes of paired normal and tumour tissue samples allows the identification of differentially activated reactions (DARs), which are either significantly more active in tumour tissue compared to normal tissue, or vice versa. A list of DARs is presented as supplementary table 2, as well as a comparison to standard statistical over-representation analysis of the transcriptome data to identify differentially expressed genes (DEGs). Figure S13 presents an example pathway predicted to have increased activity in tumour compared to normal tissue. Flux through this pathway, which produce kyneurenine, was predicted to be more active in 94/131 (72%; 95% CI 63-79%) tumour samples compared to their matched-normal tissue samples; the converse was predicted in only 7/131 (5%; 95% CI 2-11%) samples. This predicted increase in kyneurenine synthesis is consistent with the published literature: kyneurenine was recently established as the endogenous ligand for the Aryl hydrocarbon receptor (24), and implicated in pro-survival and immune evasion phenotypes (24, 25). It is important to note that this pathway was not identified through standard DEG analysis.

###
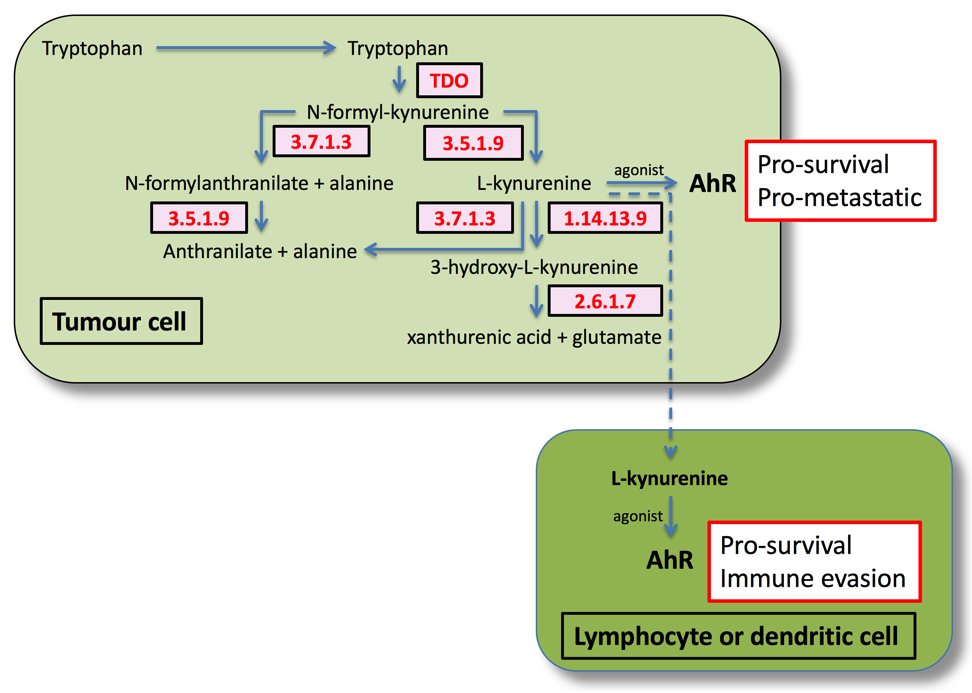
Figure S13: Breast tumours have significantly higher flux toward kyneurenine that matched normal breast tissue.

The METABRIC dataset contains transcriptome data for 131 individual tumours paired with healthy tissue samples from the same patient. Personalised metabolic landscapes for each of 262 transcriptome samples were generated using the Fast iMAT approach. The list of reactions whose states were significantly different between tumour and healthy tissues revealed altered tryptophan metabolism, predicting increased synthesis of L-kynurenine in tumours. This mechanistic hypothesis is in agreement with literature experimental data, where is is demonstrated to drive a pro-survival phenotype.

# TECHNICAL DESCRIPTION OF COMPUTATIONAL METHODS

# Linear Inhibitor and Activator Constraints

Vardi and colleagues (3) have published a linearized CBM approach for studying signaling networks. Here, we provide a general implementation of this approach, allowing for the first time the study of steady state systems composed of gene regulatory, signaling and metabolic networks. We introduce a definition of reaction formulas where substances can enter the formula not only as substrates but also as activators or inhibitors. The “&” operator preceding name of the substance introduces activator, which is required for the reaction. The “~” operator introduces inhibitor, which must not be produced in the system in order for the reaction to happen. Neither activators nor inhibitors are consumed during the reaction. An example formula is given below:

r: A + 2 B + & C + ~ 50 D = E + F

where, A and B are stoichiometric substrates of this reaction with stoichiometric coefficients of 1 and 2 respectively. C is activator and D is inhibitor. The coefficient in front of D is inhibition strength parameter. Extended reaction formulas are interpreted by the software in the following way.

For each activator *A* occurring in reaction *r* the following additional reaction *a* and reaction bounds are added to the system:


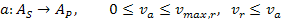


The activator A is represented by two “virtual” metabolites A_s_ and A_p_, which are the substrate and product of reaction *a*. A_p_ is an external sink metabolite for which flux balance is not required. Constraining flux through reaction *r* to be smaller than the flux through reaction *a* introduces demand for A_s_ in reaction r. If *v* for reaction *a* equals v_max_ for reaction *r*, this demand does not influence the quantitative value v_r_. However, if A_s_ is not available in the network v_r_ is constrained to 0. This makes A a cofactor that is required for reaction *r*, but is not consumed by reaction *r*.

Each inhibitor I in reaction *r* results in the creation of the following additional constraint in the Linear Problem representing the GSMN:


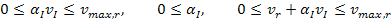


The coefficient α** **represents the inhibition strength, while v_I_ represents the producibility of inhibitor metabolite I calculated as the sum of all non-reversible reactions producing I. For α v_I_  = v_max,r_ the above constraints can be satisfied only if flux through reaction v_I_ is 0. For α v_I_ in the range of (0, v_max,r_) the v_r_ is constrained to the range of (0,  α v_I_). Thus, reaction v_r_ is not affected by I if I is not producible, while any increase of producibility of I results in a decreasing upper bound of v_r_, eventually limiting v_r_ to zero.

Our formulation of inhibitor constraint is different from the solution proposed by Vardi and colleagues (3). In their formulation, v_I_ is the flux through an additional virtual reaction consuming the inhibitor, while we use the sum of irreversible reaction fluxes making the inhibitor. Moreover, activator and inhibitor constraints were originally proposed to model a signalling network alone. In Use Case I we will show for the first time much wider application to model systems combining gene regulatory, signaling and metabolic reactions at steady state thus providing general capability to construct mechanistic models of metabolic reprogramming by cellular regulatory networks. Last but not least, MUFINS is the first software providing reaction formula interface, which does not require coding of the additional LP constraints by the user. The reaction formulas can be used both in JyMet GUI and sfba input files in command line mode.

# Extended Quasi Steady State Petri Net (QSSPN) algorithm.

We will introduce Quasi Steady State Petri Net (QSSPN) algorithm and define new features published for the first time in this manuscript. While definition of basic QSSPN is available in the supplementary material of our previous paper (26), it will be re-iterated here to provide consistent definition of the algorithm. New features, introduced for the first time in this work will be indicated. Documentation on file formats, software operation and QSSPN tutorial are provided in MUFINS1.0_Docs of software distribution.

## 2.1. DEFINITION OF QUASI-STEADY STATE PETRI NET (QSSPN).

The QSSPN model is composed of two disjoint sets of molecular interactions: quasi-steady state fluxes (QSSF) and dynamic transitions (DT). We assume that following interaction activity in DT set the QSSF quickly reach steady state. In Constrained Based Modeling (CBM) field (18) this quasi-steady state assumption has been originally applied to connect stoichiometric models of steady state flux distribution in metabolism with dynamic models of gene regulation. This is justified by timescale separation between gene regulation and signaling. We note that covalent protein modification reactions in signaling networks are also fast compared to transcription & translation timescales and can be included into QSSF set. This is further facilitated by use of linear activator and inhibitor constraints as described in the main manuscript.

In QSSPN the DT set is represented using Petri Net (PN) while, QSSF are modeled using Flux Balance Analysis (FBA). For the sake of presenting a full, self contained description of QSSPN, we will first re-introduce definitions of PN and FBA that can also be found elsewhere (18, 27). In section 2.1.3 we will define QSSPN and in chapter 2.2 describe our QSSPN simulation algorithm and its novel extensions.

### 2.1.1. Petri Nets.

A **standard Petri Net** is a directed bipartite graph, where two types of nodes represent **places** and **transitions**. Directed **edges** connect places to transitions and transitions to places. Since PN is a bipartite graph, there are no edges linking places to places and transitions to transitions. A place linked to a transition with a directed edge originating at the place is described as a **pre-place** of that transition. A place connected to a transition by a directed edge originating at the transition is described as a **post-place** of that transition. Each place of the PN may have a non-negative integer number of **tokens** associated with it. The distribution of tokens over all places in the PN is called **marking** and represents the state of the system. Upon **firing**, transitions change the state of the system by moving tokens from pre-places, to post-places. The number of tokens consumed from the given pre-place is specified by the **weight** of an edge linking the pre-place with the transition. The number of tokens added to the post-place is specified by the weight of the edge linking the transition and post-place. The transition may fire only when it is **enabled** (i.e. when all pre-places contain sufficient number of tokens, specified by a **firing rule)**. The PN formalism is associated with graphical notation, where places and transitions are represented by circles and rectangles respectively, edges are drawn as arrows and tokens are represented as dots or numbers drawn within place symbols. The terminology above is summarised by the following commonly used definition:


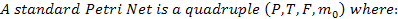


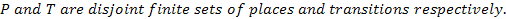


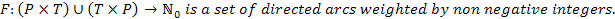


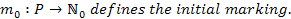


In the PN field, numerous extensions of the standard framework described above have been defined to increase its expressiveness. In QSSPN we use two additional types of edges. A **read edge** connects pre-place to the transition. The transition is enabled only if the pre-place connected by read edge has sufficient number of tokens. However, upon firing, the transition does not consume tokens from the pre-place. In graphical representation read edge is indicated by an arrow headed with a full circle. An **inhibitory edge** connects a pre-place to a transition. The transition is enabled only if the pre-place connected by inhibitory edge **is not** sufficiently marked. In graphical notation an arrow headed with an empty circle represents an inhibitory edge.

The dynamics of the PN is simulated by iteratively firing transitions, which change the marking. A set of firing rules associated with transitions and an algorithm used to generate a dynamic trajectory of the system defines the **Petri Net semantic**. In the simplest case, a transition is enabled when all pre-places connected by standard and read edges are sufficiently marked (i.e. have number of tokens larger than the weight of an edge connecting pre-place to transition) and all pre-places connected by inhibitory edges are not sufficiently marked (i.e. have token numbers lower than the weight of an edge connecting pre-place to transition). The dynamic trajectory is then generated by iteration of the following steps: i) identification of all enabled transitions, ii) random selection of one of the enabled transitions, iii) changing PN marking by firing of selected transition. However, many different semantics are available in PN tools and separation of semantics from connectivity is one of the most powerful features of the PN formalism, supporting testing of different modelling assumptions for the same network connectivity. In **continuous PNs**, firing rules are expressed in terms of Ordinary Differential Equations (ODEs) and standard algorithms for numerical integration of ODEs are used to generate dynamic trajectories. In **stochastic PNs** firing rules are defined by probabilistic propensity functions and the Gillespie algorithm (28) is used to generate a sample of dynamic trajectories. Definition of semantics in continuous and stochastic PNs requires the introduction of **continuous and stochastic transition types**, which, when enabled, are fired by different procedures. Moreover, in the case of continuous PNs, the **continuous place** is defined where real rather than integer number is used as a marking. The PN composed of multiple transition types, including continuous and stochastic transitions is referred to as **hybrid PN**.

### 2.1.2. Flux Balance Analysis (FBA)

**Flux Balance Analysis (FBA)**, frequently referred to as constraint-based method (CBM), explores global flux distribution in a chemical reaction network at steady state. The system of m coupled chemical reactions, involving n metabolites is defined as a stoichiometric matrix S with dimensions of m x n. Each value in the matrix represents the stoichiometric coefficient of a particular metabolite in a particular metabolic reaction. The stoichiometric coefficient is negative for substrates and positive for products. The stoichiometric matrix is sparse, since for most metabolite/reaction pairs the value of the stoichiometric matrix is 0, indicating that metabolite does not participate in the reaction. Vector **x** of length m assigns concentration to every metabolite and vector **v** of length n assigns metabolic flux to every reaction in the network. Time evolution of molecular concentrations in the system is given by the equation: d**x**/dt = S**v**, which at steady state becomes S**v** = **0**. This equation constrains the possible values of fluxes in vector **v**, for each metabolite the sum of fluxes producing this metabolite, must equal the sum of fluxes consuming the metabolite. Additional constraints are introduced by assigning bounds to every reaction: **f_l_** < **v** < **f_u_**, where **f_l_** and **f_u_** are vectors of length n containing lower and upper reaction bounds, respectively. The bounds are used to constrain measured fluxes to experimentally known values and specify reaction reversibility. Reversible reactions can assume both positive and negative flux values, irreversible reactions are constrained to either positive or negative fluxes. Furthermore, the bounds are also used to define boundary conditions (i.e. the values of exchange fluxes, which are connected to sources and sinks of metabolic flux). The sources and sinks are frequently referred to as **external metabolites**, which do not have to be balanced. The external metabolites specify nutrients available and secreted in the medium.

In a genome scale metabolic network the flux values for most of the reactions are unknown, so the linear system given by equation S**v** = **0** cannot be solved for a unique vector **v**. Instead, FBA explores the space of flux distribution vectors **v** that satisfy steady state condition (the null space of S) and the bounds to test feasibility of metabolic functions of interest under given extracellular nutrient conditions. The following linear programming optimisation is performed:


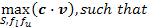


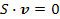


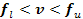


The linear programming (usually Simplex algorithm) is used to maximise **objective function**, which is formulated as a linear combination of fluxes in **v**. Thus, **c** is a vector of objective function coefficients set by the user. The use of linear programming guarantees finding maximal, unique value of objective function, but there may be many solution vectors **v** corresponding to this value. In its original formulation FBA has been used to calculate maximal growth rates of microbial cultures under steady state conditions. However, it can be used to model feasibility of any metabolic function, which is especially applicable in modelling of human cells (11, 12), which may not divide under condition of interest. If the unique, maximal value of objective function is 0, then a particular objective function is not feasible given stoichiometric constraints of 0. In many QSSPN simulations, such as our model of bile acid homeostasis in liver cell (26), we use FBA to test whether metabolites in the network can be produced. Following the previous work of Imielinski (29) and colleagues, and ours (30) on producibility plots we set objective function coefficient vector **c** to the row of stoichiometric matrix corresponding to a metabolite, which is tested, and remove the balance requirement for this metabolite. In this way we maximise sum of fluxes connected to this metabolite. If maximal value of objective function is greater than 0, we conclude that metabolite can be produced by the system. We can also use this calculation to conclude that the flux towards the metabolite is greater under certain conditions rather than others. In this way we can use FBA to test whether the change of metabolic network flux bounds leads to increased availability of the metabolite.

### 2.1.3. Quasi Steady-State Petri Nets (QSSPN)

We define QSSPN as follows:


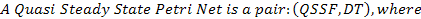
:


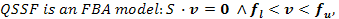


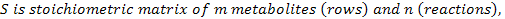


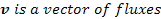


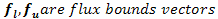


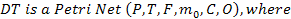


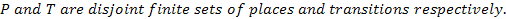


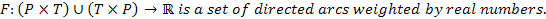


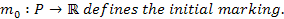


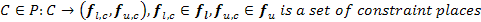


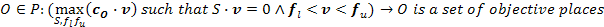


The QSSPN connects the FBA model of Quasi Steady State Fluxes (QSSF) with Petri Net (PN) model of Dynamic Transitions (DT). Connection is defined through two types of PN places. **Constraint places** define mapping between PN marking and FBA bounds making FBA model dependent on the state of PN. **Objective places** define mapping of between multiple FBA objective functions and PN marking making state of PN dependent on FBA model. Constraint place sets specific lower and upper flux bounds in FBA model according to it’s marking. A constraint place can set bounds of multiple fluxes in FBA model specified in vectors f_l,c_ and f_u,c_. Objective place requests evaluation of FBA objective function and sets it’s marking according to objective function value. The objective function is defined as linear combination of FBA fluxes, with coefficients in **c**_O_ vector defining objective specific to particular objective place.

Definition above defines basic QSSPN structure. Chapter 2.2 below defines semantics by introducing transition rules for different transition types, specific rules for constraint and objective places mapping as well as hybrid simulation algorithm. We note that in QSSPN definition above we use PN marking by real rather than integer numbers. This is **new feature**, introduced here to facilitate hybrid simulations where marking can be updated in both discrete and continuous way. In original QSSPN definition (26) we used integer token numbers and approximated continuous changes with very large numbers of discrete levels.

## 2.2 QSSPN SIMULATION ALGORITHM

Here we present QSSPN simulation algorithm that integrates hybrid PNs and FBA. The PNs can be composed using **stochastic**, **continuous**, **immediate** and **reset** transitions. Special **flux** transition type is a **new feature** implemented to communicate fluxes of example FBA solutions to PN part of the model. By graphically selecting different transition types in PN editor the user can implement stochastic, ODE and qualitative models and combine them with FBA. Thus, without any need of additional coding the user can graphically construct models in all major formalisms of computational systems biology and combine these models in multi-formalism simulations.

Below, we provide complete formulation of QSSPN simulation algorithm and indicate new features that were introduced after our first publication (26). We first describe transition semantics and subsequently define a number of functions describing elementary operations within one algorithm iteration. These functions are then used within algorithm flowchart shown on Figure 1.

### 2.2.1 Pre-place activity.

During simulation we use pre-place activity, rather than pre-place marking directly. The user can choose between **mass-action** activity, where activity equals pre-place marking and a **lookup table**, which maps arbitrary activities to marking within specific thresholds. Use of thresholds is key for the formulation of qualitative simulations (26) and for the integration of qualitative and quantitative models.

Activity μ(x) of a pre-place with marking x equals either x, if it is a mass-action activity, or a value from lookup table T thresholds t_i_ and activities a_i_:

In the previous version of the software we used exclusively lookup tables to define activities. The choice between lookup table and mass-action activity is a **new feature** implemented here.

### 2.2.2 Propensity function.

By default, the propensity function of a QSSPN transition is calculated as a product of pre-place activities and rate constant and referred to as **elementary** propensity function. Alternatively, the user can choose **complex** propensity function defined by algebraic expression with pre-place activities as variables. While elementary propensity is a special case of complex propensity, we maintain distinction here as both are implemented differently in the software and input files.

The elementary propensity of PN transition t, denoted by P_t_, is defined as:

where c_t_ is a rate constant, N is the number of pre-places of transition t, x_i_ is the number of tokens at pre-place i and μ_i_ is the pre-place activity in the transition depending on the pre-place state.

The complex propensity of PN transition t, denoted by P_t_ is defined as:

where f is a function of N pre-place activities μ_i_(x) and M parameters c_i_. In current implementation any algebraic expression of activities and parameters can be used. Rising to a power is implemented, trigonometric functions are not yet available.

Use of algebraic expressions to allow definition of complex propensity functions is a major **new feature** of qsspn solver presented here. The formulas are first parsed to Reverse Polish Notation using Dijstra’s shunting-yard algorithm and subsequently efficiently evaluated on a stack during simulation. This is computationally efficient implementation as formulas do not have to be parsed at every iteration of the algorithm.

Use of pre-place activities instead of pre-place states in propensity function formulas is a powerful tool to link variables of models expressed in different formalisms. Activity lookup tables allow introduction of thresholds, which is necessary for integration of quantitative and qualitative models.

### 2.2.3 Transition types.

The QSSPN algorithm uses transition types with the following interpretation of propensity function and update rules (semantics):

*Immediate transition***.** The immediate transition is enabled if its propensity function is greater than 0. Upon firing, it removes integer number of tokens from pre-places connected with standard edge and increases the marking of post-places by integer number. The numbers of tokens being changed upon firing of stochastic transition are specified by stoichiometric coefficients associated with edges.

*Stochastic transition*. The propensity function of stochastic transition is interpreted as the probability density of the transition firing in the next, infinitesimally small, time step. If transition is selected to fire it removes integer number of tokens from pre-places connected with standard edge and increases the marking of post-places by integer number. The numbers of tokens being changed upon firing of stochastic transition are specified by stoichiometric coefficients associated with edges.

*Continuous transition***.** The propensity function of continuous transition is interpreted as the rate. The rates are used as terms of Ordinary Differential Equations, which update marking of places connected to continuous transitions.

*Reset transition***.** Reset transition is enabled if its propensity function is greater than 0. Upon firing reset transition sets, rather than updates, marking of its post-places. If reset function is defined the marking of post-places is set to its value. Reset function can be defined as algebraic expression of pre-place markings (**new feature**). If reset function is not defined each post-place is set to the value of a number defined by an edge linking the place to reset transition. A place can be a connected as a post-place to only one reset transition.

*Flux transition.* This is **new feature** introduced to allow use of an example solution of a particular FBA objective function. This feature is necessary for implementation of dynamic FBA method. A flux transition can have only one pre-place and this pre-place must be an objective place. A flux transition can have only one post-place. Upon objective function evaluation, flux transition sets marking of its post-place to the value of a specific flux in FBA solution of the objective function.

### 2.2.4 Setting of flux bounds (Function: setQSSFbounds()).

This function sets the bounds of fluxes in the quasi-steady state flux (QSSF) part of the model according to the state of the state of constraint place. Each constraint place is associated with a list of fluxes in the QSSF and a lookup table setting bounds of all fluxes from this list according to the place’s state. The lookup table has the following form:

where x is marking of the constraint place, lb(x), ub(x) are lower and upper flux bounds respectively, t_1_,..,t_T_ are marking thresholds and f_l,i_, f_u,i_ are lower and upper flux bound values set according to the objective place’s state. If application of the lookup table results in a change to the lower or upper bound for any of the fluxes, the global Boolean variable ‘UpdateRequired’ is set to TRUE to indicate that flux balance analysis (FBA) objective functions need to be evaluated. The UpdateRequired variable is re-set to FALSE before the loop over constraint nodes commences.

### 2.2.5 Evaluation of an objective function (Function: evaluateObjective())

This function uses Flux Balance Analysis to evaluate the objective function specified by a particular objective node. The objective function is specified as the name of the flux or the name of the metabolite in the QSSF network. If the objective is specified as the flux the linear programming (LP) maximises value of this flux. If the objective is specified as a metabolite, the sum of fluxes producing this metabolite is maximized (metabolite producibility (29, 30)). If ‘UpdateRequired’ variable is set to TRUE the objective is evaluated by LP maximisation of entire QSSF model. Otherwise, the value determined for this objective in the previous iteration is used. Evaluation of an objective function by LP maximisation of the entire QSSF model is the most computationally demanding step of the QSSPN algorithm. The use of ‘UpdateRequired’ variable ensures that this step is not executed unnecessarily, when QSSF bounds have not changed, and the previously determined value of the objective function would be obtained.

### 2.2.6 Marking of objective place (Function: updateObjectivePlace())

This function sets the marking of a particular objective place according to the objective function value, thus feeding back information about steady state metabolic capabilities to the dynamic part of the model. The following lookup table is used to set the marking of the objective place:

where x(o) is the state of the objective node, t_1_,..,t_T_ are objective function thresholds (real numbers) and s_1_,..,s_T_ are integer number of tokens assigned to the node, depending on the objective function value *o* calculated by evaluateObjective().

### 2.2.7. Firing of reset transitions (Function: fireResetTransitions() new feature).

Propensity function of all reset transitions is evaluated as described in 2.2. Subsequently, all reset transitions for which propensity function is greater than 0 are fired. If two reset transitions are connected to the same post-place, simulation is stopped and error message is returned to the user. We allow reset transitions to act on only one place.

### 2.2.8. Firing of immediate transitions (Function: fireImmediateTransitions()).

Propensity function of all immediate transitions is evaluated as described in 2.2. Subsequently, all immediate transitions with propensity function greater than 0 are fired. The local conflicts between multiple immediate transitions connected to the same place are resolved by the following stochastic approach. Let T_P_ be the set of enabled transitions connected to place P. We randomly select transition t from T_p_ in such a way that probability of transition being selected is proportional to its propensity function. Only transition t changes marking of P.

### 2.2.9. Firing of continuous transitions (Function: fireContinuousTransitions(Δt_max_)).

Propensity function of all continuous transitions is evaluated as described in 2.2. Subsequently, marking of all places connected to continuous transitions is updated. Let T_p_ be the set of n transitions connected to place P. The m_p_, marking of place p is changed in the following way:


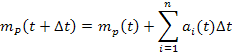


where a_i_(t) is propensity of transition i at time current simulation time t. The timestep Δt is selected in such a way that max(a_1_(t)Δt /m_p_(t),…, a_1_(t)Δt /m_p_(t)) < maxChange. Default value of maxChange is 0.01, but user can change this variable. If Δt is greater than Δt_max_ then Δt is set to Δt_max_. We note that this implies that the PN composed exclusively of continuous transitions is interpreted as a system of coupled Ordinary Differential Equations and numerically integrated with adaptive timestep Euler algorithm. Our case studies show that this algorithm provides practical solution for stiff systems and contrary to more complex ODE integration algorithms is easy to integrate into hybrid simulation. In the future versions of the software we will investigate further improvements to computational efficiency of continuous transition firing. We also note that this function has been substantially improved since previous version of the software (**new feature**).

### 2.2.10. Firing of delayed transitions (Function: fireDelayedTransition(t)).

The function examines delayed transitions queue to check if there are any scheduled to be fired before time t. If there are no delayed transitions scheduled to fire the function returns 0. Otherwise, it fires one delayed transition, removes this transition from delayed transition queue and returns t_s_, the time at which this transition has been scheduled. If multiple transitions are scheduled to be fired before time t, only the transition, which is scheduled at the earliest time, is fired.

### 2.2.11. Scheduling of delayed transitions (Function: scheduleDelayedTransition(m))

If the stochastic transition m selected to be fired has delay time t_d_ greater than 0 this function adds the transition to the delayed transitions queue. The transition is scheduled to fire at time t_s_ = t + t_d_.

### 2.2.12. Firing of flux transitions (Function: fireFluxTransitions()).

This function fires all flux transitions associated with an objective place o, after evaluateObjective() function has been fired for this place. The place o is the only pre-place of each of these transitions. Each flux transition has only one postplace. Upon firing flux transition sets marking of it’s postplace to one of the example fluxes in FBA solution obtained for objective place o. This allows use of example flux values in subsequent iteration of the algorithm. This **new feature** is required for implementation of the original dFBA method (31).

### 2.2.13 QSSPN hybrid simulation algorithm.

Figure S14: QSSPN simulation algorithm.

Functions used in this diagram are defined in 2.2.4-2.2.12

## 2.3. DISTRIBUTED QUALITATIVE SIMULATION WITH QSSPNclient.

Distributed simulation is a major **new feature** of qsspn solver. We have implemented QSSPNclient, a client-server version of qsspn solver. The server loads QSSF from sfba file and waits for requests from multiple clients. Clients load DT models from qsspn files and run QSSPN algorithm. Whenever evaluateObjective() function (see 2.2.5) is called client sends whole set of QSSF bounds to the server and retrieves value of the objective function. The server tabulates objective function value for a complete set of QSSF bounds. If any of the clients requests evaluation of the same objective for the same set of bounds as tabulated before, objective function value is returned without executing the most computationally costly step, which is LP evaluation on QSSF network.

To evaluate performance gain of the QSSPNclient, we have simulated our model of bile acid homeostasis in human hepatocyte, used to validate first QSSPN implementation (26). This is qualitative model, where we generate a Monte Carlo sample of trajectories feasible given DT network connectivity and QSSF constraints. The numbers of trajectories exhibiting qualitative behavior of interest in the original and perturbed model are compared to determine whether perturbation affects the behavior (see (26) for details). Here we first computed 100 trajectories using current qsspn implementation. The calculations run on a single core Intel I7 2.6 GHz CPU took 325 minutes. Subsequently, we started the instance of a server with HepatoNet1 (12) – a QSSF part of a model. Calculation of 100 trajectories with QSSPNclient took 217 minutes. We used the same random number generator seed in both simulations and confirmed that QSSPNclient generated the same results as stand-alone implementation. Thus, the simulation was 1.5 times faster if client server architecture was used and, in practical terms, we gained close to two hours by using QSSPNclient to run this simulation. This will translate to even more substantial gains in simulations of multiple perturbations, such as genotype/phenotype scan presented in our previous work (32). Also, multiple users running different DT models connected to the same QSSF will benefit from client/server implementation.

The QSSFclient does not implement flux transitions. Implementation of flux transition would require storing of full FBA solutions obtained for particular set of bounds and objective function, rather than objective function value alone and decrease the number FBA bounds/solution pairs that can be tabulated. On the other hand, the dFBA simulations are fast, because only one objective place is required. Also, in quantitative simulations, where the dFBA is connected to ODE model of regulatory network (e.g. Use Case 2 in main manuscript), only one trajectory is generated. Thus, implementation of flux transitions would not increase performance gain, but would make QSSPNclient implementation more complex and less efficient.

In conclusion, we recommend using QSSPNclient in qualitative simulations where Monte Carlo sampling of qualitative dynamic trajectories is used to explore behavior of DT part of the model and where multiple objective functions are used to explore capabilities of the QSSF.

# Fast iMAT algorithm

The iMAT approach (33) searches for flux distributions that satisfy both stoichiometric and thermodynamic constraints of the Genome Scale Metabolic Network (GSMN), and maximize congruency with transcriptome data. Essentially, the objective function is defined as the number of reactions where the reaction flux is consistent with the transcriptional activity of the genes encoding the enzymes required for that reaction. Gene expression data is first discretised into two (transcribed, not transcribed) or three (transcribed, neutral, not transcribed) categories. Next, the discretized gene expression levels, and Boolean rules associating genes and reactions in the GSMN are used to classify reaction states as active, inactive or neutral. Finally, MILP is used to find flux distributions where the maximal number of active reactions are assigned fluxes greater than the predefined positive flux threshold, while reaction fluxes associated with inactive reactions are minimized. While the objective function value (number of reactions which state agrees with transcriptome data) is unique, there will exist many possible solutions that lead to the same maximal objective function value. To provide a unique solution for individual reaction, the method calculates the objective function value when a particular reaction is constrained to both the active inactive states. The difference between these two values is a unique reaction activity. The final output of the iMAT method gives each reaction an integer number, which measures the flux activity of the reaction.

The iMAT approach is computationally intensive, as two MILP solutions must be derived for each reaction within the GSMN. This means that for larger GSMNs, such as Recon2 (11), it is impractical to run this method on large number of transcriptome datasets. To address this issue, we have modified the iMAT method, such that only one MILP problem is solved, increasing the computational efficiency; we term this approach Fast iMAT. In Fast iMAT, discretised gene expression data and gene-protein-reaction association rules of GSMN model are used to assign a state
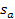
 to every reaction as
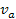
 in the model. The active reactions are assigned state of 1 while inactive reactions state of -1. Remaining reactions are assigned the state of 0. Subsequently, reactions with non-negative lower flux bound (
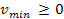
) are classified as forward reactions denoted as
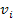
 with state
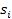
 where
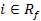
 (forward reaction set). Reactions with non-positive upper bound (
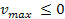
) are classified as reverse reactions and denoted as
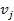
 with state
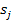
 where
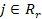
 (reverse reaction set). Reversible reactions (
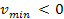
 and
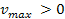
) are denoted as
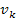
 with state
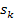
 where
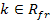
 (reversible reaction set). Each reversible reaction is divided into a forward direction denoted
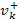
, and a reverse direction denoted
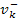
 , both using the coefficient
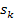
. Derivation of the optimal flux distribution using the Fast iMAT method has the same principle as the original method, but only one MILP problem must be solved, as defined by the following equations:


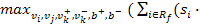

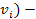

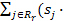

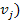

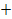

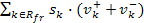

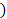
 [1]

subject to the constraints:


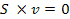
,
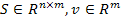
 [2]


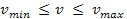
 [3]


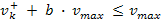
 [4]


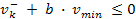
 [5]


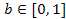
 [6]

Eqn 1 defines the objective function of the MILP problem. Eqn 2 and 3 define the stoichiometric constraints and flux bounds, where
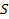
 is a
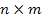
 stoichiometric matrix with
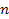
 metabolites and
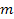
 reactions whose fluxes are presented as vector
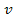
. Eqns 4-6 add additional constraints and use one Boolean control variable,
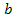
, to ensure that for every forward/reverse reaction pair only one of the reactions is active at any point, thus preventing futile cycles. The solution of this MILP problem provides fluxes for all nonzero-state reactions, and then standard flux variability analysis (FVA) can be applied to the remaining reactions in the GSMN, with the solution space constrained by the previously determined nonzero-state reaction fluxes. The flux range
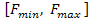
predicted by this method can be used to assign integer activities of all reactions used in original iMAT. If
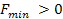
 or
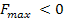
 then the reaction is predicted to be active; if
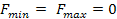
 then the reaction is predicted to be inactive; otherwise, the reaction is predicted undetermined. However, contrary to iMAT these fluxes values can be used directly, without discretisation, which is useful for well-parameterised models. Comparison of Fast iMAT with iMAT is presented in Use case 3 of main manuscript.

# COMPARISON OF TOOLS AVAILABLE WITHIN MUFINS WITH EXISTING SOFTWARE

# Multi-formalism simulation in MUFINS

Use Case 2 of main manuscript shows that MUFINS can integrate quantitative dynamic Ordinary Differential Equation (ODE) models with Constrained Based Models of Genome Scale Metabolic Networks (GSMN). In our previous paper we applied first version of qsspn solver to combine qualitative PN model of gene regulation and signaling with the GSMN (26). The sampling of alternative event sequences has been performed with Gillespie algorithm, where all transition rates were set to 1. The introduction of mass action kinetics and complex rate laws to current version of QSSPN allows implementation of quantitative stochastic kinetic models. Thus, QSSPN can integrate exact stochastic kinetic simulations with CBM, to allow simulations of both qualitative and quantitative dynamic models, integrated with GSMNs in quasi-steady state approximation. In our previous paper (26) we have also demonstrated that PN can be used to represent Boolean dynamic model and implement rFBA (34) simulation. Moreover, in Use Case 1, we show that logical steady state models can be represented as linear constraints and included into GSMN part of the model. While deterministic and stochastic kinetic simulations have been already integrated in hybrid algorithms (35) and software (36), there is no general software allowing integration of all the formalisms described above. Therefore, **MUFINS is the only software supporting integration of i) exact stochastic, ii) ODE, iii) qualitative dynamic, iv) logical steady state and v) CBM models in a general software platform with GUI.**  The only alternative to achieve the range of formalisms that can be integrated with MUFINS is coding of the model in mathematical modeling environment. While this strategy has achieved spectacular success (37), it is not a plausible proposition for experimental scientists, who do not have programming skills. Moreover, multiformalism modeling in mathematical language involves implementation of the simulation algorithm dedicated to a particular model. In MUFINS, each model is run in the same QSSPN simulation algorithm presented as Figure S9. The multi-formalism functionality emerges from the interactions between the different types of Petri Net places and transitions that can be graphically assembled, leading to combinatorial diversity of types of models that can be simulated. Using one algorithm and a few well-defined place and transition types provides clearer control and description of model assumptions than coding a different main simulation loop for each model. Also, the algorithms available in MUFINS are validated and optimized against the legacy of previous applications, while formulation, validation and description of a simulation algorithm dedicated to a particular model will take additional time. It is our experience that even scientists who can program will find it easier to implement complex multi formalism models by connecting QSSPN places and transitions to off-the-shelf GSMN models imported to JyMet, rather then by development of dedicated mathematical modeling code.

# Comparison with major software

To further substantiate our statements we used the compilation of 146 CBM methods and 27 software packages used by Lewis, Nagarajan and Palsson to construct their “phylogeny of *in silico* methods” (38). We updated the compilation by adding 8 methods and 3 software packages as well as 11 software interface and dependency features. Subsequently, we have annotated all 165 software features in 30 software packages. Results are shown in Supplementary Table 4. To the best of our knowledge this is the most comprehensive comparison of CBM software features to date. Lewis and colleagues (38) did not annotate software with methods, but rather presented two separate lists. We note that this review includes all tools in which CBM has been integrated with other formalism and would therefore highlight any existing method and/or software integrating CBM with multiple other formalisms. Therefore, this unique compilation can be used to both investigate whether MUFINS is indeed unique multi-formalism simulations tool as well as to compare its considerable support for CBM with other software.

**Supplementary Table 4 clearly confirms that there is no other algorithm and software capable of integrating GSMNs with all formalisms that can be expressed by QSSPN method.** Our use cases and previous work demonstrate that MUFINS can be used for simulations equivalent to iFBA(39), idFBA(40), dFBA (31) and rFBA(34). Moreover, linear inhibitor constraints can be used to efficiently implement SR-FBA approach (41), i.e. integrate steady state logical models with GSMNs. There is no software integrating stochastic dynamic simulations with CBM. None of the tools can perform hybrid simulations integrating all these algorithms as it can be done with QSSPN in MUFINS.

To evaluate CBM features of MUFINS, we selected all software packages that implemented more than 10 approaches and grouped features into 12 categories reflecting the classification in (38) and the major features of the software interface and dependencies (Table 1 below). MUFINS is the second most general CBM software after COBRA toolbox in terms of the number of methods implemented. However, it is the most general software with an interactive GUI, providing the largest set of CBM methods for users who are not familiar with a mathematical modeling environment. It is also the most general software providing visualization of results on interactive pathway maps. MUFINS still does not provide automatic strain design algorithms, although properties of microbial strains can be tested with comprehensive set of CBM approaches. Previous versions of sfba solver have been already applied for bioprocess design(42). We also note that QSSPN is an ideal approach to test designed strains in dFBA simulations of batch cultures or more sophisticated models where bioreactor flows would be modeled in the same way as physiological compartment flows were modeled in Use Case 2. Mechanistic interpretation of transcriptomic and proteomic data in the context of GSMN is a highly active field of CBM. MUFINS provides 3 methods for such analysis that are not implemented in other software packages. First, as demonstrated through Use Case 3 our Fast iMAT algorithm provides useful biological insight from the analysis of clinical transcriptome data in the context of the Recon2 human GSMN (11). We also show that this analysis is not practical by the original iMAT method. Second, we provide implementation of our Differential Producibility Analysis (30), which is not available in any other software. Third, we implement the Gene Nutrient Interaction method, which was formulated by Diamant and colleagues (43), but is available only as the source code and not in any regular software. We have already applied MUFINS implementation of this approach to analyse Next Generation Sequencing data from a transposon gene essentiality screen of *M. tuberculosis* interacting with dendritic cells. Our results provided insight into the pathogen’s nutritional requirements during interaction with host cells (44).

### Table S2. Comparison of CBM features of MUFINS with other software.

| Feature | COBRA toolbox | MUFINS | FASIMU | CellNetAnalyzer | OptFlux | COBRApy | TIGER |
| --- | --- | --- | --- | --- | --- | --- | --- |
| No. of methods | 26 | 24 | 17 | 16 | 16 | 12 | 12 |
| Matlab toolbox | X |  |  | X |  |  | X |
| Menu interface |  | X |  | X | X |  |  |
| Layout editor |  | X | X |  | X |  |  |
| Automatic layout |  | X | X |  |  |  |  |
| Pathway image | X |  |  | X |  |  |  |
| Command line |  | X | X |  |  | X |  |
| EFM, cut sets |  |  |  | X |  |  |  |
| FBA | X | X | X | X | X | X | X |
| Omic constraints | X | X | X |  |  |  | X |
| Regulation | X | X |  |  |  |  |  |
| Strain design |  |  |  |  | X |  |  |
| Thermodynamics |  |  | X |  |  |  |  |

# References

1. Papin, J.A., et al., *Reconstruction of cellular signalling networks and analysis of their properties.* Nature Reviews Molecular Cell Biology, 2005. **6**(2): p. 99-111.

2. Dasika, M.S., A. Burgard, and C.D. Maranas, *A computational framework for the topological analysis and targeted disruption of signal transduction networks.* Biophysical Journal, 2006. **91**(1): p. 382-398.

3. Vardi, L., E. Ruppin, and R. Sharan, *A Linearized Constraint-Based Approach for Modeling Signaling Networks.* Journal of Computational Biology, 2012. **19**(2): p. 232-240.

4. Oda, K. and H. Kitano, *A comprehensive map of the toll-like receptor signaling network.* Molecular Systems Biology, 2006. **2**.

5. Raza, S., et al., *Construction of a large scale integrated map of macrophage pathogen recognition and effector systems.* Bmc Systems Biology, 2010. **4**.

6. Klamt, S., et al., *A methodology for the structural and functional analysis of signaling and regulatory networks.* Bmc Bioinformatics, 2006. **7**.

7. Klamt, S., J. Saez-Rodriguez, and E.D. Gilles, *Structural and functional analysis of cellular networks with CellNetAnalyzer.* Bmc Systems Biology, 2007. **1**.

8. Kolodkin, A., et al., *Optimization of stress response through the nuclear receptor-mediated cortisol signalling network.* Nature Communications, 2013. **4**: p. 1972.

9. Kolodkin, A.N., et al., *Design principles of nuclear receptor signaling: how complex networking improves signal transduction.* Molecular Systems Biology, 2010. **6**: p. 446.

10. Chelliah, V., et al., *BioModels: ten-year anniversary.* Nucleic Acids Research, 2015. **43**(D1): p. D542-D548.

11. Thiele, I., et al., *A community-driven global reconstruction of human metabolism.* Nature Biotechnology, 2013. **31**(5): p. 419.

12. Gille, C., et al., *HepatoNet1: a comprehensive metabolic reconstruction of the human hepatocyte for the analysis of liver physiology.* Molecular Systems Biology, 2010. **6**: p. 411.

13. Jain, M., et al., *Metabolite Profiling Identifies a Key Role for Glycine in Rapid Cancer Cell Proliferation.* Science, 2012. **336**(6084): p. 1040-1044.

14. Krauss, M., et al., *Integrating Cellular Metabolism into a Multiscale Whole-Body Model.* PloS Computational Biology, 2012. **8**(10).

15. Sohlenius-Sternbeck, A.-K., *Determination of the hepatocellularity number for human, dog, rabbit, rat and mouse livers from protein concentration measurements.* Toxicology in Vitro, 2006. **20**(8): p. 1582-1586.

16. Kolker, E., et al., *MOPED: Model Organism Protein Expression Database.* Nucleic Acids Research, 2012. **40**(D1): p. D1093-D1099.

17. Guo, T., et al., *Rapid measurement of estrogens and their metabolites in human serum by liquid chromatography-tandem mass spectrometry without derivatization.* Clinical Biochemistry, 2008. **41**(9): p. 736-741.

18. Bordbar, A., et al., *Constraint-based models predict metabolic and associated cellular functions.* Nature Reviews Genetics, 2014. **15**(2): p. 107-120.

19. Curtis, C., et al., *The genomic and transcriptomic architecture of 2,000 breast tumours reveals novel subgroups.* Nature, 2012. **486**(7403): p. 346-352.

20. Leoncikas, V., et al., *Generation of 2,000 breast cancer metabolic landscapes reveals a poor prognosis group with active serotonin production.* Scientific Reports, 2016. **6**: p. 19771.

21. Gentleman, R.C., et al., *Bioconductor: open software development for computational biology and bioinformatics.* Genome Biology, 2004. **5**(10): p. R80.

22. Racine, J.S., *RStudio: A Platform-Independent IDE for R and Sweave.* Journal of Applied Econometrics, 2012. **27**(1): p. 167-172.

23. Dunning, M.J., et al., *beadarray: R classes and methods for Illumina bead-based data.* Bioinformatics, 2007. **23**(16): p. 2183-2184.

24. DiNatale, B.C., et al., *Kynurenic Acid Is a Potent Endogenous Aryl Hydrocarbon Receptor Ligand that Synergistically Induces Interleukin-6 in the Presence of Inflammatory Signaling.* Toxicological Sciences, 2010. **115**(1): p. 89-97.

25. Opitz, C.A., et al., *An endogenous tumour-promoting ligand of the human aryl hydrocarbon receptor.* Nature, 2011. **478**(7368): p. 197-203.

26. Fisher, C.P., et al., *QSSPN: Dynamic Simulation of Molecular Interaction Networks Describing Gene Regulation, Signalling and Whole-Cell Metabolism in Human Cells.* Bioinformatics, 2013. **29**(24): p. 3181-3190.

27. Breitling, R., et al., *A structured approach for the engineering of biochemical network models, illustrated for signalling pathways.* Briefings in Bioinformatics, 2008. **9**(5): p. 404-421.

28. Gillespie, D.T., *Exact stochastic simulation of coupled chemical-reactions.* Journal of Physical Chemistry, 1977. **81**(25): p. 2340-2361.

29. Imielinski, M., et al., *Investigating metabolite essentiality through genome-scale analysis of Escherichia coli production capabilities.* Bioinformatics, 2005. **21**(9): p. 2008-2016.

30. Bonde, B.K., et al., *Differential Producibility Analysis (DPA) of Transcriptomic Data with Metabolic Networks: Deconstructing the Metabolic Response of M. tuberculosis.* PLoS Computational Biology, 2011. **7**(6).

31. Varma, A. and B.O. Palsson, *Stoichiometric flux balance models quantitatively predict growth and metabolic by-product secretion in wild-type Escherichia-coli w3110.* Applied and Environmental Microbiology, 1994. **60**(10): p. 3724-3731.

32. Fisher, J., et al., *Predictive Modeling of signaling crosstalk during C-elegans vulval development.* Plos Computational Biology, 2007. **3**(5): p. 862-873.

33. Shlomi, T., et al., *Network-based prediction of human tissue-specific metabolism.* Nature Biotechnology, 2008. **26**(9): p. 1003-1010.

34. Covert, M.W., C.H. Schilling, and B. Palsson, *Regulation of gene expression in flux balance models of metabolism.* Journal of Theoretical Biology, 2001. **213**(1): p. 73-88.

35. Puchalka, J. and A.M. Kierzek, *Bridging the gap between stochastic and deterministic regimes in the kinetic simulations of the biochemical reaction networks.* Biophysical Journal, 2004. **86**(3): p. 1357-1372.

36. Hoops, S., et al., *COPASI — a COmplex PAthway SImulator.* Bioinformatics, 2006. **22**: p. 3067-3074.

37. Karr, J.R., et al., *A Whole-Cell Computational Model Predicts Phenotype from Genotype.* Cell, 2012. **150**(2): p. 389-401.

38. Lewis, N.E., H. Nagarajan, and B.O. Palsson, *Constraining the metabolic genotype-phenotype relationship using a phylogeny of in silico methods.* Nature Reviews Microbiology, 2012. **10**(4): p. 291-305.

39. Covert, M.W., et al., *Integrating metabolic, transcriptional regulatory and signal transduction models in Escherichia coli.* Bioinformatics, 2008. **24**(18): p. 2044-2050.

40. Lee, J.M., et al., *Dynamic analysis of integrated signaling, metabolic, and regulatory networks.* Plos Computational Biology, 2008. **4**(5).

41. Shlomi, T., et al., *A genome-scale computational study of the interplay between transcriptional regulation and metabolism.* Molecular Systems Biology, 2007. **3**.

42. Khannapho, C., et al., *Selection of objective function in genome scale flux balance analysis for process feed development in antibiotic production.* Metabolic Engineering, 2008. **10**(5): p. 227-233.

43. Diamant, I., et al., *A network-based method for predicting gene-nutrient interactions and its application to yeast amino-acid metabolism.* Molecular Biosystems, 2009. **5**(12): p. 1732-1739.

44. Mendum, T.A., et al., *Lipid metabolism and Type VII secretion systems dominate the genome scale virulence profile of Mycobacterium tuberculosis in human dendritic cells.* Bmc Genomics, 2015. **16**.
