# Supplementary material for: MUFINS: multi-formalism interaction network simulator
Source: NPJ Syst Biol Appl. 2016 Nov 17;2:16032–. doi: 10.1038/npjsba.2016.32 (PMC5516860; doi:10.1038/npjsba.2016.32)
Supplement: GETTING_STARTED [file npjsba201632-s2.doc]

# Getting Started with MUFINS

Table of Contents

[Overview of MUFINS 2](#__RefHeading___Toc450650050)

[Constraint Based Modelling 3](#__RefHeading___Toc450650051)

[Exercise 1. Calculation of growth rate on different media 3](#__RefHeading___Toc450650052)

[Genome Scale Metabolic Reaction Network of Escherichia coli. 3](#__RefHeading___Toc450650053)

[Import SBML file into JyMet. 3](#__RefHeading___Toc450650054)

[Saving models as a MUFINS reaction table, and editing with an external text editor. 4](#__RefHeading___Toc450650055)

[Open a MUFINS sfba model in JyMet. 5](#__RefHeading___Toc450650056)

[Closing a model 6](#__RefHeading___Toc450650057)

[Define the external metabolites for the GSMN 6](#__RefHeading___Toc450650058)

[The Biomass reaction and it’s relationship to growth rate. 6](#__RefHeading___Toc450650059)

[Calculate the maximal growth rate 7](#__RefHeading___Toc450650060)

[Simulate the effect of different media conditions on growth rate 9](#__RefHeading___Toc450650061)

[Exercise 2. Essential reactions. 10](#__RefHeading___Toc450650062)

[Exercise 3. Using the visualisation tools within JyMet 11](#__RefHeading___Toc450650063)

[Open the model and prepare it for simulation. 11](#__RefHeading___Toc450650064)

[Open the problem file 12](#__RefHeading___Toc450650065)

[Run Flux Balance Analysis 13](#__RefHeading___Toc450650066)

[Preparing the results table for visualisation 13](#__RefHeading___Toc450650067)

[Generating a visualisation in JyMet 14](#__RefHeading___Toc450650068)

[Quasi Steady State Petri Net (QSSPN) 17](#__RefHeading___Toc450650069)

[Exercise 1: QSSPN simulation of cortisol signaling in the liver 18](#__RefHeading___Toc450650070)

[File Structure for a QSSPN simulation 18](#__RefHeading___Toc450650071)

[Use of Snoopy to create Petri net representations of signaling networks 20](#__RefHeading___Toc450650072)

[Markup required within the Snoopy file of a QSSPN simulation 21](#__RefHeading___Toc450650073)

[Introduction of a perturbation using Snoopy 24](#__RefHeading___Toc450650074)

[Running a QSSPN simulation 25](#__RefHeading___Toc450650075)

[QSSPN Semantics Guide 28](#__RefHeading___Toc450650076)

[QSSPN file format 28](#__RefHeading___Toc450650077)

[QSSPN control file format 31](#__RefHeading___Toc450650078)

[Analysis of ~omics data in the context of genome-scale metabolic networks 33](#__RefHeading___Toc450650079)

[References 47](#__RefHeading___Toc450650080)

# Overview of MUFINS

MUFINS is a Multi-Formalism Interaction Network Simulator. It contains a suite of 24 tools that allow a wide variety of analysis methodologies to be undertaken, and is bundled with JyMet, a Graphical User Interface (GUI) to allow MUFINS to be used by the specialist and no specialist alike. The tools within MUFINS can be roughly divided into the following categories:

Flux Balance Analysis: FBA, phenotypic phase plane (PhPP), dynamic FBA (dBA), flux variability analysis (FVA), differential producibility analysis (DPA), Fermentation equation (FBA precursor)

Gene/Reaction Perturbation: Gene deletion analysis, Gene-Nutrient interactions

~omics constraints: Gene inactivity moderated by metabolism and expression (GIMME), Shlomi_NBT_08 (iMAT), Fast iMAT

Regulation: Regulatory FBA (rFBA), linear inhibitor and activator constraints, integrated dynamic FBA (idFBA), integrated FBA (iFBA), quasi steady-state Petri nets (QSSPN)

MUFINS incorporates three important innovations, providing simulation possibilities beyond those available in other software suites. These are: (i) linear inhibitor and activator constraints, allowing the integration of regulatory networks, including both activation and inhibition reactions into a CBM. (ii) Fast iMAT to allow the use of ~omic data to provide additional constraints for CBM. Fast iMAT is an optimised algorithm that is highly computationally efficient and allows the interrogation of large-scale ~omic datasets. (iii) Quasi steady-state Petri nets (QSSPN) allow the integration of gene and signalling regulatory networks with genome-scale metabolic networks, facilitating the simulation of dynamic biological responses to stimuli.

A full description of these tools, plus the comparison of MUFINS with other software suites is available in the primary publication for MUFINS.

# Constraint Based Modelling

The following exercises are designed to provide a quick introduction to Constraint Based Modelling with MUFINS. All the necessary files for the simulations are bundled with the MUFINS distribution, and can be found in the ‘MUFINS_Examples’ folder. Full instructions on how to install MUFINS can be found in both the ‘Readme.txt’ in the root folder and ‘Installation’ help file.

## Exercise 1. Calculation of growth rate on different media

A common requirement in constraint based modelling is to predict the growth rate for a cell population (1). For cell division to occur, the cell must first produce all the components required to duplicate itself (e.g. nucleotides, amino acids etc.), which is a process of metabolism. Predicting the rate at which these components can be made provides a maximal rate at which the cell could divide, and hence a measure of the rate of *biomass* production. This is a key feature of prokaryotic cells, where the aim is to continually divide and expand the cell number, but also of mammalian disease such as cancer. Even for human cells, which tend to be tightly regulated with regard to cell division, there is still a need for biomass production to allow the renewal of the cellular pool, replacing those cells that have reached the end of their lives.

### Genome Scale Metabolic Reaction Network of Escherichia coli.

In this exercise, we use the Genome Scale Metabolic Network of *Escherichia coli* developed by the group of Berhard Palsson (2):

The folder ‘MUFINS🡺Examples🡺Ec_iAF1260’ contains a single file Ec_iAF1260_flux1.xml. This file was downloaded from the supplementary material of original publication and is in Systems Biology Markup Language (SBML, <http://sbml.org/Main_Page>) format. The bounds of transport reactions are set to represent minimal medium with glucose as a carbon source. The model is called iAF1260 in an attempt to create naming convention of ‘*in silico’* microbial strains. The ‘i’ stands for ‘in silico’, AF are the initials of the first author, while 1260 is the number of genes covered by the model.

### Import SBML file into JyMet.

JyMet is the graphical user interface (GUI) for MUFINS. To open JyMet, enter the ‘MUFINS’
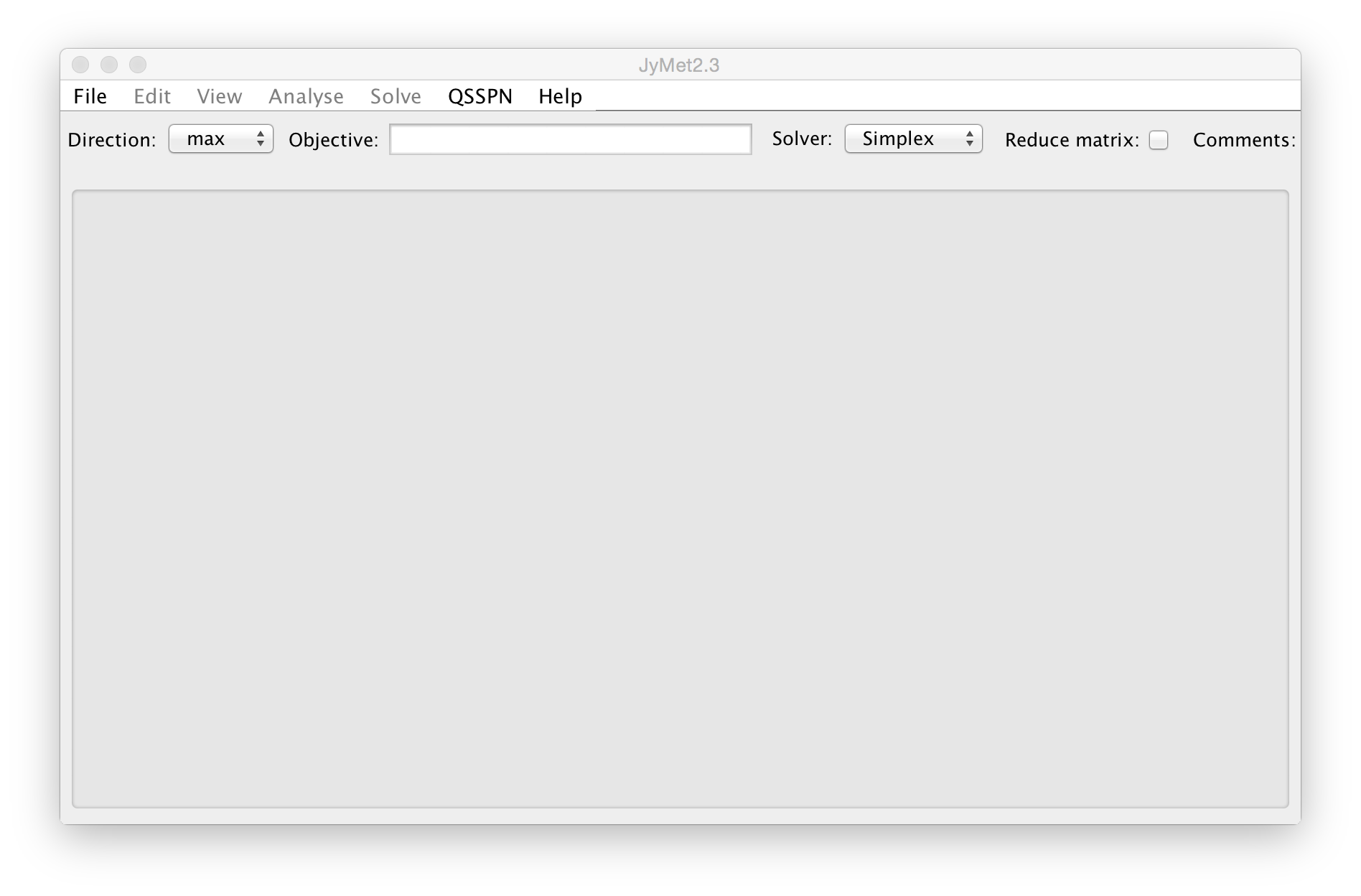
directory and double click JyMet.jar. After a few seconds, the JyMet interface will open (Figure 1).

Figure 1: The JyMet Graphical User Interface for MUFINS

The Systems Biology Markup Language (SBML) is the widely used standard language for model reconstruction. MUFINS is able to load genome scale metabolic networks presented in SBML. These can then be either simulated directly, or converted into the MUFINS-specific sfba format.

To import the SBML file for *E.coli* model to be used in this exercise, select ‘File🡺Import SBML’, navigate to the ‘MUFINS🡺Examples🡺Ec_iAF1260’ folder, and select *Ec_iAF1260_flux1.xml.* Once the model loads, you will see several tabs that describe the model in detail. These are: genes, enzyme, reactions and metabolites. The number in each tab shows the number of entries in each of the tabs.

For example, in figure 2, the reaction tab for the Ec_iAF1260 model can be seen. This tab contains 2382 reactions, one per line. For each reaction, up to 6 columns of information are presented

*ID*: Unique ID for the reaction

*Equation:* An algebraic description of the reaction. ‘M_*’ refers to a metabolite (further defined in the metabolites tab), while ‘+’ and ‘=’ define the relationship between metabolites for this reaction. By convention, reactions are read left to right.

*LB:* Sets the maximal flux rate in the reverse direction (i.e. right to left). In this example, values range from 0.0 or -999999.0

*UB:* Sets the maximal flux rate in the forward direction (i.e. left to right). In this example, values range from from 0.0 or 999999.0

*Rule:* Defines the linkage between genes and reactions

Comment: Contains any free text description of the reaction. Comments can be used to tag reactions of interest, so they can be quickly found in simulation results tables through the search function ‘Edit🡺Search’.


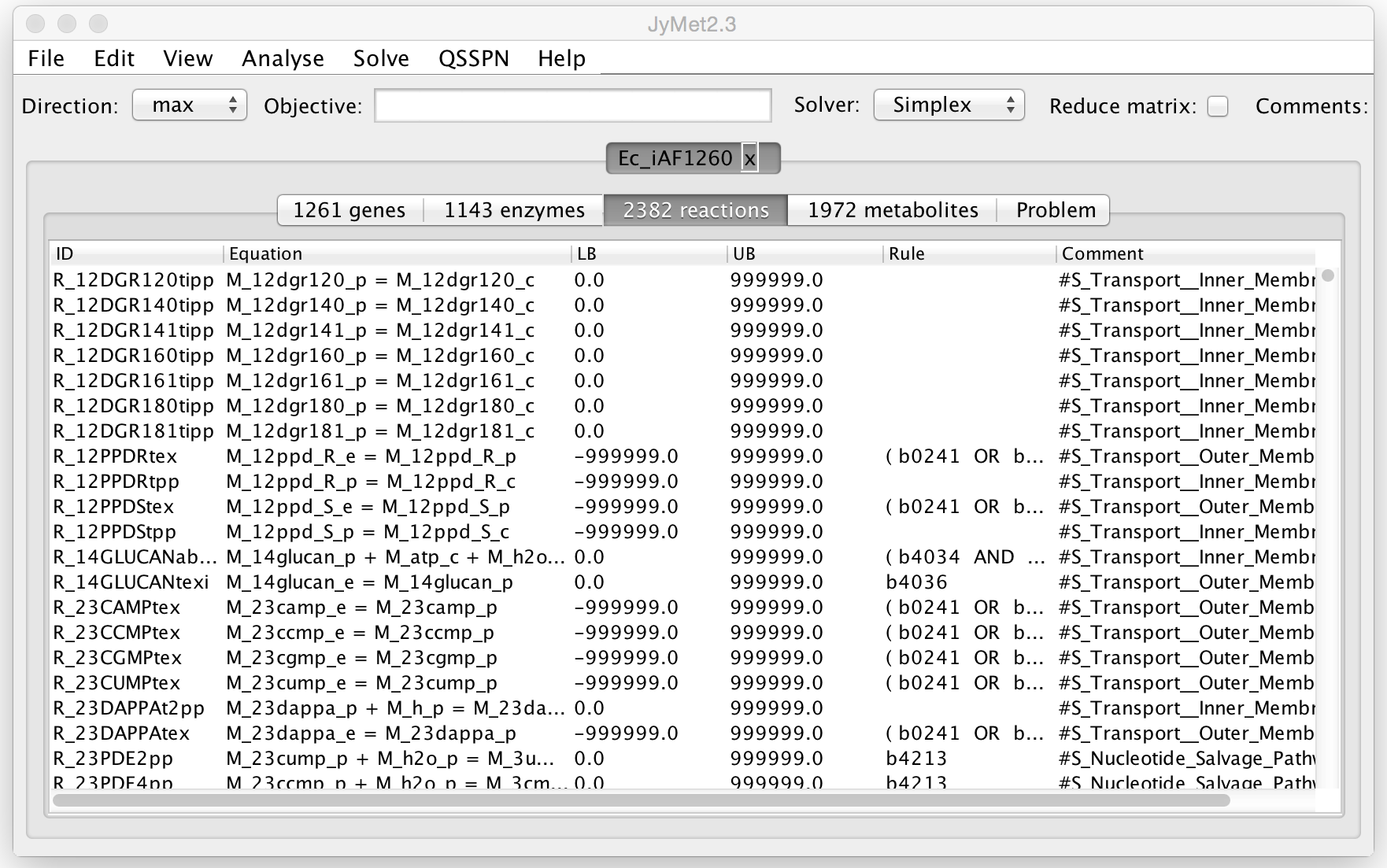
Figure 2: Import of Ec_iAF1260 SBML file, with reactions tab shown

### Saving models as a MUFINS reaction table, and editing with an external text editor.

Models within JyMet can be saved as a tab-separated text file using the ‘File🡺Save table’ command. This can be used to save a reconstruction in the MUFINS native format (sfba), or to save individual tabs, which can be useful to save reaction, problem and results tables for later examination.

To save the imported SBML file of the *E.coli* model in sfba format, make sure that reaction tab is active and use ‘File🡺Save table’ to create the file *Ec_iAF1260_flux1.sfba* file. Note that the tag has been changed from *.xml to *.sfba to denote that this is now a file used in the MUFINS.

As this file is saved as a tab-separated file, it can be opened with spread sheet programs such as Excel, or with a text editor. If this file is opened in Excel the you will see a warning stating that ‘The file you are trying to open is not a valid Excel XML Spreadsheet’ and asking if you wish to ‘Open as Text’, which you should accept. This is because the tag is *.sbml and not the expected *.xlsx or *.txt. Opening the file in Excel or a text editor means that this FBA model representation can be easily edited outside of JyMet, if desired. As long as the file is saved as a tab-separated text file then it can be loaded back into JyMet.

*Note:* Mac users may experience a problem when working with reaction tables saved from Excel. This is because Excel uses the Windows rather than Mac end of line format. To correct this problem, simply open and save the text file with another editor (e.g. TextEdit).

*Technical Note:* For information, the sfba and qsspn command line tools use this table as their native representation of the GSMN.

### Open a MUFINS sfba model in JyMet.

To confirm the successful saving of the file, we will now load the Ec_iAF1260 model in the MUFINS sfba format. In JyMet, use ‘File🡺Open model’ to open the Ec_iAF1260_flux1.sfba file. A
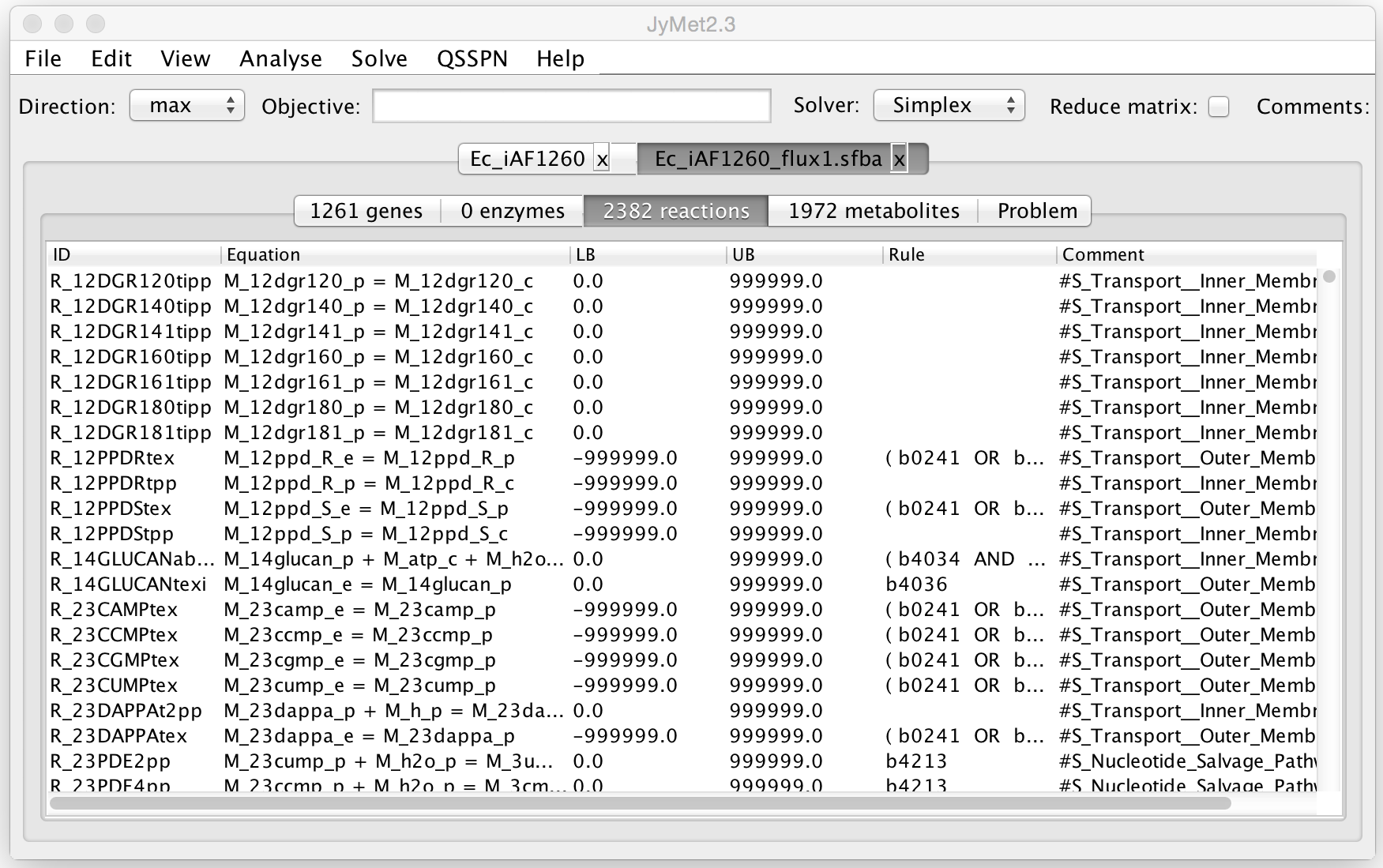
dialogue box will appear, warning that external metabolites are not defined. Click OK to acknowledge this message (it’s importance will be seen later on).

Figure 3: Import of the Ec_iAF1260 model in sfba format

As seen in figure 3, the imported sfba model looks very similar to the original sbml model import. However, clicking on the ‘enzymes’ tab will now show it has zero entries. As the sfba reaction table uses the gene-reaction association rules defined in the rule column, the enzymes tab is irrelevant and is discarded. We recommend users import/export models in SBML, allowing gene-enzyme-reaction associations to be defined, and using the universal markup language. However, model development and simulation the sfba format is sufficient and faster.

### Closing a model

JyMet allows users to work with multiple models at one time. Each model is represented as a separate tab at the top of the JyMet interface. For example, in figure 3, both the Ec_iAF1260 and Ec_iAF1260_flux1.sfba models are open. Clicking on either tab will select this model. To close a model, simply press the ‘x’ next to its name. Close the model Ec_iAF1260, leaving open the model in sfba format.

*Note:* JyMet does not ask you if you want to save any changes – once it is closed, any unsaved changes are lost. This is because in most instances you do not want to save changes to a model as they will be simulation-specific. However, please bear this in mind if you do not want to lose your hard work.

### Define the external metabolites for the GSMN

External metabolites represent sources and sinks of metabolic flux. Without external metabolites the only value for fluxes within the network will be 0, because there will be no source of metabolic flux. Before we undertake any analysis, it is necessary to tell the software which metabolites are external, which is indicated by a tag at the end of their names. For example, models originating from Palsson’s group usually use the ‘_b’ tag indicate an external metabolite. To define external metabolites click ‘Solve🡺Externality tag’ in JyMet menu and type ‘_b’ in the dialogue box (figure 4). If you are unsure whether the externality tag has been defined, this can be quickly checked by
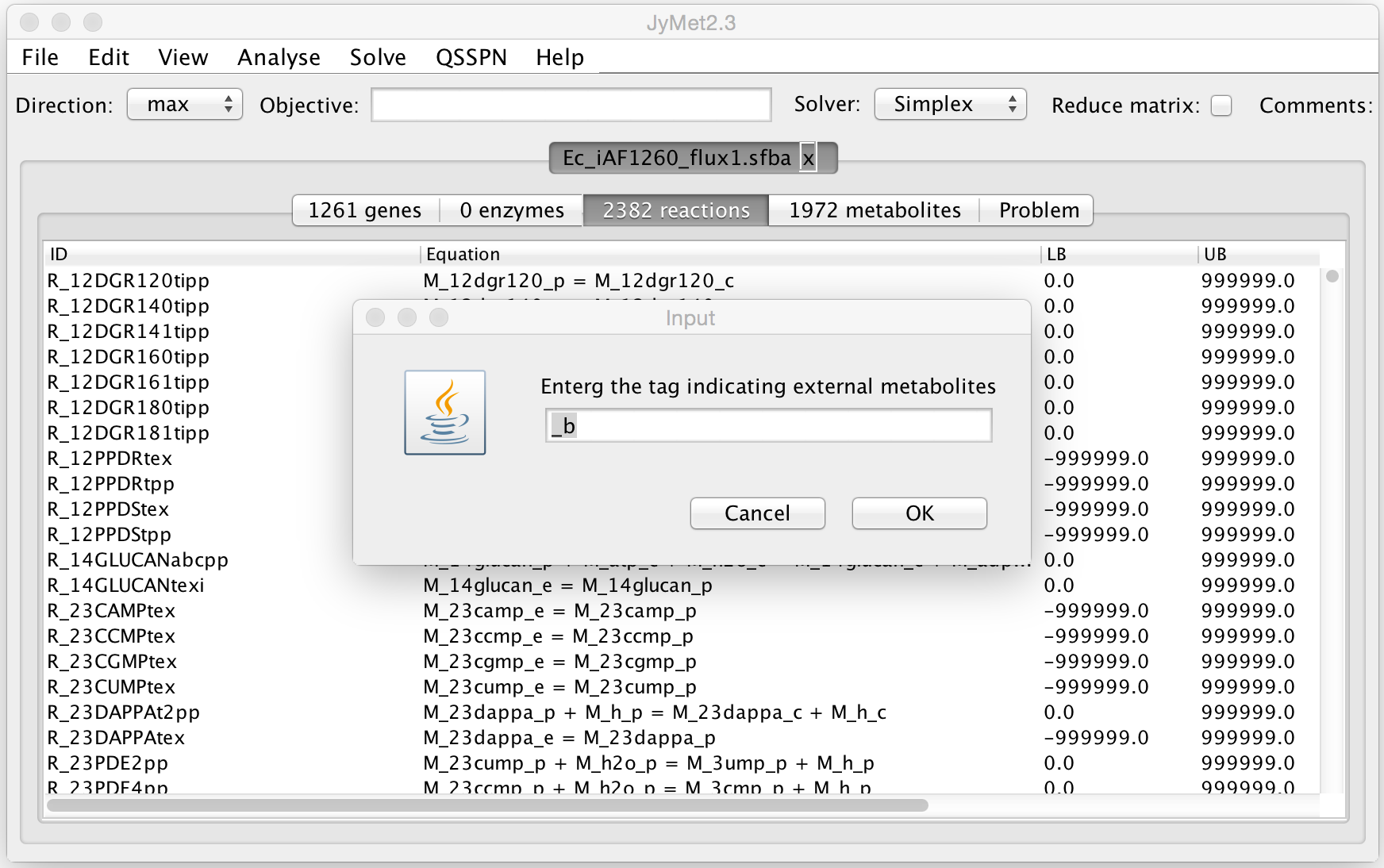
using ‘Solve🡺Externality tag’ – any previously set externality tag will be shown in the dialogue box.

Figure 4: Setting the external metabolite tag in JyMet

### The Biomass reaction and it’s relationship to growth rate.

As discussed in the introduction to this exercise, a common question in constraint based modelling is to predict the maximal growth rate possible, commonly referred to as biomass. In many metabolic networks, there is a reaction termed ‘biomass’ (or similar) that defines the biosynthethic and energy demand required for the synthesis of cell components. Therefore, the flux through this ‘biomass’ reaction represents the growth rate of the population of cells.

The biomass reaction in iAF1260 model has a long name that is difficult to remember. Therefore, it is easier to use the Search function of JyMet to find it using the ‘biomass’ substring. With the reaction tab visible, select ‘Edit🡺Search’ and enter ‘biomass’ as a query. The table will show any reaction that contains the substring ‘biomass’ anywhere in the reaction row. In this case, only one row will be returned (figure 5).

*Note:* If you do not find the biomass reaction by searching, this usually means that a single row was selected, and JyMet searched only this row. To avoid this issue (i) make sure that all reactions are shown (and can be searched) by using ‘View🡺Show🡺All’. (ii) Select all the rows (ctrl-A or
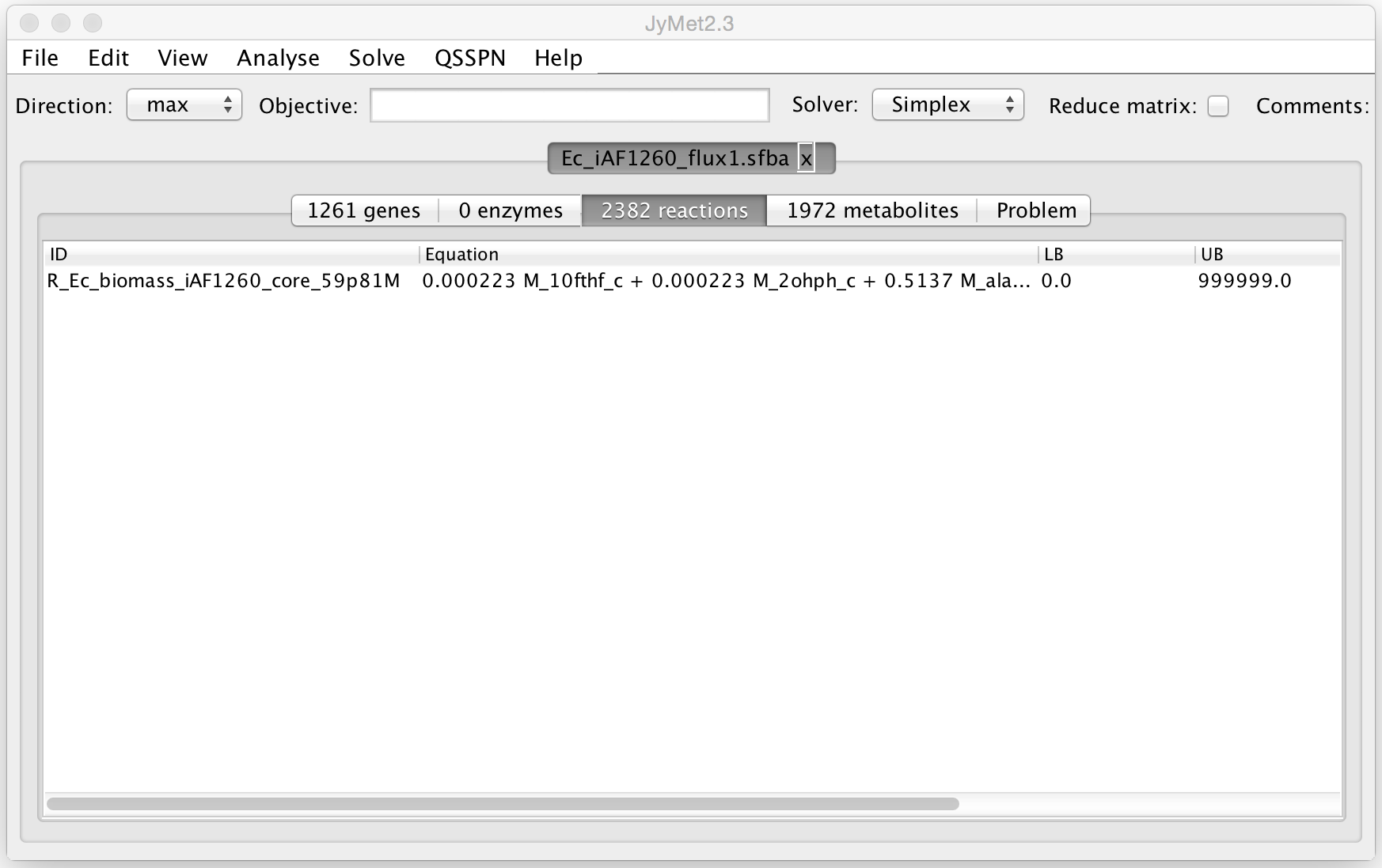
cmd-A), ensuring they will all be searched.

Figure 5: The biomass equation.

Clicking in the ‘Equation’ field allows you to scroll through the biomass definition. You will see that it contains metabolites that are considered to be required for biomass (e.g. nucleotides, amino acids *etc.*). Each metabolite is prefixed by a number, which represents the coefficients of these substances that reflect the experimentally determined biomass composition of the *E. coli* cells under specified medium conditions. Finally, to return to the all reactions view, select ‘View🡺Show🡺All’.

### Calculate the maximal growth rate

To run simulations with JyMet you must complete three tasks: (i) define the objective function, (ii) choose the analysis method, and (iii) write the problem file. Writing of the problem file makes execution of simple task a bit more complicated than necessary, but gives a lot of flexibility for definition of complex analysis protocols and media conditions.

Define the objective function: The objective function is a requirement for a number of different CBM approaches. At the simplest level, it defines what we wish to examine in the analysis. In this case, we are looking to see what is the maximal possible flux through the biomass reaction, analogous to the growth rate. The simplest way to set the objective function is to enter it into the ‘Objective:’ field at the top of the JyMet interface. You can also select here whether you wish to maximize or minimise the objective function, using the ‘Direction:’ drop down menu (Figure 6).


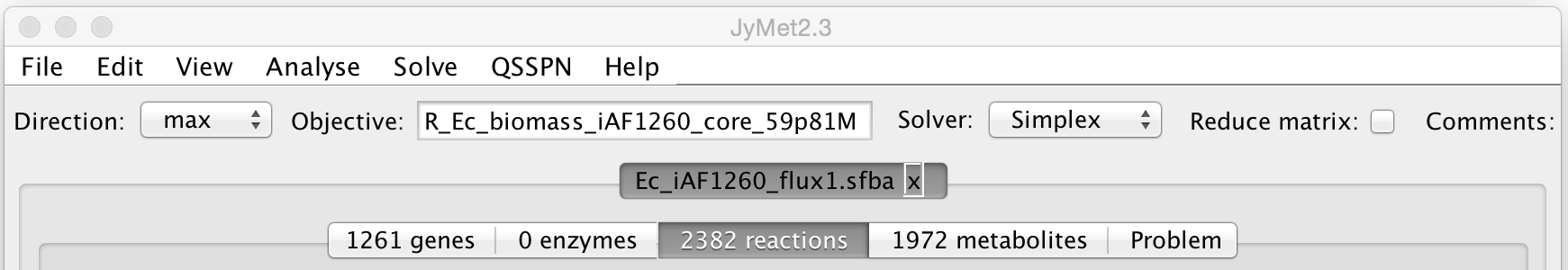


Figure 6: Setting the objective function

Choose the analysis method: The ‘Analyze’ menu contains a selection of approaches useful in CBM. For now, we will look at on of the simplest analysis, where just the value of the objective function is returned. To choose this analysis mode, select ‘Analyze🡺Objective value’. This is the default opion in MUFINS, but it is good practice to always check that you are using the desired analysis method.

Write the problem file: The problem file records what parameters are being used for the current simulation. To create the problem file, select ‘Solve🡺Write problem’. JyMet will switch to the ‘Problem’ tab, showing the currently defined problem (Figure 7). The expression in problem file window instructs the software to calculate maximal theoretical flux through reaction R_Ec_biomass_iAF1260_core_59p81M. The ‘;’ on the second line is to set the end of the problem. You can have several objective functions in one problem file, each separated by a line containing ‘;’, and MUFINS will solve each in turn. In addition, as we will see later, it is possible to add in many more instructions into a problem, further refining the
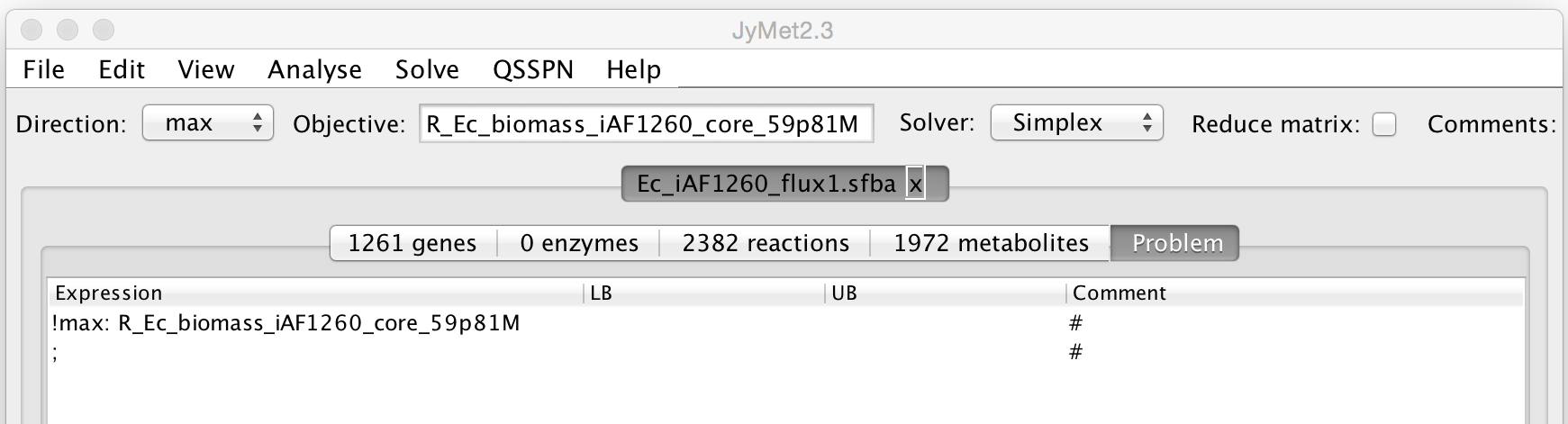
simulation to be undertaken.

Figure 7: Defining the objective function

Once we have set up the parameters for the simulation, we can instruct MUFINS to solve the problem by selecting ‘Solve🡺Solve’

As can be seen from figure 8, the maximal growth rate chemically feasible under the medium conditions of this experiment is 0.736701 h^-1^. The units of the flux in the original model are mmol. h^-1^.gDW^-1^ (milimole per hour per gram dry weight). As the coefficients in the biomass reaction are expressed in units of mmol.gDW^-1^, these are summed to produce the mole fraction of each precursor necessary to produce 1 g dry weight of cells. Therefore, biomass flux is calculated in units of h^-1^, and represents the growth rate of *E.*
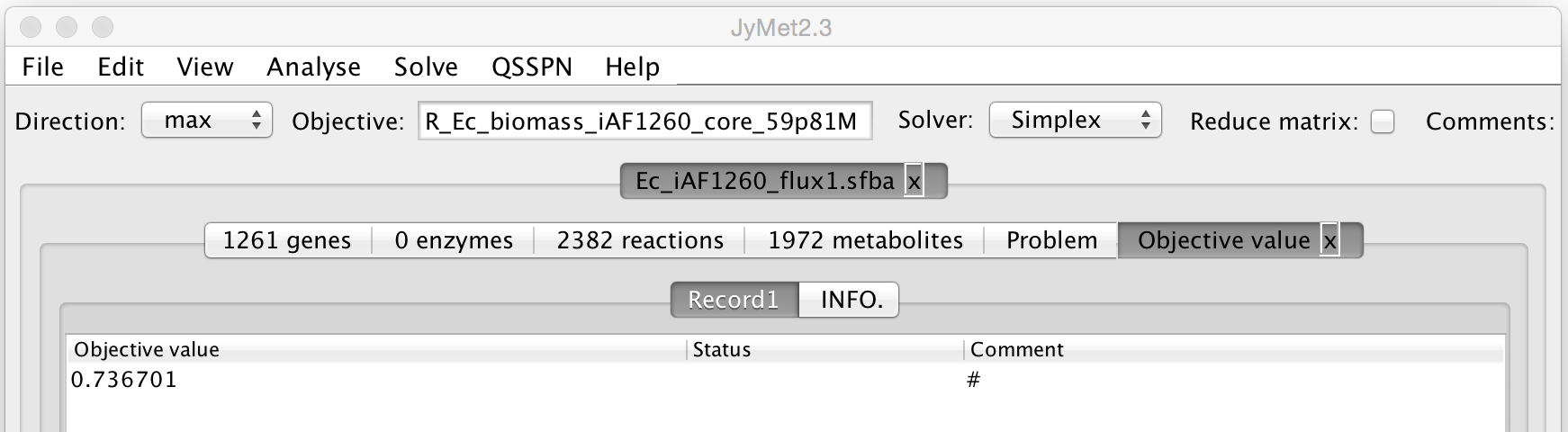
*coli* culture in the log phase.

Figure 8: Results of an Objective Value simulation

### Simulate the effect of different media conditions on growth rate

It is often interesting to examine the impact of different external metabolites on growth rate. In the Ec_iAF1260 model, the carbon source available in the external environment is glucose. The reaction
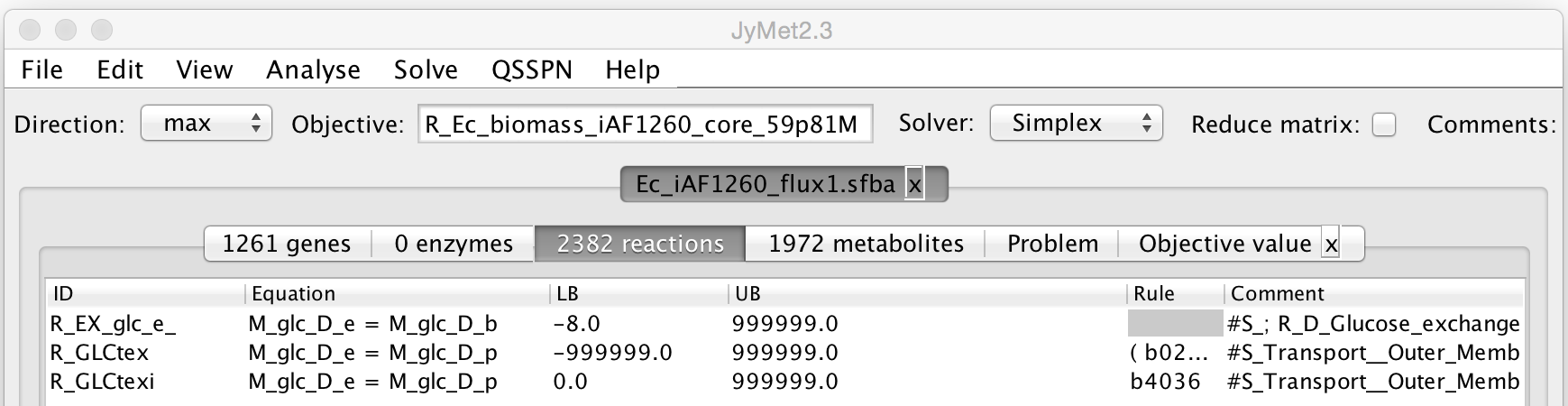
representing glucose exchange with the external environment is R_EX_glc_e_.

Figure 9: Glucose exchange reactions in Ec_iAF1260

As shown in figure 9, reaction R_EX_glc_e_ describes the transport of glucose between the extracellular space (M_glc_D_e) and the external environment (M_glc_D_b). The LB and UB are both open, showing that this reaction is bidirectional. However, while the UB (secretion into the external environment) is fully open, the LB representing glucose uptake from the external environment is limited. The LB maximal flux is set to -8.0, meaning the reaction will be capable of transporting 8 mM of extracellular glucose (M_glc_D_b) into the cell (becoming M_glc_D_e) in one hour per 1 g of cellular dry weight.

Two other reactions are shown in figure 9, R_GLCtex and R_GLCtexi. These two reactions represent the transport between the extracellular space and periplasm of the bacterial cell. R_GLCtex is a bidirectional reaction, with LB and UB allowing flux, while R_GLCtexi is a unidirectional transporter into the periplasm.

Altering uptake of glucose from the external environment: To examine the impact of altered glucose availability, we need to open or close some of the exchange reactions by changing their flux bounds. This can be done by directly altering the LB and UB in the model. However, this is not best practice, as it is easy to forget what changes you have made. It is better to set new reaction bounds in problem file. To edit the the problem file, click on its tab to make it visible. Next, use ‘Edit🡺Insert rows’ to add a row to the problem file table. To create a rule, setting the new bounds, enter ‘R_Ex_glc_e_’ in the expression column, and then LB and UB values of 0.0 and 999999.0, respectively. This will set the R_EX_glc_e_ to secrete only, preventing the cell taking up glucose from the external environment. Make sure to place a ‘;’ in the last row, showing that this is the end
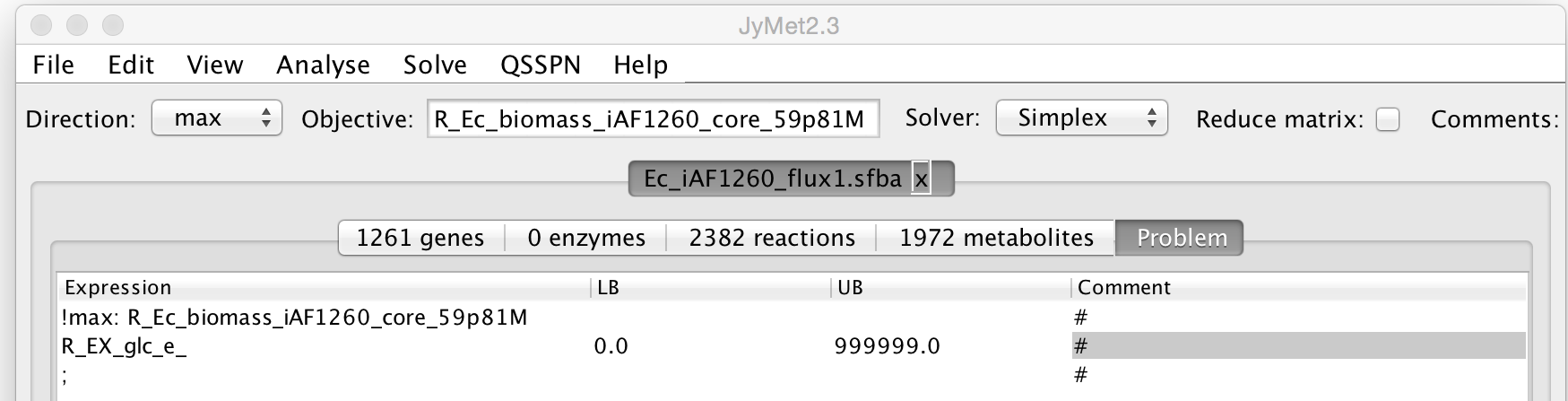
of the problem definition (figure 10).

Figure 10: Altering reaction bounds using the problem file.

Any reaction bounds specified in a problem file will automatically **overwrite** the settings in the reaction table. To show the effect of preventing the bacteria using glucose on the growth rate, calculate the maximal growth rate again (Solve🡺Solve). Since uptake of the sole carbon source is closed, the maximal growth rate should be 0. Set the R_EX_glc_e_ reaction bounds back to original settings, and confirm that the maximal growth rate reverts back to 0.736701 h^-1^. This will show that you can now control reaction bounds using settings in the problem file.


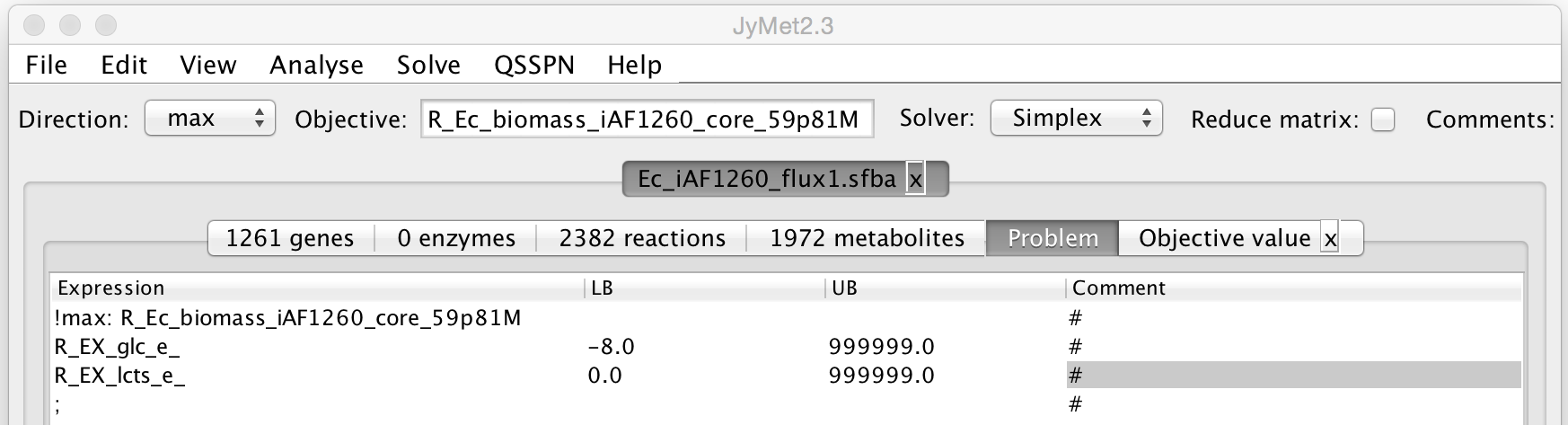
Addition of a second carbon source - lactose transport: As *E.coli* can utilise several different carbon sources, it may be of interest to predict the impact on growth rate. Lactose exchange is defined by the reaction R_EX_lcts_e_. To enable control of the available carbon source between glucose and lactose, add a row to problem file table (Edit🡺Insert rows) and add the lactose exchange reaction (Figure 11)

Figure 11: Addition of the lactose exchange reaction to the problem file

Now you can control which carbon source is available by changing the LB of these two reactions. With a LB of -8.0 for glucose uptake only, you should see a growth rate of 0.736701 h^-1^. If you switch to lactose (LB -8.0) as the carbon source, the growth rate increase to 1.082 h^-1^. Finally, if both carbon sources are available, the growth rate increases again to 1.31467 h^-1^.

## Exercise 2. Essential reactions.

An important application of computational modelling is the identification of metabolic chokepoints, critical reactions that are essential for meeting the objective function. MUFINS allows you to search for these essential reactions by sequentially constraining the flux through each reaction to 0 and calculating the maximal value of the objective function. If inactivation of a reaction results in an objective value equal to 0, then this reaction is reported as essential. In other words, the software reports any reactions that have to be active to make certain metabolic objectives (e.g. growth) chemically feasible.

In this example, we will search for essential reactions for growth of *E.coli* on a minimal medium that contains only glucose as a carbon source. If you are continuing straight on from Exercise 1, you need to reset the problem file. This would be a good time to practice the skills you learnt in Exercise 1, and open the files from scratch.

In JyMet, use ‘File🡺Open model’ to open Ec_iAF1260_flux1.sfba file. When the dialogue box appears, warning that external metabolites are not defined, click OK. Next, set the externality tag using ‘Solve🡺Externality tag’ from the JyMet menu and type ‘_b’ in the dialogue box. Finally, set define the objective function: Enter the biomass equation ID into the ‘Objective:’ box (R_Ec_biomass_iAF1260_core_59p81M). The write the problem file using ‘Solve🡺Write problem’.

Once we have set up the problem file, we can begin the analysis. First, set the analysis method to essential reactions using ‘Analysis🡺Essential reactions’. Next, start the analysis by selecting ‘Solve🡺Solve’. Depending on the computer hardware you are using, the analysis should take between two and ten minutes. Once the analysis is complete, you will see the screenshot shown in figure 12.


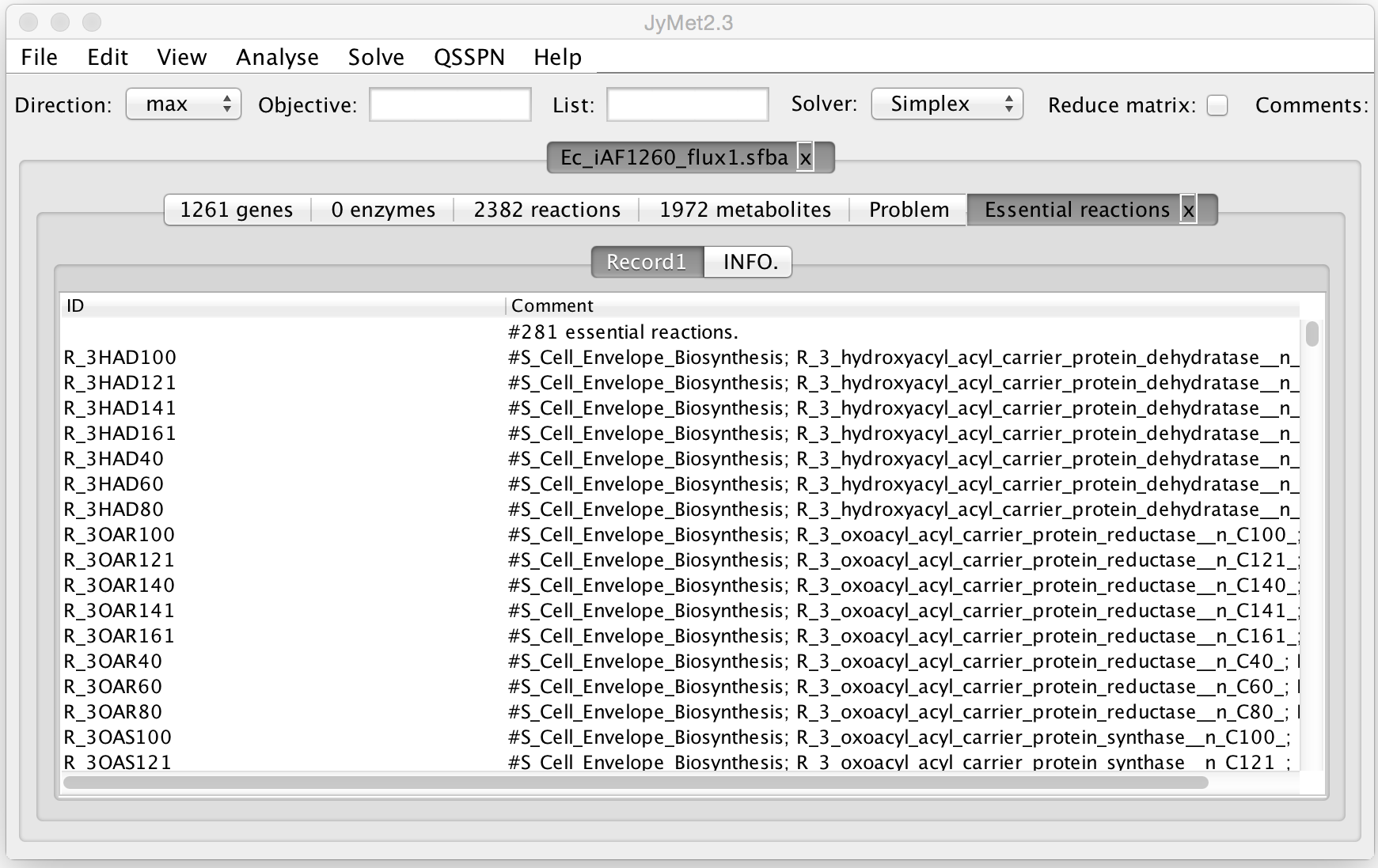
Figure 12: Output from a Essential Reaction Analysis

The first row of the results table tells you that 281 reactions (out of 2382) are essential. The rest of the results table is divided into two columns. The first column provides the reaction ID. The second column shows the comment section for each reaction, which describes the reaction mechanism. As before, the results table can be saved using ‘File🡺Save table’: we recommend the ‘.txt’ tag, allowing the file to be opened in both text editors and spread sheet programs such as Excel.

## Exercise 3. Using the visualisation tools within JyMet

JyMet, the graphical front end for MUFINS contains a visualisation tool to provide a rapid, intuitive tool for interrogation of simulation results and help optimise the model development pipeline. In this exercise, we will use a model of cell signalling, gene regulation and whole-cell metabolism in the RAW264.7 macrophage. This model is described as Use Case 1 in the original MUFINS publication, with a full description as Supplementary Information. The RAW264.7 mouse macrophage genome-scale metabolic network was first published by Bordbar *et al* (3).

### Open the model and prepare it for simulation.

The folder ‘MUFINS🡺Examples🡺RAW_264_7_r’ contains two files: an sfba file describing the model, and a pfile defining the problem. Open the sfba file in JyMet using ‘File🡺Open model’. When the dialogue box appears, warning that external metabolites are not defined, click OK. Next, set the externality tag using ‘Solve🡺Externality tag’ from the JyMet menu and type ‘_xt’ in the dialogue box.

As can be seen in Figure 13, once the model opens it contains and usual reaction command: ~. This represents the linear inhibition constraint, a unique feature in MUFINS that sets an inhibitory command. For example, reaction r3 has the equation ‘~akt1 = gsk3b’; this is read that akt1 inhibits gsk3b. The command ‘&’ can be similarly used to indicate a stimulatory interaction.


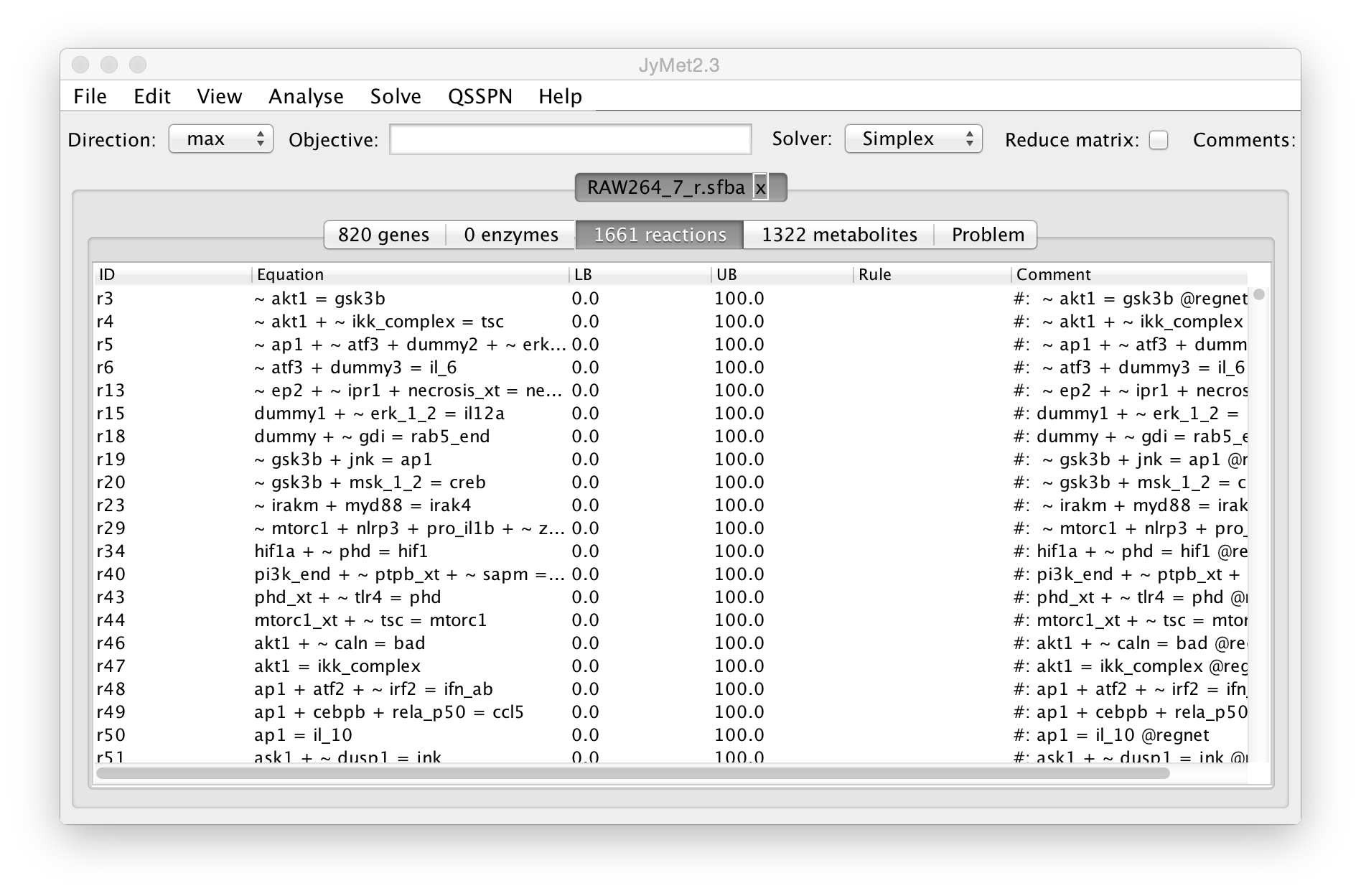
Figure 13: A regulatory network in MUFINS using linear inhibitory constraints

All the reactions in regulatory part of the model are ‘tagged’ with ‘@regnet’ in comments section. As ‘#’ symbol separates comment part of the line, the ‘hashtag’ cannot be used. Here we use @regnet as it is a unique tag for ‘Edit🡺Search’ function. Combination of multiple tags can also be used to enhance the granularity of model description.

### Open the problem file

Use ‘File🡺Open problem’ to load *RAW264_7_r/simulate.pfile*. This file defines a series of problems, with each simulation separated by a row containing ‘;’. These *in silico* experiments investigate the influence of LPS and a Mek1 inhibitor on the maximal rate nitric oxide production. A
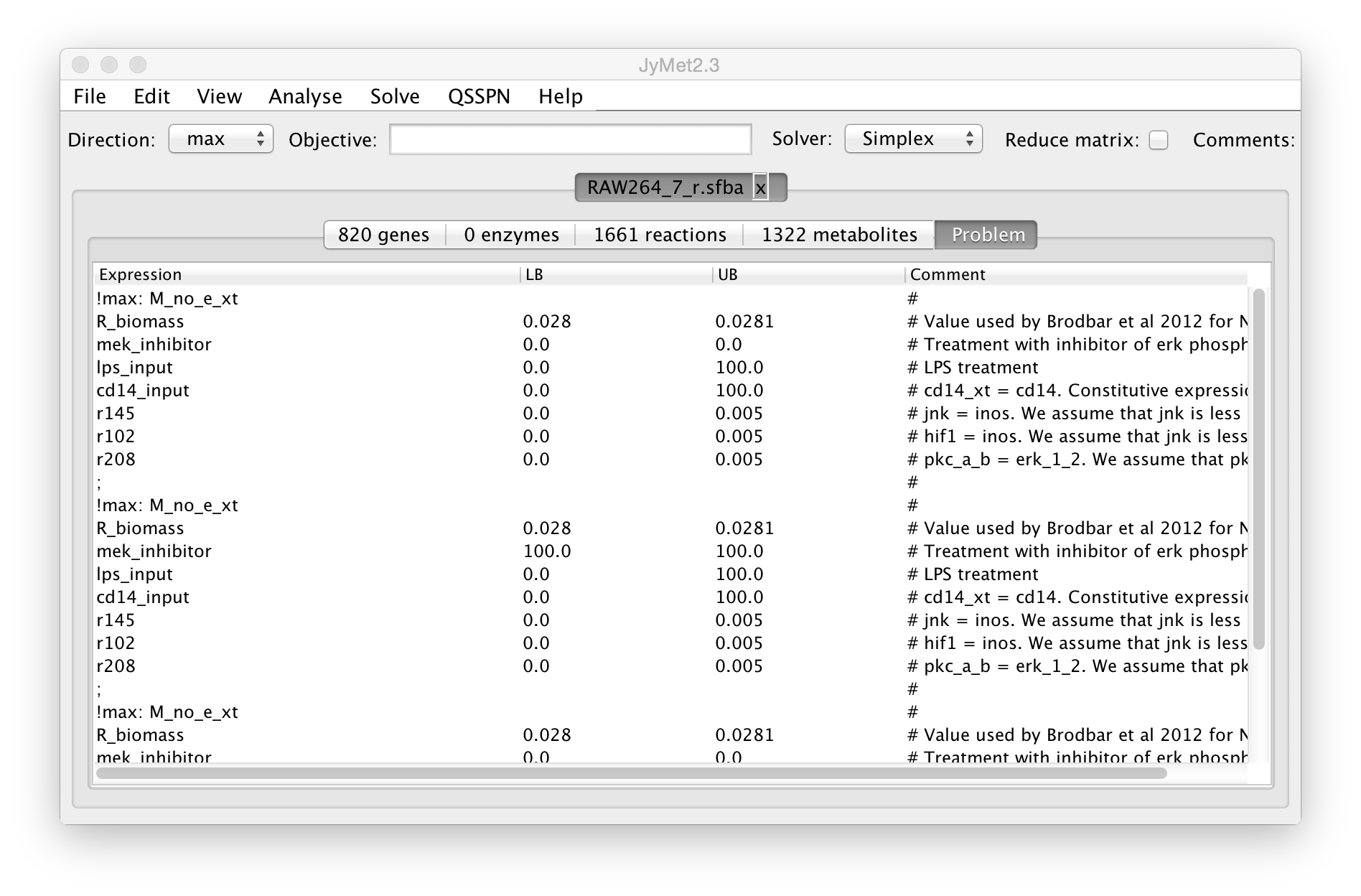
section of the problem file can be seen in figure 14.

Figure 14: Example problem file with multiple simulations

For each problem, the objective function is set maximize production of nitric oxide in the external environment (!max: M_no_e_xt). The following lines are used to set the parameters for each simulation. These include the required growth rate (R_biomass), the levels of the MEK1 inhibitor (mek_inhibitor) and LPS (lps_input), plus the maximal flux rate for three reactions in the regulatory network (r145, r102 and r208).

Looking at the bound values for mek_inhibitor (Figure 14), you can see that the difference between the first and second simulation is the absence and presence, respectively, of the MEK1 inhibitor.

### Run Flux Balance Analysis

Having set up the model and defined the problem, we can now undertake the analysis. First, select Flux Balance Analysis as our simulation mode (‘Analyze🡺FBA’). Next, we can run the simulation using ‘Solve🡺Solve’. Once the simulation has completed, the results are presented as a table (Figure 15), with each simulation in the problem file presented as a separate tab (Record1-3). In addition, the ‘INFO’ tag contains the simulation log, including the command lines used to run the simulation tool. These command lines can be used as examples to run stand-alone simulations or scripts in the command line, if desired. The results table for Record1 contains the maximal objective function value
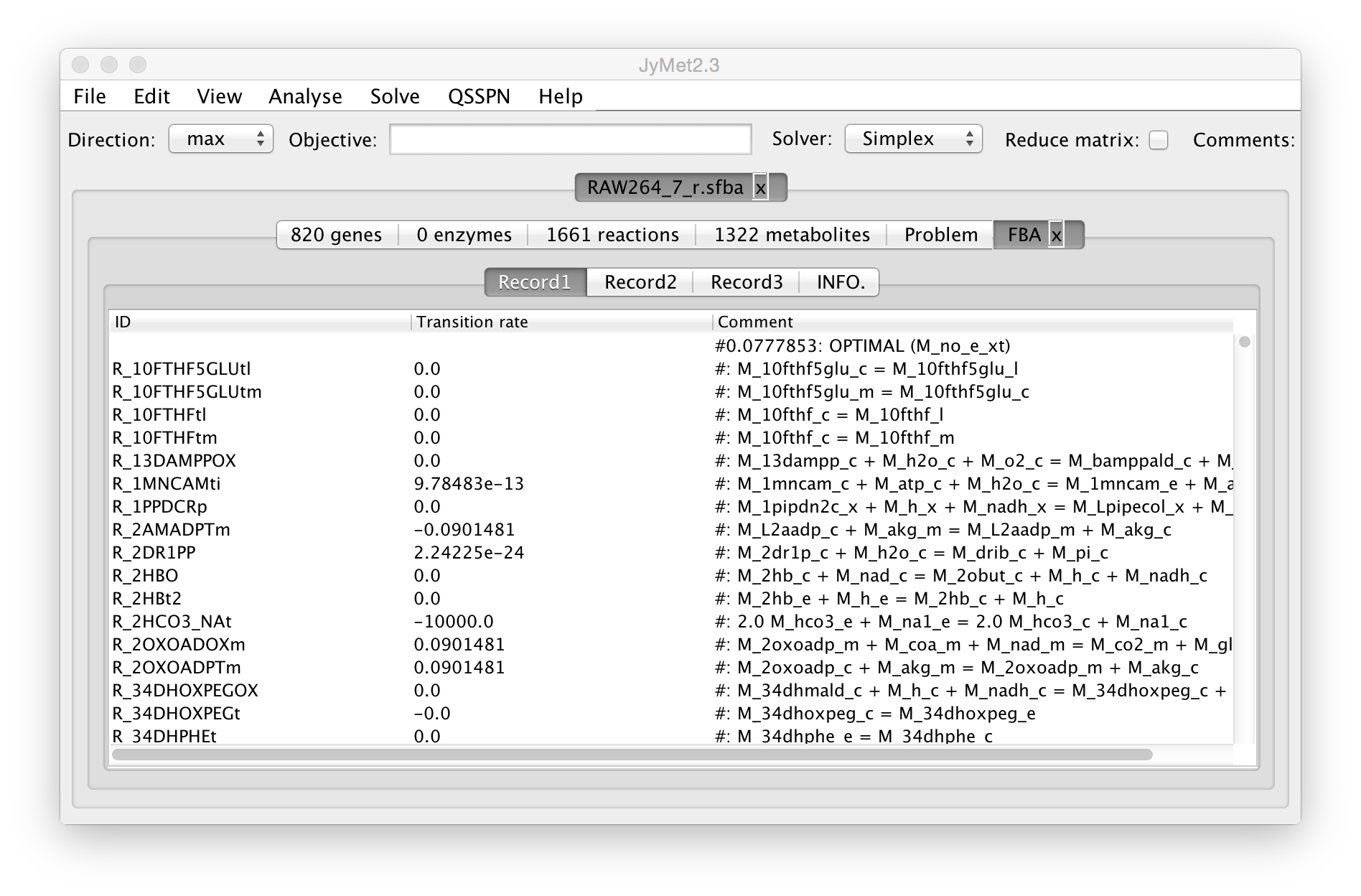
(0.0777853 h^-1^), and an example flux distribution.

Figure 15: Results table from a Flux Balance Analysis

### Preparing the results table for visualisation

While it is theoretically possible to visualise the entire network, this would just produce a ‘hairball’ that would be too complex to be of much use. Therefore, before a visualisation is produced, it is usual to reduce the number of reactions that will be viewed to a manageable number. This can easily be achieved through two steps: (i) selection of the sub-network of interest, and (ii) selection of only reaction with non-zero flux values.

Selection of the sub-network of interest: In this exercise, we wish to visualize the signalling network. A good practice during model reconstruction is to add comments that allow easy identification of key sections of the final reconstruction. In this case, all regulatory reactions are tagged with the string ‘@regnet’, allowing their quick identification. To find all reaction associated with regulation in the results table, use ‘Edit🡺Search’ to filter all reactions tagged ‘@regnet’.

Selection of reactions with non-zero flux values: As we are interesting in what is happening in the network, we can effectively ignore reactions where the flux is zero. In common with many spread sheet-style outputs, the results table can be sorted by clicking on the column headings. By clicking on the column header ‘Transition rate’ reactions will be sorted in order of flux value (highest to lowest). It is then simple to select all regulatory network reactions with non-zero flux in this particular example solution.

The resultant, sorted results table is shown in Figure 16


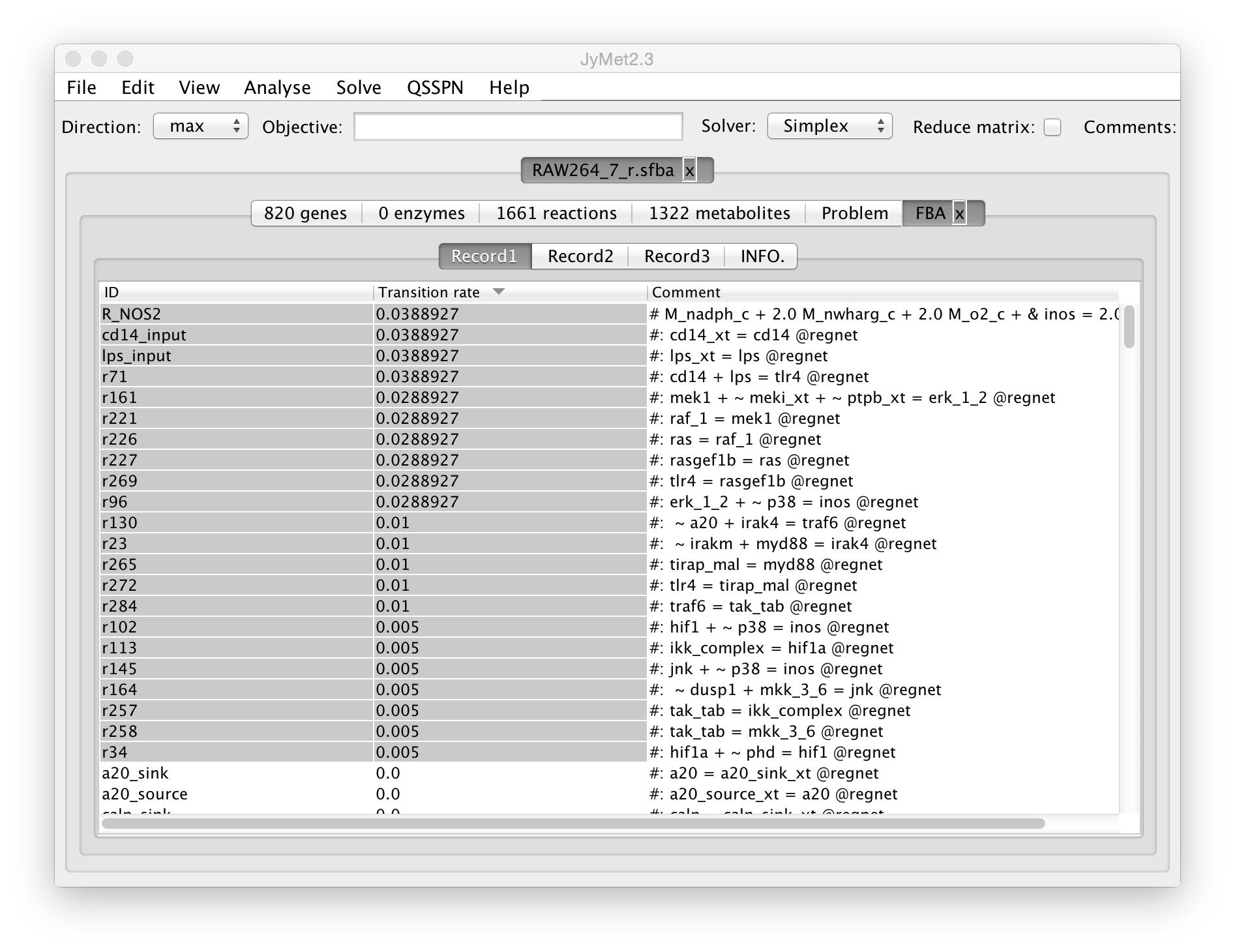
Figure 16: Results table with only non-zero flux regulatory reactions selected

### Generating a visualisation in JyMet

Once the results table has been prepared, selecting only those reactions that you wish to include in the visualisation, it is possible to generate the layout. To create an automatic layout, the options within ‘View🡺Layout’ are used. There are several different layout options available, but for the purpose of this exercise select ‘View🡺Layout🡺Hierarchical’: the resultant visualisation is shown in Figure 17. The network is visualised in Petri Net (bipartite graph) notation with rectangles representing transitions and circles representing places. Flux values are written within each reaction rectangle, with the flux values for each component of a reaction indicated next to the reaction arrow. Line thickness is also used to visualise fluxes.


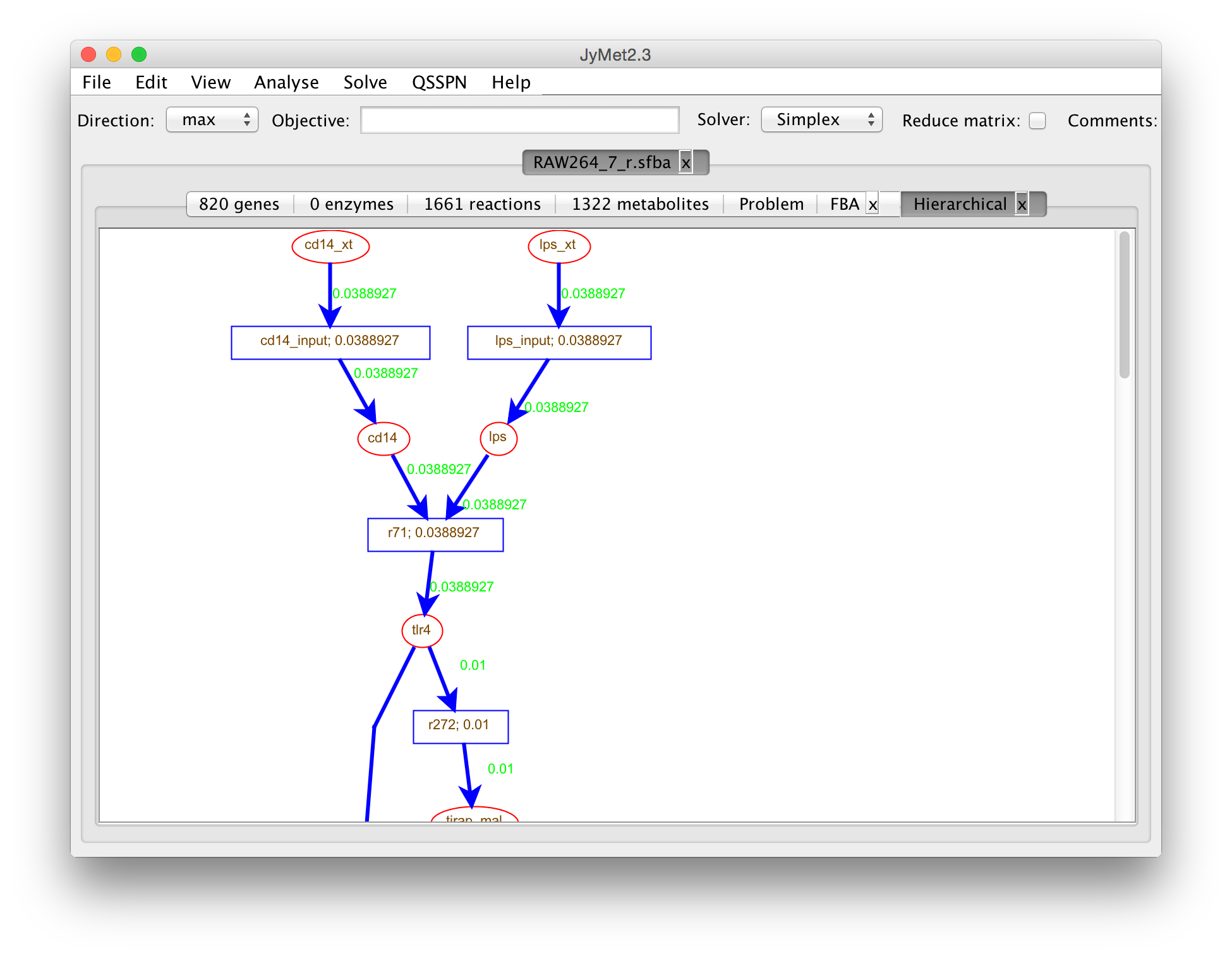
Figure 17: Visualisation of an example FBA solution

It is possible to scroll around the visualisation, and right-clicking brings up a menu to allow zooming in and out. The layout is fully manually adjustable by clicking on any component to select it. Visualisations can be saved for later use with the ‘File🡺Save graph’ function. This saved graph can then be reopened at a later time in JyMet, by clicking ‘View🡺Layout🡺Custom’.

We note that the visualisations generated in JyMet are not intended to produce publication-quality images. Rather, they provide a tool for quick, iterative examination of simulation results, helping to streamline model reconstruction and interrogation. To generate publication-quality images we recommend using the JyMet-generated visualisation as a design template, creating the final image in dedicated desktp publishing software such as MS PowerPoint or Adobe Illustrator. Figure 18 demonstrates this process.


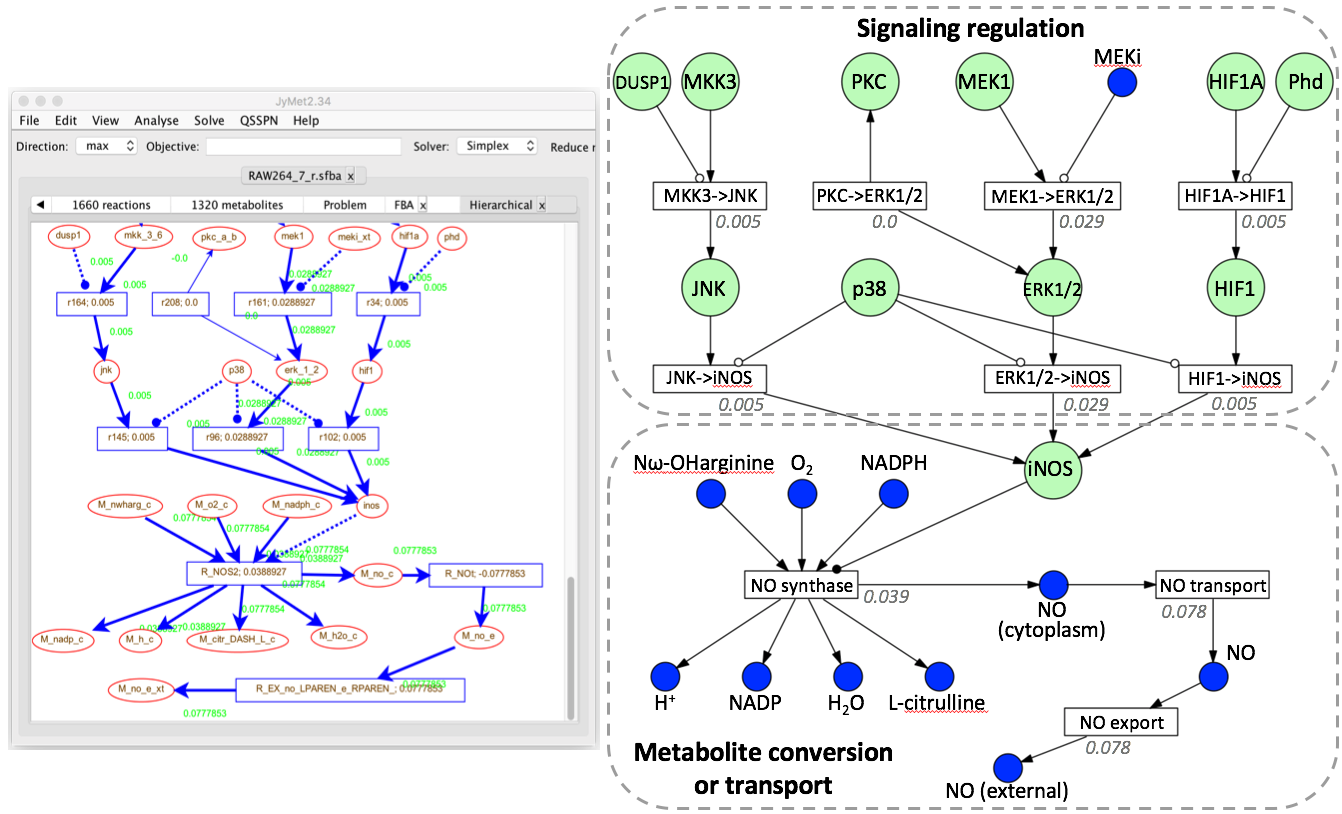


Figure 18: Use of a JyMet-generated visualisation as a template for publication-quality images

# Quasi Steady State Petri Net (QSSPN)

The QSSPN algorithm integrates Petri nets and flux balance analysis to perform qualitative dynamic simulation of molecular interaction networks involving gene regulation, signaling and whole cell metabolism (4). Simulation is performed by a hybrid simulation algorithm based on the maximal timestep method (5), making it numerically stable and facilitating interrogation of modular models reconstructed at varying levels of detail. As shown in figure 19, the Petri net is used to describe the regulatory aspects of the model, such as ligand activation of a receptor, or gene expression. Changes in expression levels/activation status of metabolic proteins are transferred to the GSMN by setting the LB and UB for mapped reactions. Importantly, reaction flux values can be extracted from the FBA solution for a given objective function, and used to feedback into the regulatory network, meaning that a fully dynamic simulation can be achieved. The Petri net is also used to set further constraints on the simulation, for example setting a biomass flux value constraint to represent cell turnover.


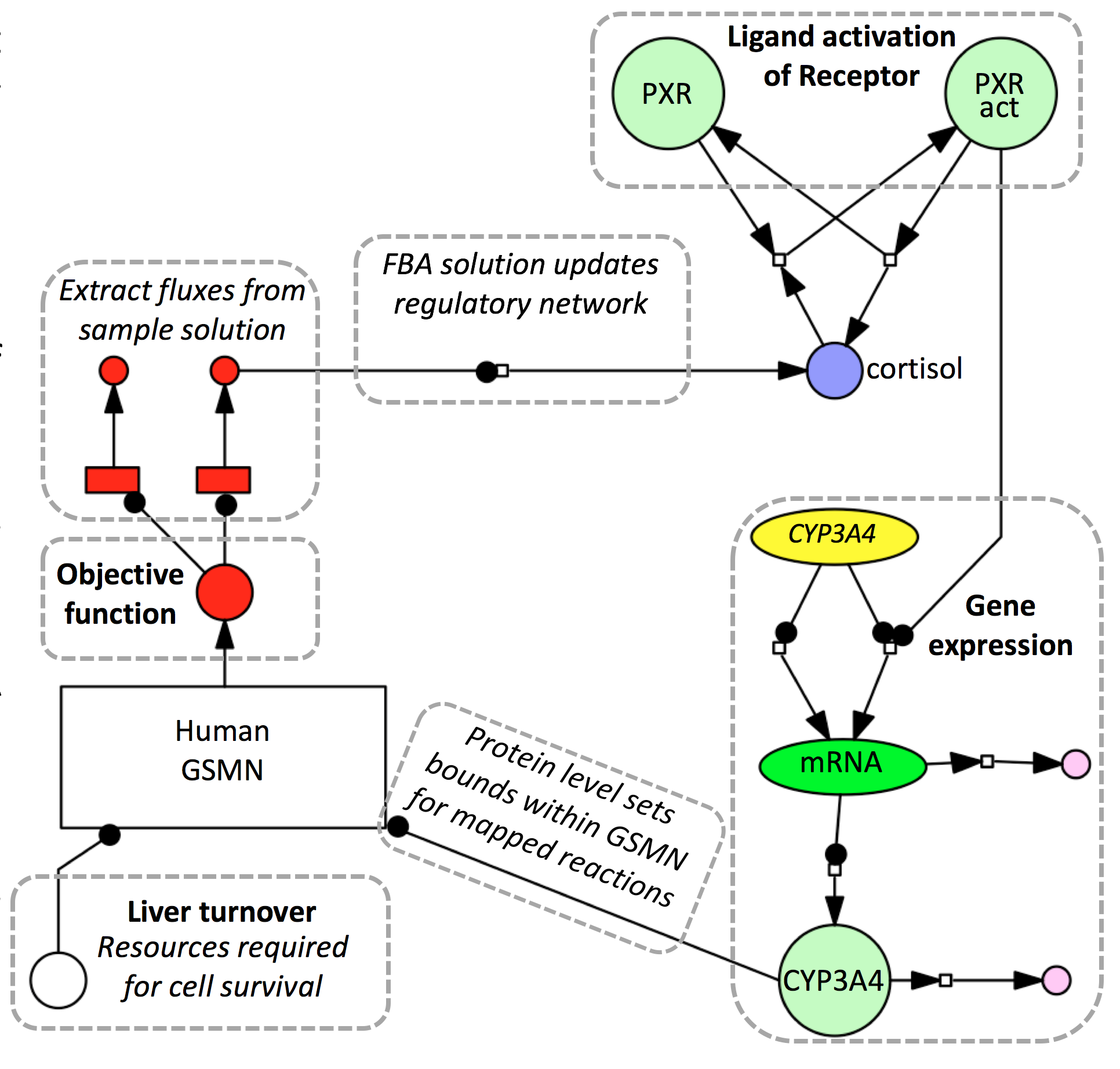
Figure 19: Overview of QSSPN approach

This tutorial introduces QSSPN model building in the Snoopy Petri Net editor (6) and subsequent simulation in MUFINS. We will use a kinetic model of cortisol signaling integrated with a dFBA simulation of human Genome Scale Metabolic Network (GSMN). The cortisol signaling model is a simplified version of the model presented by Kolodkin et al. (7), while the GSMN is the community based reconstruction Recon2 (8).

This model involves all the new features of QSSPN available in MUFINS. Specifically, the Petri net now represents a fully parameterized Ordinary Differential Equation (ODE) model of cortisol signaling and external metabolite concentrations in physiological compartments. To learn how to use QSSPN for non-parametric simulation with Monte Carlo sampling of qualitative dynamic trajectories, please see the original QSSPN publication (4). All features of original QSSPN method are retained in new version.

## Exercise 1: QSSPN simulation of cortisol signaling in the liver

### File Structure for a QSSPN simulation

QSSPN is probably the most complex simulation undertaken with MUFINS, and it is worth taking some time to explore all the files required. Examples of the ‘user’ files required for a simulation are contained within the folder ‘MUFINS_Examples🡺R_Recon2’. The simulation-specific files are contained withinin the subfolder ‘MUFINS_Examples🡺R_Recon2🡺bin’.

#### User-specific files to run a QSSPN simulation

NR_Recon2.v3.1.spept – This is a Snoopy output file. Containing the model of cortisol signaling integrated with a dFBA simulation of the human Genome Scale Metabolic Network (GSMN). This Snoopy file is used to build Dynamic Transition (DT) part of the model. The constraint and objective places that connecting the DT and QSSF parts of the model are also defined within the Snoopy file.

*Note:*  The latest version of Snoopy saves in the format *.xpn rather than *.spept. However, MUFINS will read both versions.

recon2_xt.PIPES.CORE.v1.sfba – The MUFINS reaction table file describing the human GSMN Recon 2 [3]. This file is used to build the Quasi Steady State Fluxes (QSSF) part of the model. The Recon2 reconstruction has been further paramaterised to constrain the solution spave further. Specifically. only exchange reactions (those that link the GSMN with the external environment) present in the HepatpNet1 PIPES are set to allow uptake from the external medium. PIPES represents those metabolites present under normal physiological conditions (9). In addition, the maximal uptake flux values are also constrained based upon experimental metabolite consumption and production data (10), further constraining the solution space.

NR_Recon2.v3.1.ctrl – This is the control file that sets the parameters for the QSSPN simulation. The control file can contain the following commands, with an example control fils shown in figure 20:

MODEL: this defines the model to be simulated. As this is placed in the same folder as the control file, it’s location is designated by ./name.sfba

NUMBER_OF_SAMPLES: Sets the number of times that a simulation is run (Default=1)

SEED: Sets the seed for the random number generator used during stochastic transitions

TIME_MAX: simulation time

MAXIMAL_TIMESTEP: Sets the timestep for the simulation

MAX_CHANGE: Allows an adaptive timestep to be introduced if necessary. A value or 0.01 indicating a 1% change in token status of a palace

OUTPUT: Specifies the file that stimulation results are written to. Usually an Excel file within the current directory (e.g. ./name.xls)

LOG: Specifies the file for any log entries to be written to. Usually a txt file within the current directory (e.g. ./name.txt)

MONITOR: Sets the number of iterations between writing of data to the output file. This parameter can be altered to achieve sufficient granularity in the simulation output

INITIAL STATE: Sets the token state for places within the Petri net

PROPENSITY_FUNCTION: Allows algebraic formulae to be used to define the transition propensity. Must finish with END

RESET_FUNCTION: Sets the value of the post-place based upon the pre-place (e.g. to be exactly the pre-place value, or 1/20 *etc.*). Must finish with END.

PETRI_NET_MONITORS: sets which places have their token status written to the output file

/* … */: Used to enclose comments

*NOTE:* values in the control file will **overwrite**
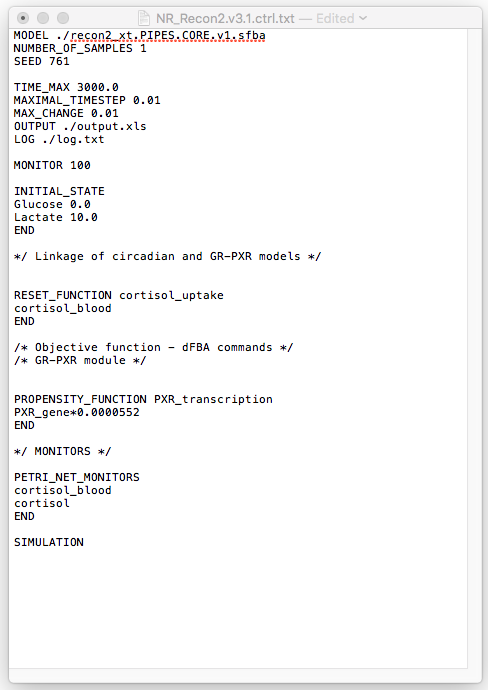
 any values entered into places or transitions within the Snoopy file. For best practice, we recommend that simple (i.e. mass action kinetics) rates be placed within the Snoopy file, while complex (e.g. Michaelis-Menten kinetics) rates be placed within the control file. All initial values that are not default (i.e. 1 or 0) are best placed within the control file.

Figure 20: Example control file for QSSPN simulation

In addition to these three obligatory files, the top directory contains two additional files. These files are not required to run a simulation, and merely automate the task for ease.

RUN_Mac – script that runs a QSSPN simulation of this example on MacOSX using binaries in bin/

CLEAN_Mac – scripts that clears simulation results on MacOSX.

#### MUFINS-specific files to run a QSSPN simulation

/bin/qsspn – *qsspn* solver for Mac copied here to create stand alone example distribution. The solver can be run in command line mode or started from JyMet interface or other scripts.

/bin/qsspn.exe - windows binary of *qsspn* solver

/bin/qsspn-linux – linux binary of *qsspn* solver.

*Note:* If this binary does not work on your version of Unix go to MUFINS**_Source/QSSPN/ and run compile.sh, install.sh to create a new binary in MUFINS**_Source/QSSPN/bin.

/bin/spept2qsspn – python script converting Snoopy *.spept or *.xpn files to a QSSPN model file. This script provides the ‘semantics’ for a Petri net model build in Snoopy. It interprets the graphical symbols and annotations in the comments section to construct the Petri net part of the QSSPN model to be executed with the *qsspn* solver.

### Use of Snoopy to create Petri net representations of signaling networks

To create a regulatory network in Petri net formalism, we recommend the use of Snoopy, a graphical editor for Petri nets (6). QSSPN models can also be edited using the spreadsheet interface of JyMet, or through spreadsheet programs such as Excel, or a text editor. However, we recommend editing models in Snoopy to take full advantage of Petri Net graphical notation.

Software distributions of Snoopy for multiple platforms are available at:

http://www-dssz.informatik.tu-cottbus.de/DSSZ/Software/Snoopy#downloads

The graphical user interface of Snoopy makes model building intuitive. Figure 21 shows the Snoopy model to be used in this exercise, with additional annotation to aid understanding. To see the naïve model (without annotation), open ‘NR_Recon2.v3.1.spept’ by either double-clicking the file, or using ‘File🡺Open’ from the Snoopy menu. Once opened, the model can be viewed by scrolling around the main window, or by using ‘View🡺Zoom’ to change the Zoom level.

Depending on the version of Snoopy you are using, you may see a warning that you are opening a model built in an older version of Snoopy. If this happens, just click ‘Ok’ to acknowledge and open the model.


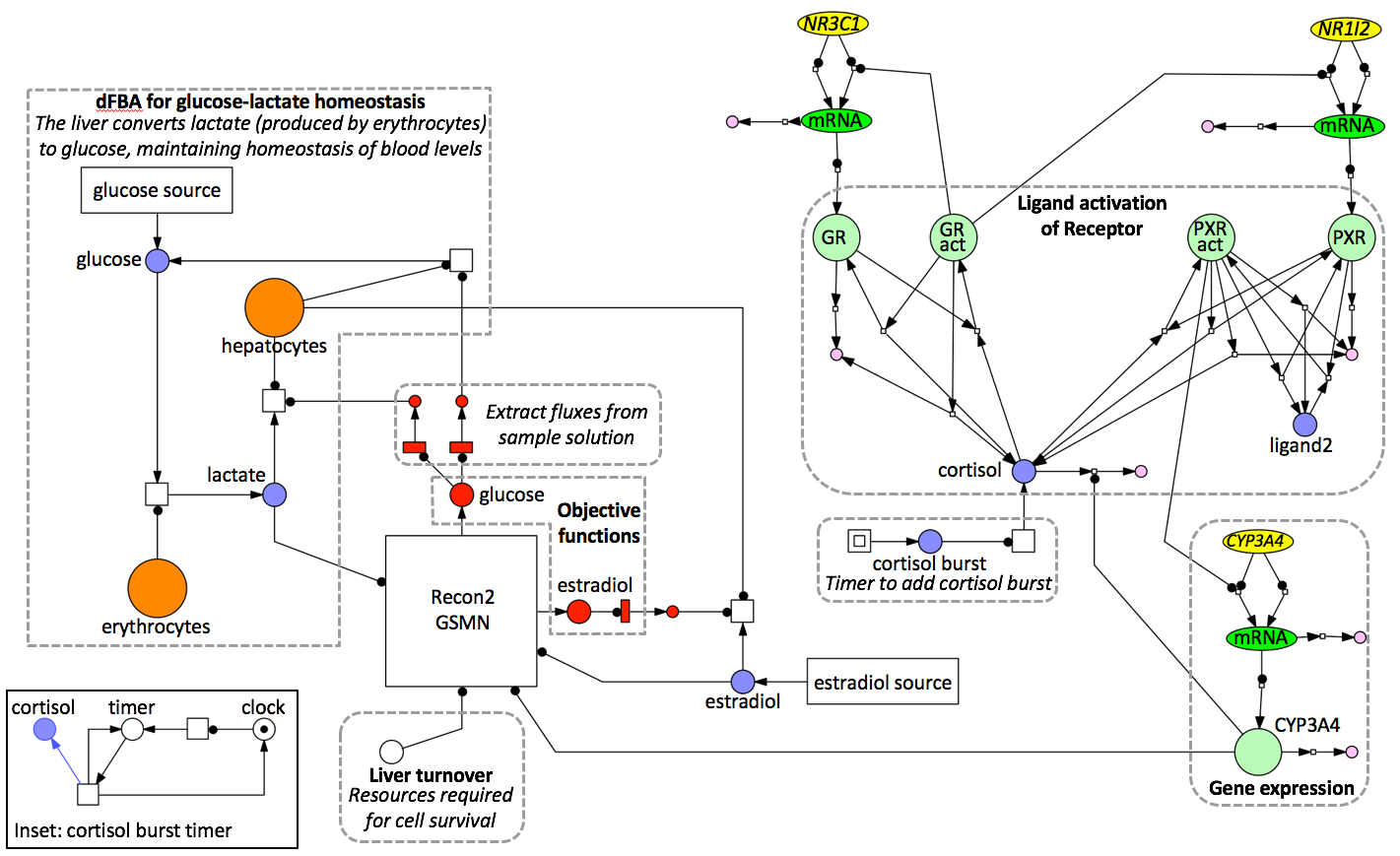


Figure 21: Snoopy model file with additional annotation

In figure 21, the color and symbol size has been manually adjusted to match SBGN molecule types and transition types specific to QSSPN. This is not necessary for simulation, but does aid interpretation of the network topology. Petri Net places (circles and ovals) represent molecular species, and are connected through transitions (rectangles) that represent reactions: the place before a transition is referred to as the pre-place and inputs into the reaction, while post-places occur after the transition and receive outputs from the transition. Places and transitions may be linked through directed arcs, read arcs or inhibitory arcs, which have distinct functions. Directed arcs transfer tokens from the pre-place to the post-place. In comparison, read and inhibitory arcs do not lose tokens from the pre-place to the post-place. Rather, they act as activators or inhibitors, with tokens in the pre-place required/absent for a reaction to occur (respectively), but are not consumed by the reaction.

The model is constructed of several functional units, indicated by the grey-hatched boxes.

*Gene expression* is represented as both transcription and translation reactions, with associated degradation of the mRNA and protein products. Read arcs (line with filled circle) are used as both transcription and translation do not consume their substrates (i.e. gene and mRNA).

*Ligand activation of receptors* represents the interaction of cortisol with the glucocorticoid receptor (GR) and pregnane X-receptor (PXR). Here, directed arcs (line with arrow head) are used to represent movement of species during reactions. In addition, the presence of a second ligand for PXR (ligand2) is simulated, allowing the examination of drug-drug interactions.

*A cortisol infusion* can be simulated through a timer; for clarity this timer is hidden within a coarse transition (double squared box), with the timer shown as an inset to the main diagram.

*Liver turnover* is used to set a constraint on the biomass reaction, representing the minimum level of resources (e.g. amino acids, ATP, nucleotides etc.) required for the hepatocyte to be able to replenish itself.

*Objective functions* set the reactions or metabolites that will be maximized during FBA. In this case, the external metabolites for glucose and estradiol are used. From each FBA solution we extract fluxes that can be used to monitor the behaviour of the system.

*A dFBA* for glucose-lactate homeostasis is included. This adds an additional constraint whereby the GEM must maintain glucose and lactate levels in the blood at 4mM and 1.5mM, respectively.

*Recon2* *GSMN* is a transition that represents the QSSF part of the simulation

### Markup required within the Snoopy file of a QSSPN simulation

All items within a Snoopy representation of a Petri net (i.e. places, transitions and arcs) can have comments attached to them. Double-clicking on the item will open the ‘Edit Properties’ box so that these comments can be accessed. The comments are read by *qsspn* and used to set the parameters for the simulation. The roles of these comments will be described in the following section. Furthermore, to couple the dynamic transition part (Snoopy file) with the quasi steady-state flux part (GSMN) of a QSSPN simulation, a number of special transitions are used within the Snoopy file. These allow *qsspn* to read the interconnections between the two parts and perform the dynamic simulation, and special comments are required for these.

*NOTE:* values in the control file will overwrite any values entered into the comments section of places or transitions within the Snoopy file. For best practice, we recommend that simple (i.e. mass action kinetics) rates be placed within the Snoopy file, while complex (e.g. Michaelis-Menten kinetics) rates be placed within the control file. All initial values that are not default (i.e. 1 or 0) are best placed within the control file.

*NOTE:* you can also use the comments section to enter text comments. If the *qsspn* parser does not recognize a specific command, it will treat it as a text comment and ignore it. Text comments can be useful to add supporting evidence for reactions, such as PubMed IDs for relevant papers, or details on a species, such as Entrez Gene IDs.


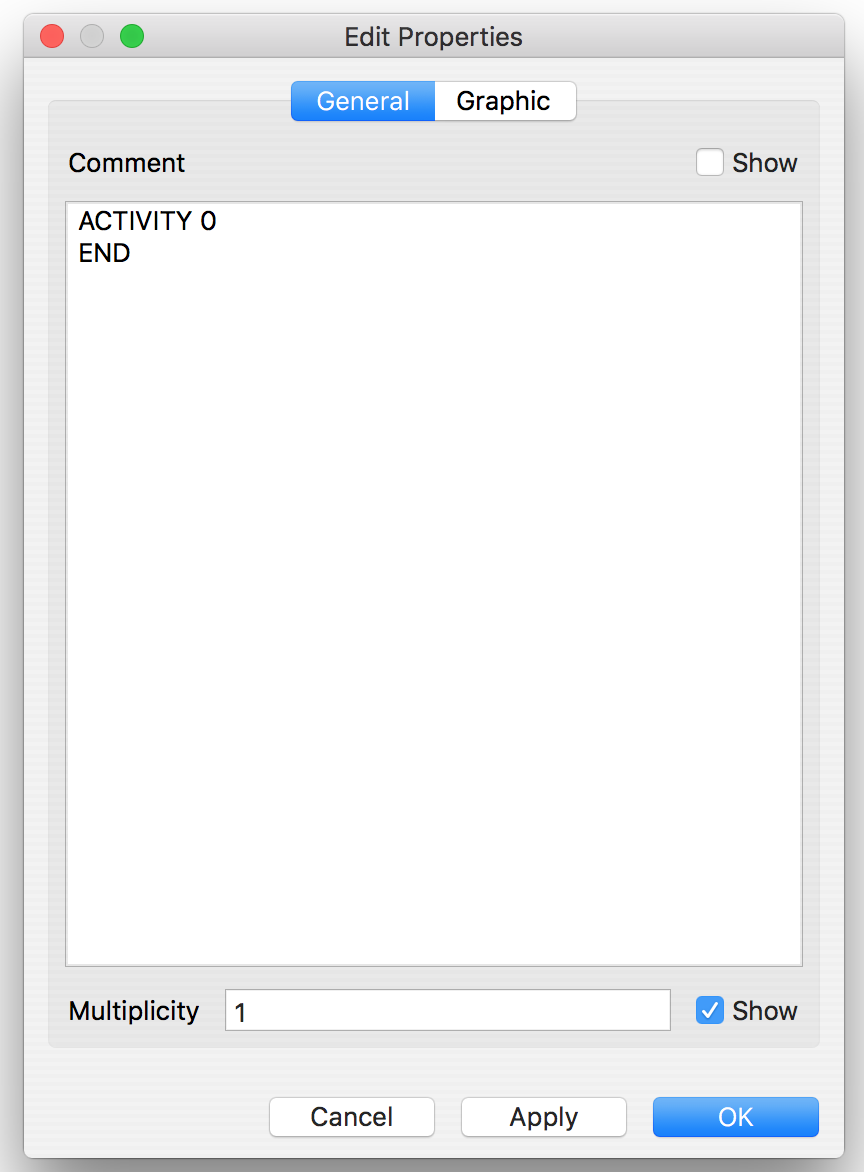


#### Comments within arcs: Setting transition type

Open the ‘Edit Properties’ box for arc between the place ‘lactate’ and the transition ‘F3’. Inside the comments section you will see one entry (Figure 22). The comment section of an edge specifies the activity of the pre-place, in this case Lactate. The ‘ACTIVITY 0’ followed by ‘END’ denotes mass action activity i.e. the marking of the pre-place is used. If in doubt, it is better to enter this comment for any arc connecting a pre-place with a transition in a Petri net. The only exceptions to this are the arcs connecting the objective function to any fluxes that you wish to extract from the FBA example solution – these should be left empty.

Figure 22: Arc comments

####
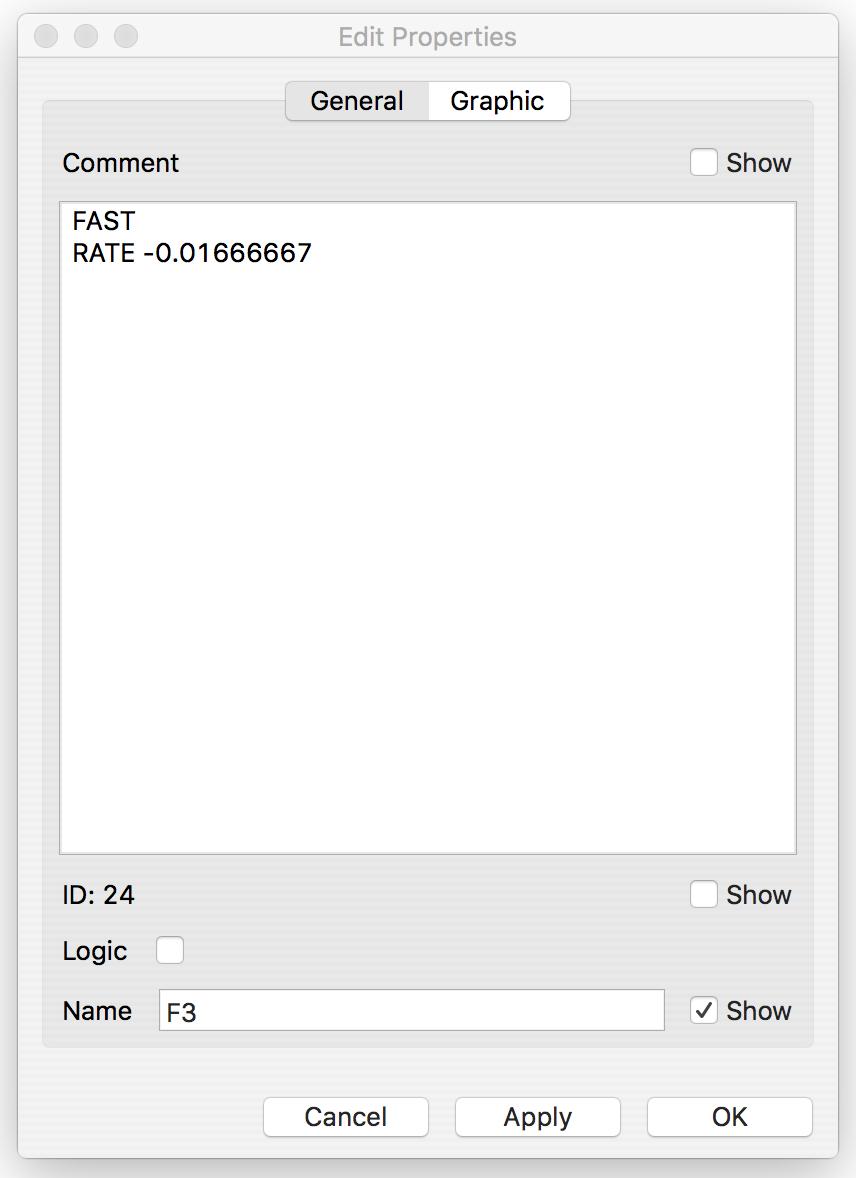
Comments within a transition: Setting rates

Open the ‘Edit Properties’ box for transition ‘F3’. Inside the comments section you will see two entries (Figure 23). The tag ‘FAST’ is used to specify that this is continuous transition. The tag ‘RATE’ is subsequently used to set the flux rate of the transition, in this case to -0.01666667 1/min. If no ‘RATE’ tag is used, then a rate of 1 is set by default. Alternatively, a rate can be taken from the control file.

Figure 23: Setting the flux

value for a transition

####
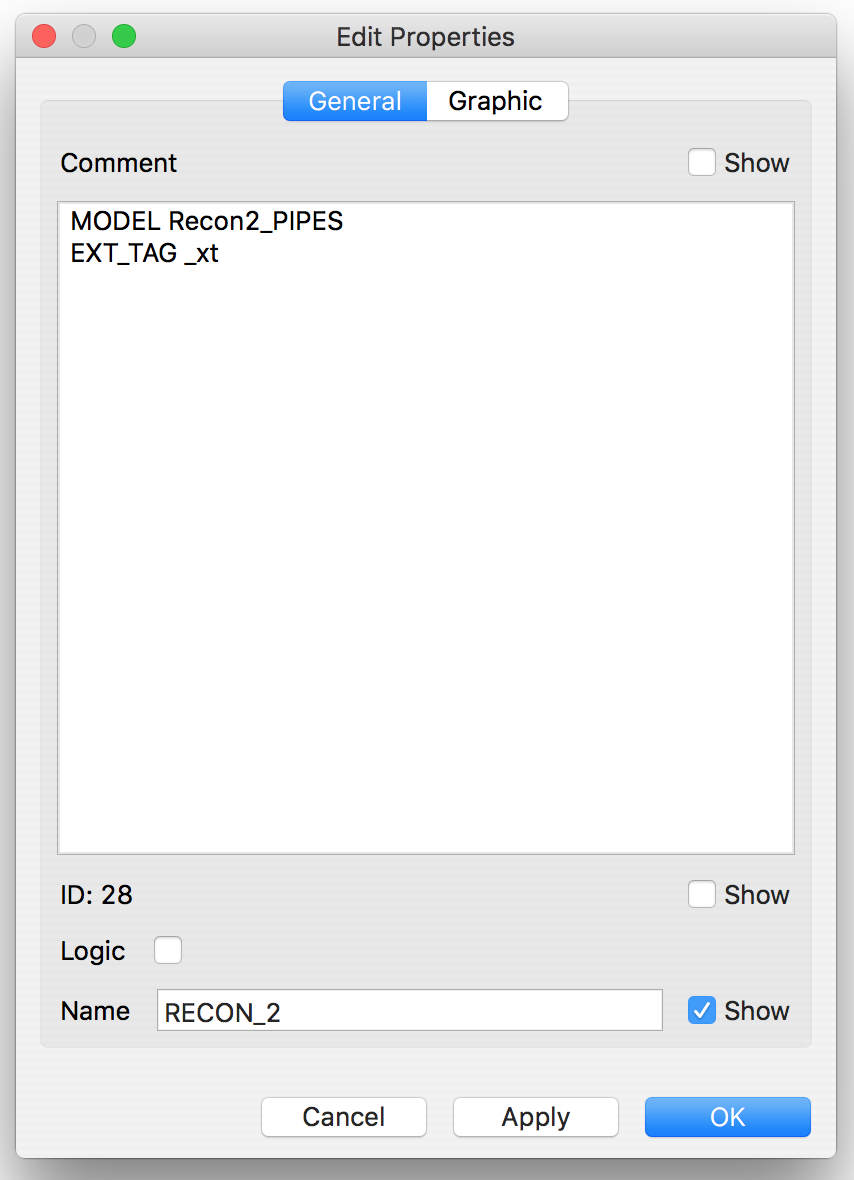
Comments within a transition: Defining the GSMN

Within Snoopy, the Recon2 GSMN is represented as a transition named ‘Recon_2’. Within the comments section of this transition, two command lines are added: first ‘MODEL Recon2_PIPES’ set the transition type as a link to a model; second, ‘EXT_TAG _xt’ specifies the external tag used within the GSMN (Figure 24).

Figure 24: Comments section

for the Recon2 transition

#### Comments within places: Setting activity for mapped reactions


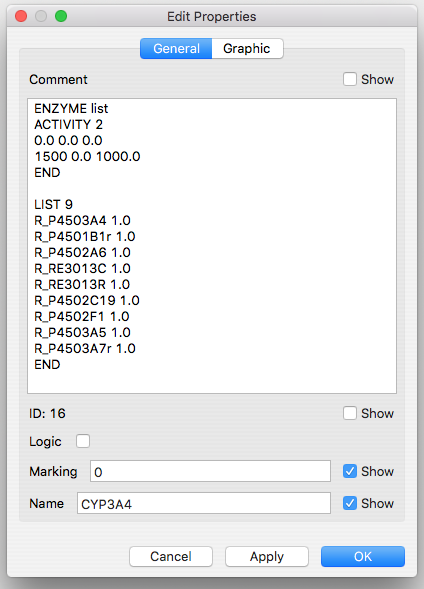
Connection between the continuous PN representing the kinetic model of cortisol signaling and the GSMN occurs through the QSSPN constraint place representing the CYP3A4 protein, as denoted by the read arc between the CYP3A4 protein place and the Recon2 transition. To provide a conversion between the expression level of CYP3A4 protein in the continuous Petri net and bounds for all reactions catalysed by CYP3A4 within Recon2 a lookup table is added to the comment section of the CYP3A4 protein place (Figure 25). As shown in the figure, this lookup table is composed of two parts: a list of all reactions that the protein catalyses within Recon2, and a set of activity values. Where only a single reaction is mapped to a protein, then this can be entered directly next to ‘ENZYME’, but where multiple reactions are effected a list is used (as shown here). The number next to each reaction (e.g. 1.0) sets the relative impact of the activity list on each reaction, allowing differential control of reactions by a protein.

Figure 25: Mapping protein

levels to reaction activity

The activity list sets the bounds for these reactions at any given token status for the protein place. In this case, the flux bounds of all nine reactions are closed (0, 0) for CYP3A4 concentrations smaller than 1500 nM and fully open (0, 1000) at higher concentration. This threshold is sufficient parameterisation for the conclusions presented in this use case, but we note that lookup tables with more levels could be constructed to interpolate rate equation of CYP3A4 enzyme and quantitative parameters.

To link token status within a Petri net representation of a gene or signaling regulatory network with reaction bounds within a GSMN, comments are added to the QSSPN constraint place (e.g. place representing a metabolic enzyme). The comments specify, (i) which reactions are linked to the constraint place, and (ii) the activity of these reactions for any given token status of the constraint place.

#### Comments within places: Defining the objective function and extracting fluxes of interest

To demonstrate the use of comments within places, we have used MUFINS to integrate a simple PBPK constraint with a metabolic reconstruction. This produces a dynamic FBA model that quantitatively constrains the Recon2 GSMN through an ODE model of lactate and glucose concentration in blood. A major physiological function of the liver is to achieve homeostasis for a range of chemicals within the body, including glucose and lactate. Erythrocytes (red blood cells; RBCs) produce lactate during normal cellular respiration, which is then excreted into the blood. As elevated lactate levels may be toxic, lactate is taken up by the liver and converted to glucose. Secretion of glucose by the liver into the blood is important to maintain blood glucose concentrations for use throughout the body.

The graphical, Petri net representation of the model is shown in Figure 21: glucose and lactate in the blood were modelled as Petri net places, with continuous marking representing their concentration in mM. Lactate is formed from glucose in the blood: to scale lactate production to a physiological level, an additional Petri net place was connected to this transition via a read arc, representing the total number of RBCs in the blood (Figure 21). The produced lactate concentration in the blood was modelled as QSSPN constraint place, representing the availability of lactate for the lactate exchange reaction in the Recon2 GSMN. As shown in the right panel of figure 26, if lactate concentration is below 0.001 mM, the exchange reaction is constrained to (0, 1000), preventing uptake of lactate. Otherwise, the lactate exchange reaction flux is set to (-0.0565, 1000) representing a saturating lactate uptake rate of 0.0565 mmol/gDW/h, consistent with experimental observations.

The metabolic utilization of lactate to form glucose in the liver has been represented by setting the FBA objective function as maximization of external glucose. This objective function has been represented by the QSSPN objective place glucose (Figure 21). Commands placed within the comments section of this place specify the metabolite to optimize (external glucose), plus an activity table to prevent the use of this objective if it is infeasible (Figure 26, middle panel).

Finally, flux values are extracted from the FBA solution using QSSPN flux transitions (figure 26, right panel). These are connected to the objective function via a read arc, and access values of the FBA solution for the specified flux fluxes. A directed arc transfers this flux value to a place, where it can be read (Figure 21). The token values for these flux places are then used as inputs for rate equations within transitions representing glucose production and lactate consumption by the hepatocyte. To scale the simulated flux values to a physiological level, these transitions are modified by a Petri net place representing the total number hepatocytes, connected via a read arc (Figure 21).

To represent the net contribution of the rest of the body to glucose blood levels, a glucose source transition is added. This transition adds glucose to the blood at the rate required to achieve the known steady-state glucose blood concentration (-2.76 mmol/L/min).


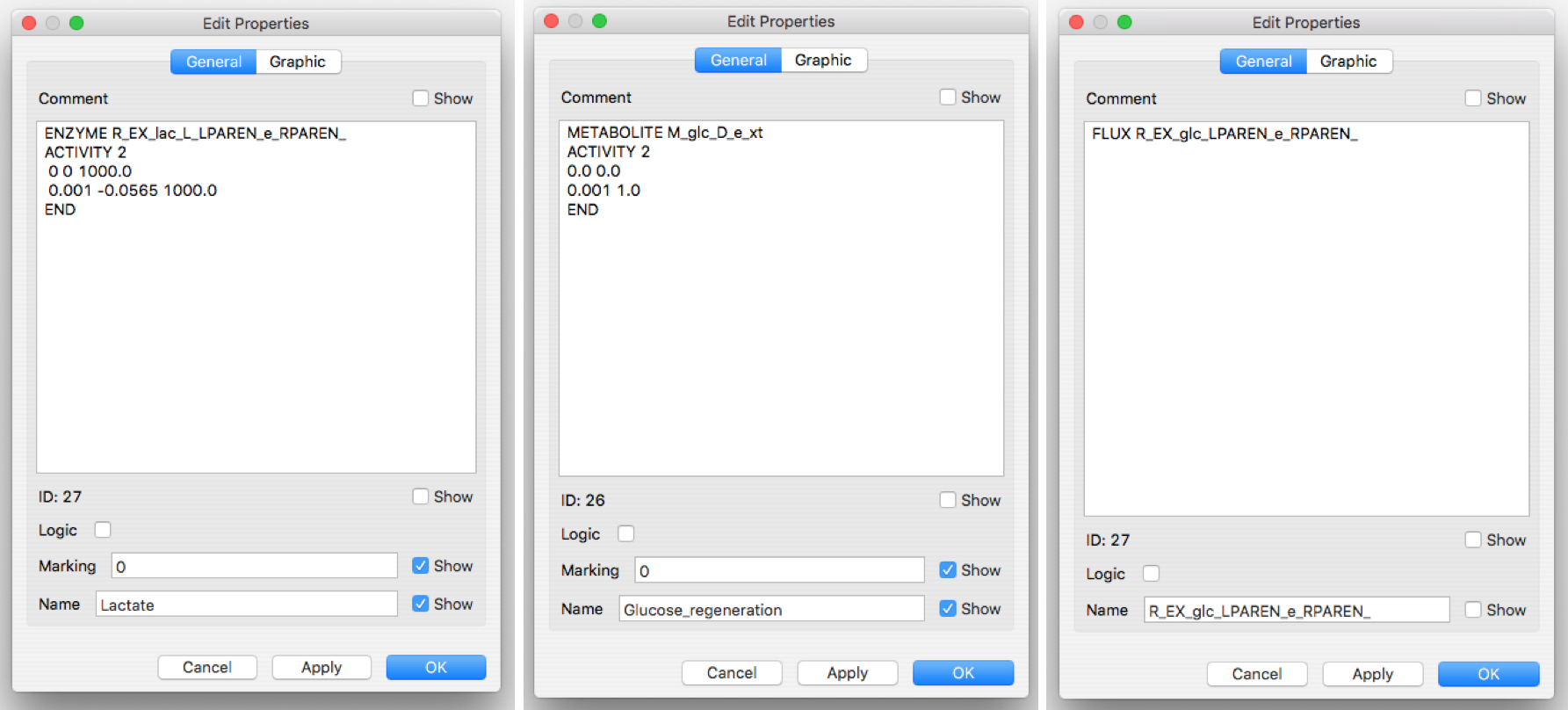


Figure 26: Generation of physiological compartment dynamics for QSSPN simulation

Properties are shown for: QSSPN constraint place for lactate consumption (right), QSSPN objective place to set the objective function for FBA as maximization of production of external glucose (middle), and QSSPN flux transition to extract flux value for glucose exchange from the FBA solution in the pre-place.

### Introduction of a perturbation using Snoopy

A key part of dynamic simulations is the requirement to cause perturbations to the system and then observe the results. We have designed Petri net representations of a simple timer, allowing such perturbations to occur at a specified time, or at a specified frequency. In this exercise, we use a time to start an infusion of cortisol after 500 minutes of simulation. We also use this example to show how coarse transitions can be used to encapsulate technical network into coarse places or transitions, clarifying the biological network diagrams.

To view the sub-network, first ensure the Hierarchy window is visible (Figure 27). If this is not visible use ‘View🡺Toggle Hierarchy browser’ to make it appear. Double clicking on the item ‘cortisol_burst’ will open a new window containing the sub-network (Figure 27)


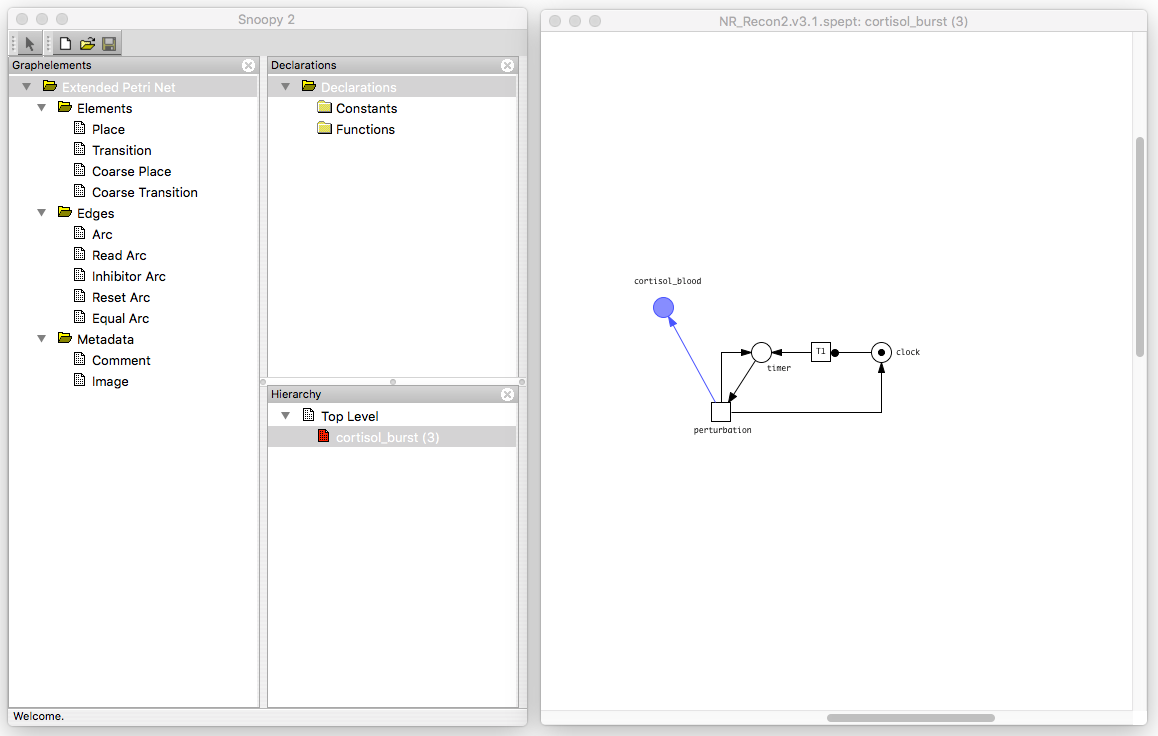
Figure 27: The sub-network ‘cortisol_burst’

The perturbation timer consists of several components.

Place ‘clock’: This place contains a single token and acts as a pre-place for the transition F1

Transition ‘T1’: This transition connects the pre-place ‘clock’ with the post-place ‘timer’. It has a RATE of 1, and is connected to the pre-place by a read edge. This means that every 1 unit of simulation time, a token is added to ‘timer’, but without removing a token from ‘clock’

Place ‘timer’: This place collects tokens from ‘clock’ acting to keep time in the simulation

Transition ‘perturbation’: This transition accepts tokens from the pre-place ‘timer’ and adds them to the post-place ‘cortisol_blood’, which connects to the main network. This place uses a RESET command in the comments section, meaning that the value of ‘cortisol_blood’ is set directly by the number of tokens in the pre-place, rather than receiving them at a specific rate. In this case, the following lines appear in the control file, setting the number of tokens to be transferred to 50

RESET_FUNCTION perturbation

50

END

Arc connecting ‘timer’ to ‘perturbation’: This arc has a look up table that means it is only active when the pre-place reaches 500 tokens (Figure 28). This means that the perturbation will begin after 500 units or simulation time.

*Note:* An alternate method is to set the multiplicity to ‘500’. This would change the perturbation from an infusion (i.e. always on after 500 time units) to a burst (i.e. timer resets after delivering a burst at 500 time units, and begins to accumulate again

Arc connecting ‘perturbation’ to ‘timer’: this arc uses the comment STOCHIOMETRY to set how many of the tokens proceed along this arc. In this case, the value is set to 0, meaning no tokens move along this arc. The reason for including this arc is that when the previous arc is set to produce a burst (i.e. using the multiplicity value) then this is necessary to reset the token status of the ‘timer’ place to zero and reset the timer.

### Running a QSSPN simulation

As with all functions in MUFINS, QSSPN simulations can be undertaken either through JyMet or the command line. For the exercises described under constraint-based modelling, the simulation times are fast and JyMet is the obvious interface to use. However, QSSPN simulation can be longer, depending on the complexity of the model, and hence executing both through JyMet and the command line (especially via a remote server) is more common. Therefore, both ways to exectute a QSSPN simulation will be described below.

#### Running a QSSPN simulation through JyMet:

The implementation of a JyMet interface for QSSPN simulations is major new feature of MUFINS. Upon starting JyMet from, three files must be imported. The first is the GSMN model, designated as recon2_xt.PIPES.CORE.v1.sfba, and can be opened via (File🡺Open Model). Successful import will produce the screen shown in figure 28.


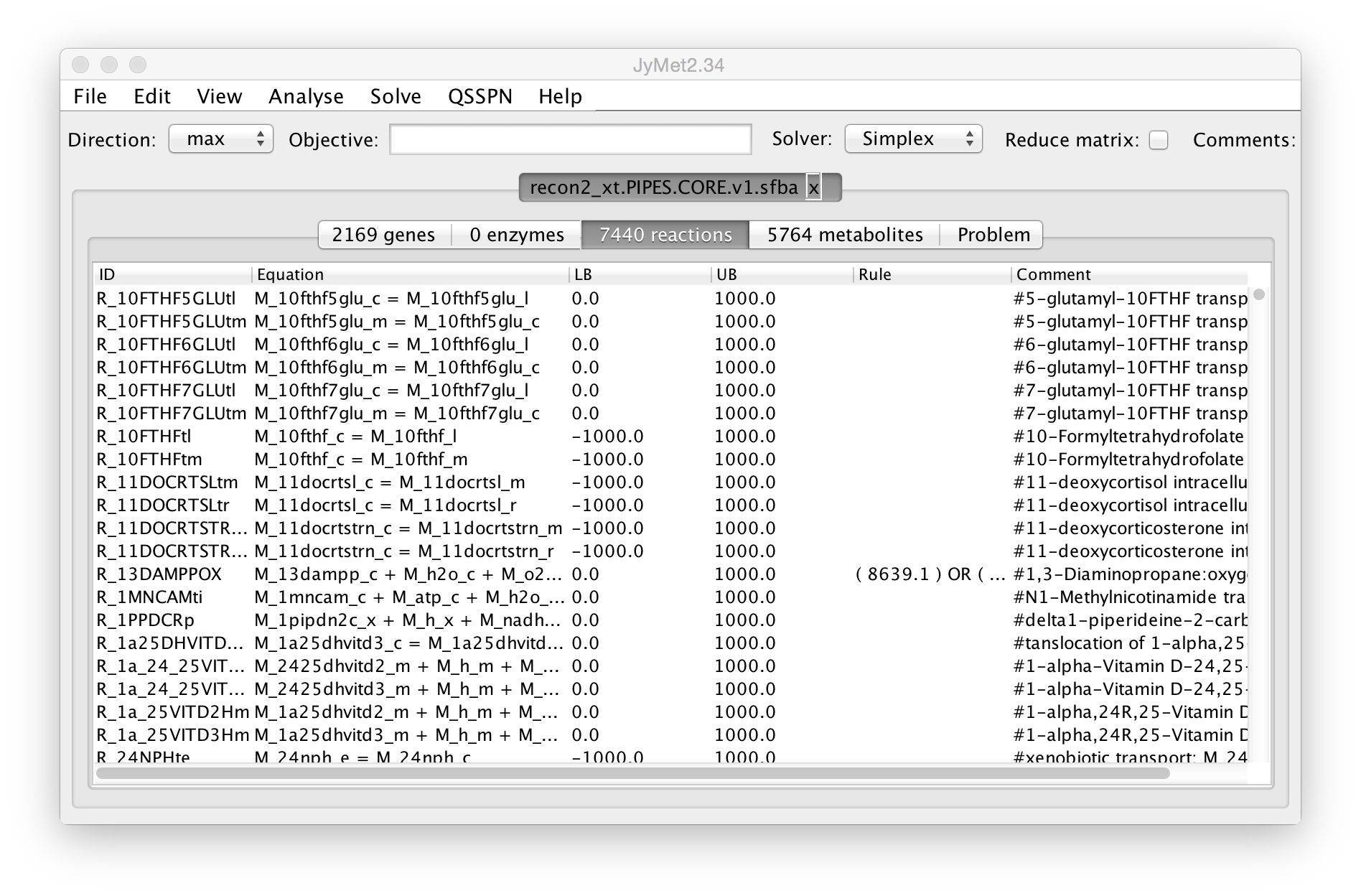
Figure 28: Import of recon2_xt.PIPES.CORE.v1.sfba in JyMet


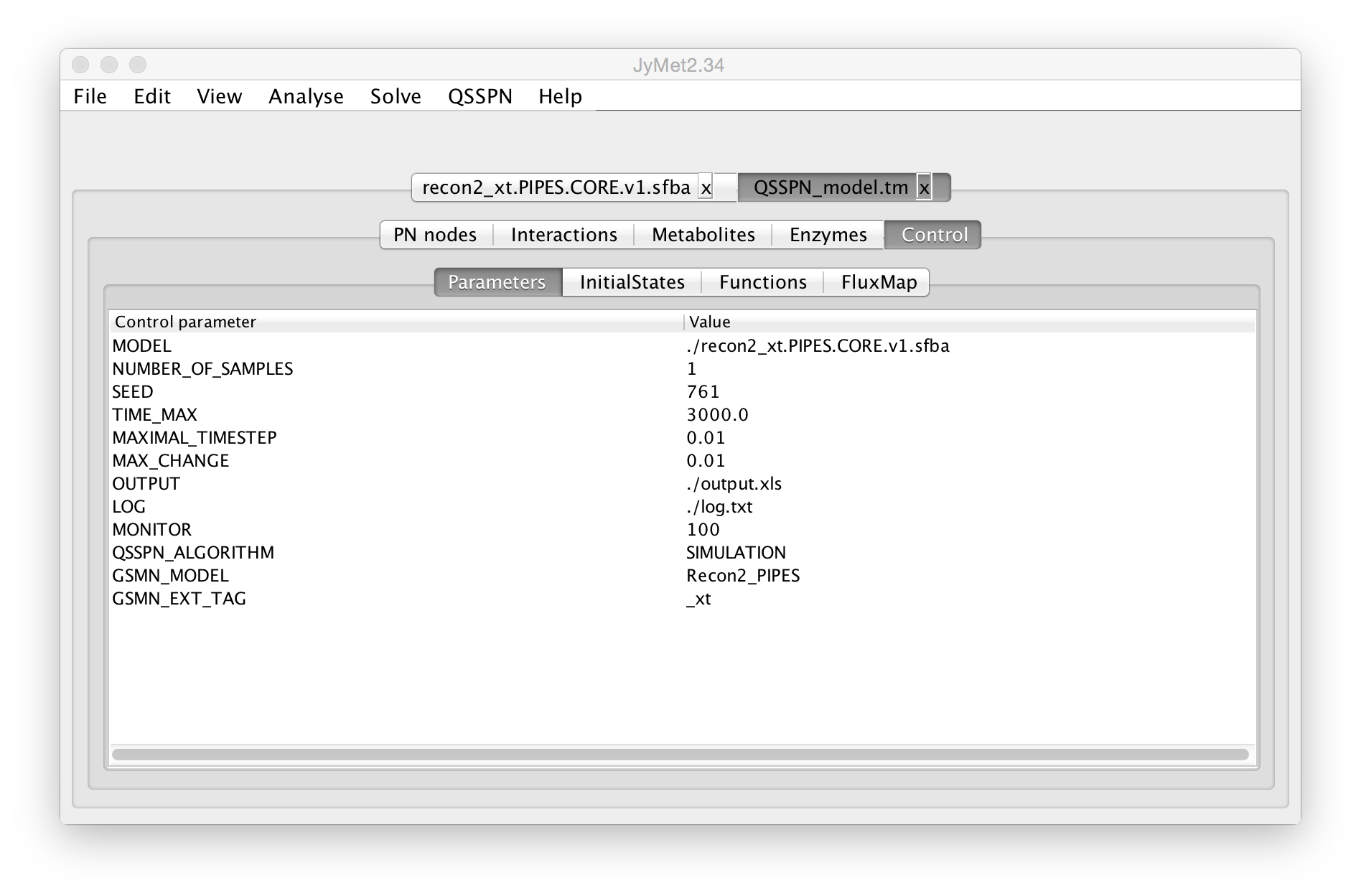
Second, the Snoopy file must be imported (QSSPN🡺Import SPEPT). During this process, the program prompts for to ‘Set default maximal number of tokens’. As commented above, this is best left at 2 for qualitative simulations and 1e9 for quantitative simulations (a value unlikely to be exceeded by any species within the network). Once this import has completed you will see the screen shown in Figure 29. The tabs ‘PN nodes’, ‘Interactions’, provide interface to Petri Net places and Transitions. The ‘Metabolites’ and ‘Enzymes’ tabs contain lookup tables linking Petri Net model to GSMN. All parameters are defined in SurreyFBA**_Docs_qsspn.pdf.

Figure 29: Import of NR_Recon2.v3.1.spept Snoopy file into JyMet


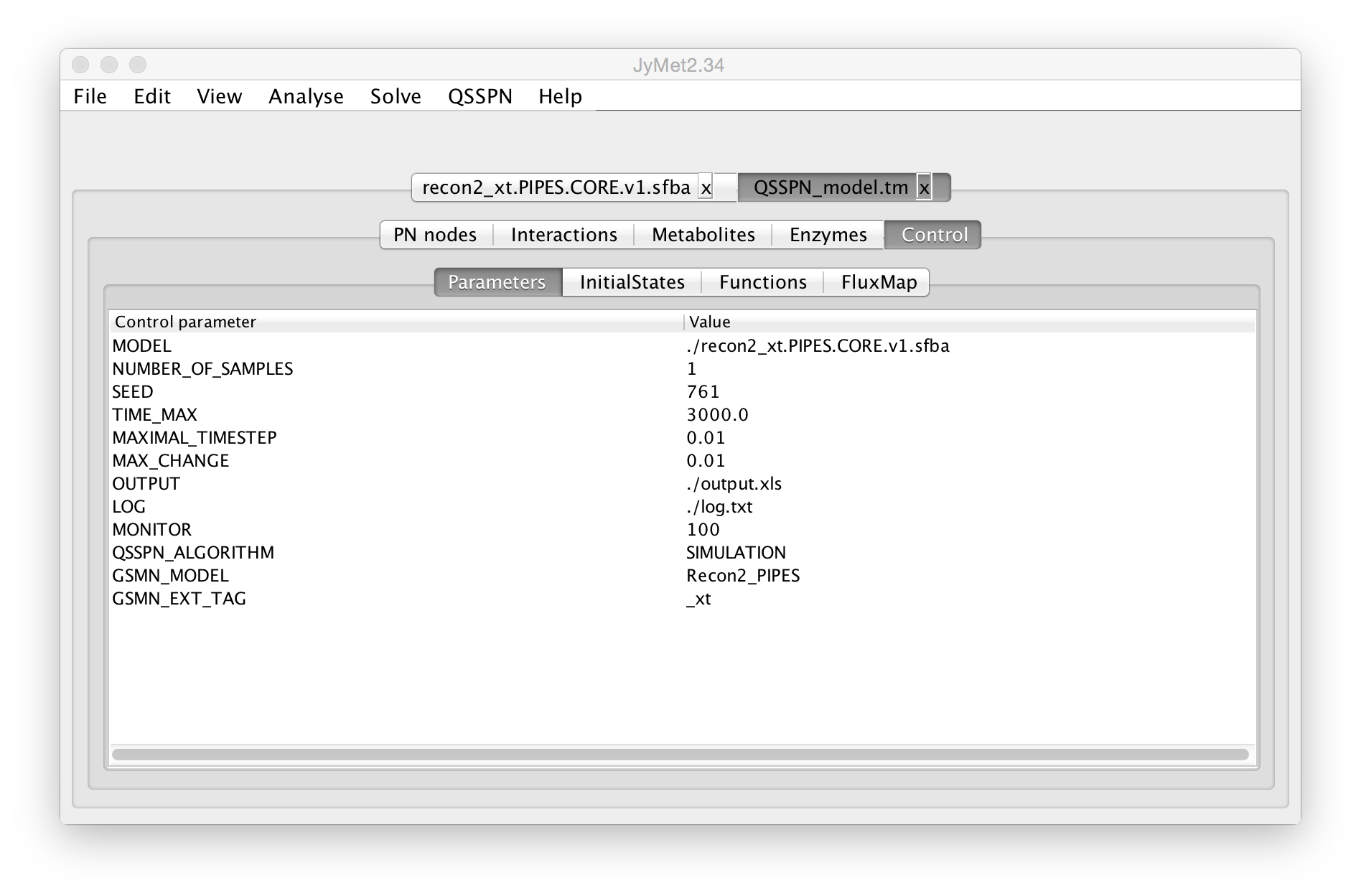
Finally, the control file is loaded (QSSPN🡺Load Control). Figure 30 shows the control file simulation parameters. The ‘InitialStates’ tab sets initial marking of places (i.e. molecular species), while the ‘Functions’ tab allows editing of algebraic formulae for transition propensity functions. The ‘Flux Map’ tab describes reactions where flux values are extracted from the FBA solution.

Figure 30: Import of NR_Recon2.v3.1.ctrl into JyMet


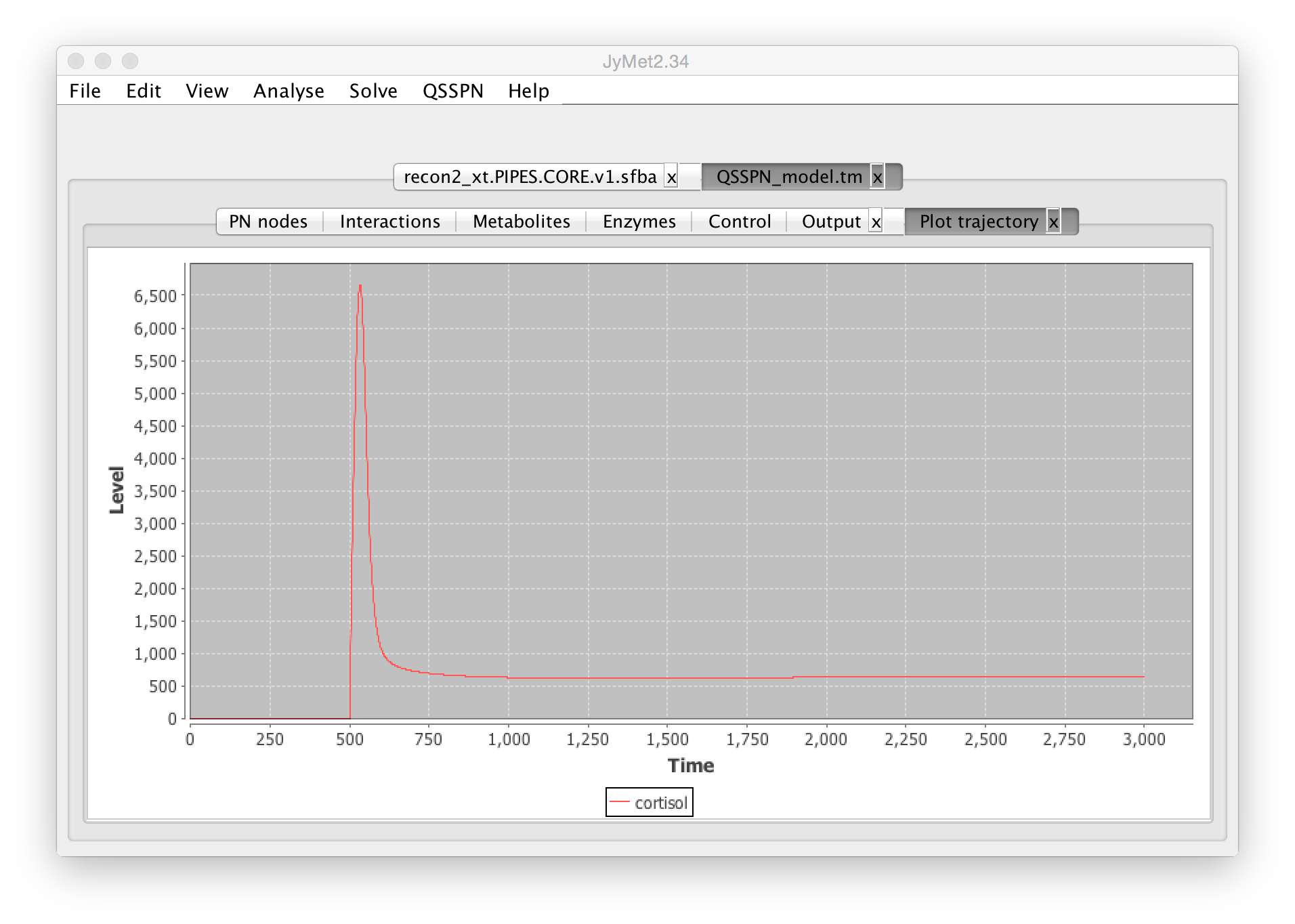
Once all the necessary files have been imported, simulation can be undertaken (QSSPN🡺Run). Once the simulation has finished, the ‘Output’ tab will appear, containing data from all Petri net places specified in the control file. These can be visualized by selected them and then using (QSSPN🡺Plot Trajectory). An example trajectory plot is shown in figure 31.

Figure 31: Example output trajectory for cortisol

A results file is automatically written in the simulation folder, with the name specified under ‘OUTPUT’ in the control file. This can be opened in spreadsheet programs such as Excel for further examination.

#### Running a QSSPN simulation through the Command line:

QSSPN simulations can be run in the command line under all major OS. Instructions for running QSSPN in Mac, Windows and Linux are given below.

Under a Mac OS, start terminal, and navigate to the folder containing the simulation files and enter the following commands

./bin/spept2qsspn name.spept x > name.qsspn

this converts the named *.spept or *.xpn file to a qsspn input format. x specifies the largest possible number used in the simulation. For qualitative simulation, this can usually be set as 2, but for quantitative simulation we recommend 1e9 (a value unlikely to be exceeded by any species within the network).

./bin/qsspn name.qsspn name.ctrl.txt

this executes the simulation. After a brief pause, simulation progress will be seen in the terminal window, with each number representing the simulation time for each MONITOR step.

*Note:* In the example folder for this software distribution the script RUN_Mac will automatically execute the above two commands, while CLEAN_Mac will delete the output files

For Windows OS, the above commands can be used in the Windows Command Line, using Windows syntax. A python interpreter is required to run spept2qsspn. In general, we recommend running simulations using JyMet under Windows OS

For Linux OS, the user should execute two command lines using either binaries available in /bin or go to MUFINS**_Source/QSSPN/ and run compile.sh, install.sh to create new binary in MUFINS**_Source/QSSPN/bin. Both qsspn and spept2qsspn do not require any libraries or environmental variables and can be moved to any convenient location on the system, such as /usr/local/bin.

## QSSPN Semantics Guide

This section provides a detailed guide to the semantics used in creating a QSSPN simulation. It is not designed to be used on its own, but acts as a reference beyond the details provided in the Exercise.

### QSSPN file format

The QSSPN file specifies the Dynamic Transition part of the model as well as constraint and objective places connecting the Dynamic Transitions to Quasi Steady State Fluxes.

The list of Petri net places is defined in the following format:

SUBSTANCES

Name init max type

…

END

Each line between SUBSTANCES and END specifies one Petri net place. The fields have the following meaning:

Name – node name.

init – initial number of tokens associated with the node.

max – the maximal number of tokens.

type – place type, 1 – common place, 2 – objective place, 3 – constraint place.

Subsequently, the text between tags REACTIONS and END specifies Petri net transitions. Each transition is specified by the text between INTERACTION and END tags:

INTERACTION

SUBSTRATE Name rate type delay

ACTIVITY n

t_1_ a_1_

…

t_n_ a_n_

END

END

PRODUCT Name

CONSUMED Name

END

Each SUBSTRATE and PRODUCT tag specifies a Petri net pre-place or post-place, respectively. The CONSUMED tag specifies which of the pre-places change state upon firing of the transition. When transition fires it removes tokens from pre-places specified as CONSUMED and adds tokens to post-places. Thus, activators and inhibitors feature as transition pre-places, but are not specified as CONSUMED. The ACTIVITY section is associated with a particular pre-place and provides a lookup table for the contribution of the pre-place to the transition propensity function (defined later).

The ACTIVITY table with 0 rows:

ACTIVITY 0

END

indicates that a particular pre-place has mass-action activity function in the transition. This is implemented to support integration of quantitative models.

The variables have the following meaning:

Name – the name of Petri net place.

rate – transition rate.

type – transition type, SLOW (stochastic), INSTANT (immediate), FAST (continuous), FLUX (flux, new feature), RESET (reset, new feature).

delay – Applies to SLOW (stochastic) transitions only. If delay is greater than 0 transition is treated as delayed with delay time set by this parameter.

t, a – Threshold and propensity function contribution. If the state of the pre-place node is greater or equal to t in particular row of the table and smaller than t in next row than the pre-place contribution to propensity function is set to a.

The text between GSMN and END describes connectivity between Dynamic Transitions (regulation) and Quasi Steady State Fluxes (metabolism). MODEL tag can be used to specify the path to MUFINS reaction table file with the QSSF model. Settings of MODEL tag are overridden by the value of MODEL tag in the control file. The EXT_TAG specifies externality tag in MUFINS file. This is the string at the end of metabolite name, which indicates external (unbalanced) metabolites in FBA model.

The mapping of flux transition to a particular QSSF flux is defined as:

FLUX transition flux

Where, transition is a transition name in the INTERACTION section of the qsspn file, and flux is the reaction name in the MUFINS reaction table. In this case, the transition type must be set as FLUX in the INTERACTION section. The flux transition must have an objective place as the only pre-place and must be connected to only one post-place.

The objective place is specified by the METABOLITE tag:

METABOLITE Name Objective

ACTIVITY k

t_1_ n_1_

…

t_n_ n_k_

END

END

Where, Name is the name of Petri net objective place and Objective is the name of flux or metabolite in the QSSF. The activity table maps real values of linear programming optimization of QSSF objective to integer number of tokens.

The constraint node is specified by ENZYME tag:

ENZYME Name Flux

ACTIVITY k

n_1_ lb_1_ ub_1_

…

n_k_ lb_k_ ub_k_

END

END

Where, Name is the name of constraint node and Flux is the flux in the QSSF that is constrained. The activity table maps an integer number of tokens n into lower (lb) and upper (ub) flux in QSSF.

Alternatively, the activity table can be applied to a list of fluxes rather than a single flux. This enables modelling of enzymes that catalyze multiple reactions. The flux name is replaced by ‘list’. The list of m fluxes is then provided between LIST and END tags. Each flux name is associated with a weight w, further increasing the flexibility of expressing enzyme-reaction relationships.

ENZYME Name list

ACTIVITY k

n_1_ lb_1_ ub_1_

…

n_k_ lb_k_ ub_k_

END

LIST m

Flux_1_ w_1_

…

Flux_m_ w_m_

END

END

### QSSPN control file format

The HepatocyteQSSPN.ctrl.txt file can be edited with text editor. It specifies parameters of the simulation. The file has the following format:

MODEL path

NUMBER_OF_SAMPLES N

SEED seed

TIME_MAX tmax

MAXIMAL_TIMESTEP timestep

MAX_CHANGE change

OUTPUT out

LOG log

MONITOR m

PROGRESS progress

TARGET name t

INITIAL_STATE

name value

…

END

PROPENSITY_FUNCTION name

formula

END

RESET_FUNCTION name

formula

END

PETRI_NET_MONITORS

name

…

END

SIMULATION

The path specifies the file containing the metabolic network in a MUFINS reaction table format. On some Linux distributions, the full path to the file may be required. The parameter N indicates the number of independent trajectories starting from the same initial conditions. The seed is the seed of random number generator. The tmax is the maximum simulation time. Simulation of a trajectory stops after the maximal time is reached. The maximal timestep parameter of the QSSPN algorithm is set to timestep. It is a maximal change of place marking in an adaptive timestep Euler algorithm used in the fireContinuousTransitions function. The default value of 0.01 indicates 1% change of place marking.

The INITIAL_STATE and END tags enclose initial marking of PN places. The name is the place name and value is its marking at the start of simulation. This feature has been introduced to allow specification of continuous place marking by real numbers rather than integers, as the Snoopy editor interface supports only integer marking.

The major new feature of QSSPN is use of algebraic formulas to define transition propensity functions and reset functions. The PROPENSITY_FUNCTION name, END lines enclose algebraic formula used to calculate a propensity function of transition ‘name’. The formula uses pre-place names as variables. Parameters must be introduced as values. Alternatively, parameters may be introduced as pre-places connected to the transition with read edges. The parameter values may be then specified via the INITIAL_STATE section. The pre-place names can be linked with ‘+’, ‘-’, ‘*’, ‘/’ and ‘^’ (power) operators, and brackets can be used. In the current implementation we do not use functions. The RESET_FUNCTION, END tags enclose formula that use the RESET transition to calculate the value to which marking of post-places will be set.

The state of the system is recorded after each m iterations of the QSSPN algorithm. Output from for each trajectory is written in a tab separated text format to the file specified by out path. The names of Petri net places to be monitored in the trajectory file need to be listed between PETRI_NET_MONITORS and END tags. Each name should be written in a separate line. The log file reports completion of each trajectory and can be used to monitor progress of the simulation. The log file also contains statistics of trajectory sampling. If the PROGRESS tag is specified, the trajectory number and simulation time are printed to standard output after each progress iterations.

The QSSPN trajectory samples are analyzed to calculate the number of trajectories that exhibit a certain behavior. If the behavior of interest can be expressed as a reachability problem (certain place reaching a certain state) the TARGET tag can be used to facilitate calculations. In the log file the QSSPN software reports the number of trajectories where the target node specified reaches the threshold t. The trajectory is stopped after these conditions have been met. If the user wishes to calculate the sample of trajectories that are not stopped before tmax and analyze them later, a dummy target that can never be reached needs to be specified (any Petri net plac with a threshold larger than its maximal number of tokens).

The SIMULATION indicates the function implementing QSSPN algorithm. Currently only this function is available, but in future versions we may implement functions performing more complex simulation protocols (e.g. place inactivation scans).

# Use of MUFINS to analyze ~omics data in the context of genome-scale metabolic networks

## Overview

The integration of ~omics data with genome-scale metabolic networks has two distinct advantages:

(i) the generation of context-specific genome-scale metabolic networks to allow examination of the emergent behavior of these metabolic landscapes during, for example, disease progression (REFS)

(ii) the additional constraints placed that emerge through integration of ~omic datasets and genome-scale metabolic landscapes has been shown to improve the robustness of predictions made from thse ~omic datasets.

MUFINS contains a number of different analysis methodologies to interpret ~omics data in the context of a GSMN, namely: Gene inactivity moderated by metabolism and expression (GIMME), iMAT and Fast iMAT. (REFS)

This section will detail how to undertake these methods in MUFINS. Analysis can be undertaken through JyMet, but due to the time involved in most of these procedures it is more likely that jobs will be run on a remote server. Therefore, command line options are presented.

## Usage of iMAT method and alternatives.

### Synopsis

./sfba -i model_file -X external_tag -j gexp_file -p problem_type -b problem_file [-o array] [-s solver] [-c] [-f output_file]

where,

-i model_file : input model file in MUFINS reaction table format.

-j gexp_file : input gene expression data file

For iMAT and Fast iMAT methods (-p imat, -p fimat), gene expression data should be pre-processed and discretized as 3 levels: highly (1), lowly (-1) and moderately expressed (0). For example:

Gene Tissue1 Tissue2

g1 1 1

g2 1 -1

g3 -1 1

…

Where, the first column consists of gene names, and each of the following columns is associated with a gene expression profile for particular condition. Since this method was initially formulated to generate ‘tissue-specific models’, the columns are labelled as ‘Tissue*’ in the JyMet interface. Columns are tab-delimited, with ‘NA’ denoting an empty value. The expression data can also be enzyme expression level (protein abundances) which is used in the same way as the gene expressions. For the GIMME methods (-p gimme and –p gimmefva), the expressions are pre-processed absolute expression levels (positive numbers).

-p problem_type : analysis method

The following methods are implemented:

imat[,thr] : iMAT method where thr is positive threshold for active flux, used to establish whether reaction is active or not. If not set, the default value is 1.

fimat: Fast iMAT approach (see MUFINS manuscript).

gimme[,thr]: the objective function of GIMME method is to minimize the fluxes of lowly expressed reactions weighted by the deviations of reaction expression state from predefined threshold thr (default 12) for low expression. The output is similar as FBA.

gimmefva[,thr]: the objective function is the same as gimme, but the output is in FVA format.

-b problem_file : input problem file

Problem file follows the format of FBA described in MUFINS manual (see sfba.pdf). The following parameters are specific to ~omics data analysis:

![expression name] - In ~omics data analysis approaches objective function represents congruency between data and flux distribution, rather than reaction flux. Thus we use the name of ~omics data sample (Tissue) in place as objective function. For gimme and gimmefva, the problem should define reaction bounds which define Required Metabolic Functionalities (RMF) that the cell is assumed to achieve, such as for example growth rate where the biomass reaction should be fixed at specific value.

?[reaction name and gene name] - perform analysis only for specific reactions and genes. If no reaction or gene designated, all reactions and genes will be investigated. Names are single space-delimited.

-o array : expression array

Without the problem file you can directly specify which expression array column in expression table you would like to analyze.

-s solver : solver and algorithms

Both a GNU Linear Programming kit (GLPK) and Gurobi can be used as the solver. For the GLPK solver you can choose simplex, exact or milp[,mip_gap] with mip_gap indicating the tolerance of the mixed integer linear programming (MILP: default mip_gap = 1e-6).

To use the Gurobi solver, set options as -s grb[,tol[,foc]] where 2 parameters can be set. Parameter tol is used to set Dual&Primal feasibility tolerance for the Gurobi solver, tightening this tolerance can produce smaller constraint violations (default: 1e-6, Min: 1e-9, Max: 1e-2). Parameter foc is used to set MILP solution strategy, 4 integer values can be chosen which are 1: focus on finding feasible solutions quickly; 2: focus on proving optimality; 3: focus on moving objective bound; 0 (default): balancing between finding new feasible solutions and proving that the current solution is optimal. If MILP solver is very slow for a problem then try foc=3. Because Gurobi’s MILP is much efficient than GLPK’s MILP, it is better choose Gurobi for problems which need MILP solver (such as imat and fimat) on big models.

The default solver is GLPK simplex.

### Example

#### Run the following commands:

./sfba -i model.sfba -X _xt -j gexp -p imat -o Tissue1 –s grb -c -f out_imat

./sfba -i model.sfba -X _xt -j gexp -p fimat -o Tissue1 –s grb -c -f out_fimat

./sfba -i model2.sfba -X _xt -j gexp_level -p gimme,12 -b problem_gimme -c -f out_gimme_gni

./sfba -i model2.sfba -X _xt -j gexp_level -p gimmefva,12 -b problem_gimme -c -f out_gimmefva_gni

#### Output for iMAT (imat):

| Flux-activity analysis results for expression profile of Tissue1 | | | | | | |
| --- | --- | --- | --- | --- | --- | --- |
|  |  |  |  |  |  |  |
| Flux-activity state for reactions: | | |  |  |  |  |
| Reac(direc) | Active | Inactive | ACT_LEV | State | Comment |  |
| R1 (+) | 4: OPTIMAL | 2: OPTIMAL | 2 | 1 | Reaction | 1 |
| R1 (-) | 0: UNDEFINED | 5: OPTIMAL | -5 | 1 | Reaction | 1 |
| R2 (+) | 4: OPTIMAL | 2: OPTIMAL | 2 | 1 | Reaction | 2 |
| R3 (+) | 4: OPTIMAL | 1: OPTIMAL | 3 | -1 | Reaction | 3 |
| R4 (+) | 4: OPTIMAL | 4: OPTIMAL | 0 | 1 | Reaction | 4 |
| R5 (+) | 4: OPTIMAL | 4: OPTIMAL | 0 | 1 | Reaction | 5 |
| R5 (-) | 0: UNDEFINED | 5: OPTIMAL | -5 | 1 | Reaction | 5 |
| /// |  |  |  |  |  |  |
| Flux-activity state for genes: | | |  |  |  |  |
| Number of genes: 5 | |  |  |  |  |  |
| Gene | Active | Inactive | ACT_LEV | EXP_LEV | Postregulation | |
| g1 | 4: OPTIMAL | 2: OPTIMAL | 2 | 1 | no |  |
| g2 | 4: OPTIMAL | 2: OPTIMAL | 2 | 1 | no |  |
| g3 | 4: OPTIMAL | 1: OPTIMAL | 3 | -1 | up |  |
| g4 | 4: OPTIMAL | 4: OPTIMAL | 0 | 1 | no |  |
| g5 | 4: OPTIMAL | 4: OPTIMAL | 0 | 1 | no |  |
| ; |  |  |  |  |  |  |

Symbol (+) indicates the forward direction of reaction, that is, from left side to right side of the reaction formula in the model, whereas (-) indicates the reverse direction. Active denotes the maximum similarity when reaction forced to be active. Inactive denotes the maximum similarity when reaction forced to be inactive. ACT_LEV denotes the activity state of reactions, that is, Active - Inactive. If ACT_LEV >0, reaction is predicted to be active; if ACT_LEV <0 reaction is predicted to be inactive; if ACT_LEV =0, reaction is predicted to be undetermined. For reversible reactions, the directionality can be judged by comparing the ACT_LEV of both directions, for example, ACT_LEV of R1 (+) is 2 against -5 of R1 (-), so reaction R1 prefers the forward direction. State column denotes reaction state determined by genes expressions and rules, where 1 means highly activated; -1 means lowly activated; 0 means moderately activated.

Flux-activity state for genes can be used to test if the genes have post-translational regulations. For example, the expression level of g3 is -1 (lowly expressed), while its ACT_LEV value is 3 (active), so the corresponding gene could be post-up-regulated. The state of reactions can also give post-regulation information, for example R3 has STATE of -1 (lowly expressed) and its ACT_LEV is 4, so the reaction must be post-up-regulated through its regulation genes or enzyme.

#### Output for Fast iMAT (fimat):

#666666: OPTIMAL (R1 + R1__r + R2 + -1 R3 + R4 + R5 + R5__r)

Reac(direc) Minimum Maximum Activity State Comment

R1 500000: OPTIMAL 500000: OPTIMAL 1 1 #Reaction 1

R1__r 0: OPTIMAL 0: OPTIMAL -1 1 #R1:Reverse

R2 500000: OPTIMAL 500000: OPTIMAL 1 1 #Reaction 2

R3 999999: OPTIMAL 999999: OPTIMAL 1 -1 #Reaction 3

R4 333333: OPTIMAL 333333: OPTIMAL 1 1 #Reaction 4

R5 333333: OPTIMAL 333333: OPTIMAL 1 1 #Reaction 5

R5__r 0: OPTIMAL 0: OPTIMAL -1 1 #R5:Reverse

;

The first row shows the maximized optimal flux 666666 and its state OPTIMAL on the objective function max(R1 + R1__r + R2 + -1 R3 + R4 + R5 + R5__r). Column State denotes reaction state determined by genes expressions and rules and the coefficients are determined by these states. After fixing the nonzero-state reactions' fluxes, the normal FVA then be applied to each zero-state reaction. Let predicted flux range be [Fmin, Fmax], if Fmin >0 or Fmax<0 then reaction is predicted to be active (1); if Fmin = Fmax = 0 then reaction is predicted to be inactive (-1); otherwise reaction is predicted undetermined (0). By this reaction R1, R2, R3, R4 and R5 are active, and reverse reactions R1__r and R5__r are inactive which is consistent with the results of imat. Reactions with suffix ‘__r’ are split reverse reactions for nonzero state reversible reactions.

#### Output for Gene inactivity moderated by metabolism and expression (gimme):

#75: OPTIMAL (3 R6 + 3 R6__r)

BIOMASS 50 0 #Biomass

GROWTH 50 0 #Growth

NR1 0 0 #Nutrient M1

NR2 100 0 #Nutrient M2

NR3 25 0 #Nutrient M3

NR4 0 0 #Nutrient M4

R1 50 100 #Reaction 1

R2 25 100 #Reaction 2

R2__r 0 100 #R2:Reverse

R3 0 100 #Reaction 3

R3__r 0 100 #R3:Reverse

R4 0 0 #Reaction 4

R5 25 200 #Reaction 5

R5__r 0 200 #R5:Reverse

R6 25 9 #Reaction 6

R6__r 0 9 #R6:Reverse

;

The first row shows the minimized optimal flux 75 and its state OPTIMAL on the objective function minimize (3 R6 + 3 R6__r) under maximal growth (biomass fixed at 50). The value 75 is inconsistency score indicating the degree of disagreement between the gene expression data and the assumed objective function under specified required functionalities. Second column is non-unique flux for each reaction; third column is the reaction state determined by genes expressions and rules using the same mapping rule as iMAT method. Reactions with suffix ‘__r’ are split reverse reactions for all reversible reactions.

#### Output for gimmefva:

#75: OPTIMAL (3 R6 + 3 R6__r)

Reac(direc) Minimum Maximum Activity State Comment

BIOMASS 50: OPTIMAL 50: OPTIMAL 1 0 #Biomass

GROWTH 50: OPTIMAL 50: OPTIMAL 1 0 #Growth

NR1 0: OPTIMAL 25: OPTIMAL 0 0 #Nutrient M1

NR2 100: OPTIMAL 100: OPTIMAL 1 0 #Nutrient M2

NR3 0: OPTIMAL 25: OPTIMAL 0 0 #Nutrient M3

NR4 0: OPTIMAL 25: OPTIMAL 0 0 #Nutrient M4

R1 50: OPTIMAL 50: OPTIMAL 1 100 #Reaction 1

R2 0: OPTIMAL 100: OPTIMAL 0 100 #Reaction 2

R2__r 0: OPTIMAL 100: OPTIMAL 0 100 #R2:Reverse

R3 0: OPTIMAL 100: OPTIMAL 0 100 #Reaction 3

R3__r 0: OPTIMAL 100: OPTIMAL 0 100 #R3:Reverse

R4 0: OPTIMAL 0: OPTIMAL -1 0 #Reaction 4

R5 25: OPTIMAL 100: OPTIMAL 1 200 #Reaction 5

R5__r 0: OPTIMAL 75: OPTIMAL 0 200 #R5:Reverse

R6 25: OPTIMAL 25: OPTIMAL 1 9 #Reaction 6

R6__r 0: OPTIMAL 0: OPTIMAL -1 9 #R6:Reverse

;

The results format is the same as output of fimat and the judgement of reaction activities is also the same as fimat method. The fluxes of gimme output are contained within the flux range of gimmefva output. State column denotes reaction state determined by genes expressions and rules, with larger number implying greater certainty that reaction is present.

### Usage of flux activity analysis by the GIM3E method

With the GIM3E method, we can exploit metabolomics data to further constrain the solution space of a flux variability analysis and thus generate more robust context-specific predictions on reaction activities.

Synopsis

./sfba -i model_file -X external_tag -j msig_file -p problem_type -b problem_file [-o objective,array] [-s solver] [-c] [-f output_file]

Description

-j msig_file : Input metabolic signal data file

This is discretized data indicating whether a metabolite is detected (1) or not (0) for a set of metabolites of interest. Here is an example:

metabolite arr1 arr2

m1 1 1

m2 1 0

m3 0 1

…

where, first column consists of metabolite names. From second column, each column is associated with a metabolic signal profile. Columns are tab-delimited, with ‘NA’ denoting empty value.

-o objective,array : objective reaction and metabolic signal array

Without the problem file you can directly specify the objective reaction (often the biomass reaction) to be maximized and specify which metabolic signal profile in the metabolic signal table you would like to analyze, delimited by a comma.

-b problem_file : input problem file

Problem file follows the format of FBA described in MUFINS manual, excepting follows.

![objective,array] -specify the objective reaction and metabolic array column in metabolic signal table you would like to analyze, delimited by a comma.

?[reaction name] - perform analysis only for appointed reactions. If no reaction designated, all reactions will be investigated. Names are single space-delimited.

Example

Run following commands:

./sfba -i model2.sfba -X _xt -j msig -p gim3e -o BIOMASS,arr1 –s grb -c -f out_gim3e

Output for gim3e:

#50: OPTIMAL (BIOMASS)

BIOMASS 49.5: OPTIMAL 50: OPTIMAL ACTIVE #Biomass

GROWTH 49.5: OPTIMAL 50: OPTIMAL ACTIVE #Growth

NR1 0: OPTIMAL 50: OPTIMAL UND #Nutrient M1

NR2 99: OPTIMAL 100: OPTIMAL ACTIVE #Nutrient M2

NR3 1.01e-006: OPTIMAL 25: OPTIMAL ACTIVE #Nutrient M3

NR4 0: OPTIMAL 25: OPTIMAL UND #Nutrient M4

R1 49.5: OPTIMAL 50: OPTIMAL ACTIVE #Reaction 1

R2 1.01e-006: OPTIMAL 25: OPTIMAL ACTIVE #Reaction 2

R2__r 0: OPTIMAL 0: OPTIMAL INACT #R2:Reverse

R3 0: OPTIMAL 25: OPTIMAL UND #Reaction 3

R3__r 0: OPTIMAL 0: OPTIMAL INACT #R3:Reverse

R4 0: OPTIMAL 50: OPTIMAL UND #Reaction 4

R5 1.01e-006: OPTIMAL 25: OPTIMAL ACTIVE #Reaction 5

R5__r 0: OPTIMAL 0: OPTIMAL INACT #R5:Reverse

R6 24.75: OPTIMAL 50: OPTIMAL ACTIVE #Reaction 6

R6__r 0: OPTIMAL 0: OPTIMAL INACT #R6:Reverse

TS_M1 37.125: OPTIMAL 50: OPTIMAL ACTIVE #Turnover sink: M1

TS_M2 74.25: OPTIMAL 75: OPTIMAL ACTIVE #Turnover sink: M2

TS_M3 1.01e-006: OPTIMAL 25: OPTIMAL ACTIVE #Turnover sink: M3

TS_M4 0: OPTIMAL 25: OPTIMAL UND #Turnover sink: M4

TS_M5 74.25: OPTIMAL 75: OPTIMAL ACTIVE #Turnover sink: M5

TS_M6 37.125: OPTIMAL 50: OPTIMAL ACTIVE #Turnover sink: M6

TS_M7 24.75: OPTIMAL 50: OPTIMAL ACTIVE #Turnover sink: M7

TS_M8 49.5: OPTIMAL 50: OPTIMAL ACTIVE #Turnover sink: M8

;

Compared to the output of FVA:

#50: OPTIMAL (BIOMASS)

BIOMASS 50: OPTIMAL 50: OPTIMAL ACTIVE #Biomass

GROWTH 50: OPTIMAL 50: OPTIMAL ACTIVE #Growth

NR1 0: OPTIMAL 50: OPTIMAL UND #Nutrient M1

NR2 100: OPTIMAL 100: OPTIMAL ACTIVE #Nutrient M2

NR3 0: OPTIMAL 25: OPTIMAL UND #Nutrient M3

NR4 0: OPTIMAL 25: OPTIMAL UND #Nutrient M4

R1 50: OPTIMAL 50: OPTIMAL ACTIVE #Reaction 1

R2 0: OPTIMAL 25: OPTIMAL UND #Reaction 2

R3 0: OPTIMAL 25: OPTIMAL UND #Reaction 3

R4 0: OPTIMAL 50: OPTIMAL UND #Reaction 4

R5 0: OPTIMAL 25: OPTIMAL UND #Reaction 5

R6 25: OPTIMAL 50: OPTIMAL ACTIVE #Reaction 6

;

Explanation:

The first row is maximized objective flux value, here is the optimal biomass growth rate. The output table has the same format as FVA output, where, the first column is reaction IDs where reaction IDs suffixed with ‘__r’ represent reverse reactions split for reversible reactions. Reaction IDs headed with ‘TS_’ represent turnover sink reactions for metabolites which have signals in metabolic signal file. The detected metabolites (having signal of 1) will be set positive flux for their turnover sink reactions. The second and third columns are minimum flux and maximum flux for the reactions. The fourth column shows the reaction activities where ‘ACTIVE’ indicates the reaction was predicted to be active; ‘INACT’ indicates the reaction was predicted to be inactive; and ‘UND’ indicates the reaction was predicted to be undetermined. Comparing to the FVA output, we can see that reactions NR2, NR3, R2 and R5 became active in GIM3E output by using metabolic constraints.

3. Usage for predicting gene-nutrient interactions (GNIs)

Synopsis

./sfba -i model_file -X external_tag -p problem_type -b problem_file [-o biomass] [-s solver] [-c] [-f output_file]

Description

-p problem_type : analysis for certain problem

The analysis includes:

sgni : strong GNI analysis.

wgni[,sam] : weak GNI analysis, optional parameter sam is sample size of media for wgni. The number of sampled growth media for wgni problem which should be proportionate to the media space (
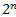
, where n is number of nutrients) is not set, default value is 33000.

-b problem_file : input problem file

Problem file follows the format of FBA described in MUFINS manual, excepting follows.

![nutrient names] - here nutrient names are specified, which defines the space of growth media under which we perform prediction of GNIs. The nutrient names are single space-delimited. The names of nutrients are the same as the name of external metabolites of exchange reactions in model file.

$[nutrient names] - for the rest nutrients excepting defined nutrients of growth medium, here we can designate the nutrients to be set present and rest nutrients will be set absent. If no nutrient designated, all nutrients excepting nutrients of growth medium will be set absent. Names are single space-delimited.

?[gene name] - here you can designate a list of genes for GNI analysis. The gene names are single space-delimited. If no gene designated, all genes will be investigated.

-o biomass: biomass reaction

You have to specify a biomass/growth reaction for GNI analysis.

Example

Run following commands:

./sfba -i model2.sfba -X _xt -p sgni -b problem_gni -o BIOMASS -c -f out_gni_sgni

./sfba -i model2.sfba -X _xt -p wgni -b problem_gni -o BIOMASS -c -f out_gni_wgni

Output for sgni

Gene: g1

KO Reactions: R1

BLP Result: Drop = 50:OPTIMAL Level = 1

Growth Medium:

M1_xt : 1

M2_xt : 1

M3_xt : 0

M4_xt : 0

Nutrients of GNI-SP: M2_xt

Gene: g2

KO Reactions: R6

BLP Result: Drop = 50:OPTIMAL Level = 1

Growth Medium:

M1_xt : 1

M2_xt : 1

M3_xt : 0

M4_xt : 0

Nutrients of GNI-SP: M2_xt

Gene: g3

KO Reactions: R5

BLP Result: Drop = 50:OPTIMAL Level = 1

Growth Medium:

M1_xt : 0

M2_xt : 1

M3_xt : 1

M4_xt : 0

Nutrients of GNI-SP: M2_xt M3_xt

Nutrients of GNI-SN: M1_xt

Gene: g4

KO Reactions: R2 R3

BLP Result: Drop = 50:OPTIMAL Level = 1

Growth Medium:

M1_xt : 0

M2_xt : 1

M3_xt : 1

M4_xt : 0

Nutrients of GNI-SP: M2_xt M3_xt

Nutrients of GNI-SN: M1_xt

Gene: g6

KO Reactions: R4

BLP Result: Drop = 50:OPTIMAL Level = 1

Growth Medium:

M1_xt : 1

M2_xt : 1

M3_xt : 0

M4_xt : 0

Nutrients of GNI-SP: M1_xt M2_xt

Nutrients of GNI-SN: M3_xt M4_xt

;

Explanation:

The predicted results for each gene are delimited by a space line, and only the genes having knockout reactions are listed. Explanation of output format is as follows. Gene: name of gene; KO Reactions: knockout reactions associated with this gene; BLP Result: bi-level LP optimization results, where Drop = growth rate of wild type – growth rate of knockout strains, followed by solution status, Level: the percentage of drop over growth rate of wild type, if Level > 20% then this gene is considered as essential under this growth medium; Growth Medium: consisting of a set of nutrients whose present/absent states are indicated by 1/0; Nutrients of GNI-SP: the predicted nutrients which have strong positive GNIs with this gene; Nutrients of GNI-SP: the predicted nutrients which have strong negative GNIs with this gene. For example gene g4 has spGNIs with M2_xt and M3_xt, and has snGNI with M1_xt.

Output for wgni

The analysis results of weak GNI with sample size of 16

Gene: g1

KO Reactions: R1

Number of Essential medium = 6

Nutrients:

M1_xt GNI-WP: Number = 4 GNI-WN: Number = 2

M2_xt GNI-WP: Number = 6 GNI-WN: Number = 0

M3_xt GNI-WP: Number = 4 GNI-WN: Number = 2

M4_xt GNI-WP: Number = 3 GNI-WN: Number = 3

Gene: g2

KO Reactions: R6

Number of Essential medium = 6

Nutrients:

M1_xt GNI-WP: Number = 4 GNI-WN: Number = 2

M2_xt GNI-WP: Number = 6 GNI-WN: Number = 0

M3_xt GNI-WP: Number = 4 GNI-WN: Number = 2

M4_xt GNI-WP: Number = 3 GNI-WN: Number = 3

Gene: g4

KO Reactions: R2 R3

Number of Essential medium = 2

Nutrients:

M1_xt GNI-WP: Number = 0 GNI-WN: Number = 2

M2_xt GNI-WP: Number = 2 GNI-WN: Number = 0

M3_xt GNI-WP: Number = 2 GNI-WN: Number = 0

M4_xt GNI-WP: Number = 1 GNI-WN: Number = 1

Gene: g3

KO Reactions: R5

Number of Essential medium = 2

Nutrients:

M1_xt GNI-WP: Number = 0 GNI-WN: Number = 2

M2_xt GNI-WP: Number = 2 GNI-WN: Number = 0

M3_xt GNI-WP: Number = 2 GNI-WN: Number = 0

M4_xt GNI-WP: Number = 1 GNI-WN: Number = 1

Gene: g6

KO Reactions: R4

Number of Essential medium = 1

Nutrients:

M1_xt GNI-WP: Number = 1 GNI-WN: Number = 0

M2_xt GNI-WP: Number = 1 GNI-WN: Number = 0

M3_xt GNI-WP: Number = 0 GNI-WN: Number = 1

M4_xt GNI-WP: Number = 0 GNI-WN: Number = 1

;

Explanation:

The predicting results for each gene are separated by a space line, and only the genes having knockout reactions are listed. Explanation of output format is as follows. Gene: name of gene; KO Reactions: knockout reactions associated with this gene; Number of Essential medium: the number of media under which the gene is essential; Nutrients: where a set of nutrients and their predicted results are listed; At each line of a nutrient, GNI-WP: the predicted results of weak positive GNI where the value of Number is the number of presents among all sampled essential growth media. GNI-WN: the predicted results of weak negative GNI. The sum of number of wpGNI and wnGNI is the total number of sampled essential media. In cases where a nutrient is found to be present (or absent) in all sampled media, this may also hint to the existence of strong GNIs. As expected, the predicted wGNI results are consistent with the predicted sGNI results. For example, gene g4 has spGNIs with M2_xt and M3_xt which are shown present in all 2 essential media in the wGNI results. Also g4 has snGNI with M1_xt which is shown absent in all 2 essential media in the wGNI results.

Statistical scripts

To address the statistical significance of wgni results, we offer three scripts for this purpose.

1) Perl script ‘ wgni2table.pl’ - extract wgni results into a table format which can be read for a R script.

Usage:

perl wgni2table.pl wgni_file [ess_gene_file] count_table_file

wgni_file : wgni file from predicted wgni results.

ess_gene_file : a optional file contains a list of essential genes, one gene each line, only these genes will be counted for table, otherwise without this file all genes will be considered.

count_table_file : output file of a count table.

Example:

For above wgni results, run perl command:

perl wgni2table.pl out_gni_wgni table_wgni

get output:

|  | M1_xt.WP | M1_xt.WN | M2_xt.WP | M2_xt.WN | M3_xt.WP | M3_xt.WN | M4_xt.WP | M4_xt.WN |
| --- | --- | --- | --- | --- | --- | --- | --- | --- |
| g1 | 4 | 2 | 6 | 0 | 4 | 2 | 3 | 3 |
| g2 | 4 | 2 | 6 | 0 | 4 | 2 | 3 | 3 |
| g4 | 0 | 2 | 2 | 0 | 2 | 0 | 1 | 1 |
| g3 | 0 | 2 | 2 | 0 | 2 | 0 | 1 | 1 |
| g6 | 1 | 0 | 1 | 0 | 0 | 1 | 0 | 1 |

Note:

Rows correspond with genes and columns correspond with nutrients. The numbers are counts associated with the related genes and nutrients. Column name is formatted as ‘nutrient name.WP(WN)’ where WP(WN) means weak positive (weak negative).

2) R script ‘wgniStat’ - an R function performing statistical computing for input of above table.

Usage:

wgniStat(‘count_table_file’,’stat_table_file’)

count_table_file : count table file from output of wgni2table.pl script.

stat_table_file : output file of the statistical significance for each count in count table.

Example:

For above count table results, run R command:

wgniStat(‘table_wgni’,’stat_wgni’)

get output:

| The p-value for weak GNIs | | |  |  |  |  |  |  |
| --- | --- | --- | --- | --- | --- | --- | --- | --- |
|  |  |  |  |  |  |  |  |  |
|  | M1_xt.WP | M1_xt.WN | M2_xt.WP | M2_xt.WN | M3_xt.WP | M3_xt.WN | M4_xt.WP | M4_xt.WN |
| g1 | 0.1066 | 0.6581 | 0.0000 | 0.9853 | 0.1066 | 0.6581 | 0.3419 | 0.3419 |
| g2 | 0.1066 | 0.6581 | 0.0000 | 0.9853 | 0.1066 | 0.6581 | 0.3419 | 0.3419 |
| g4 | 0.7510 | 0.0000 | 0.0000 | 0.7510 | 0.0000 | 0.7510 | 0.2490 | 0.2490 |
| g3 | 0.7510 | 0.0000 | 0.0000 | 0.7510 | 0.0000 | 0.7510 | 0.2490 | 0.2490 |
| g6 | 0.0000 | 0.5000 | 0.0000 | 0.5000 | 0.5000 | 0.0000 | 0.5000 | 0.0000 |

Explanation:

The number is estimated p-value indicating the statistical significance of weak GNIs, the smaller this p-value the more significant the GNI is, normally it is considered as weak GNI if p-value < 0.05. It should be noted that given a media space the larger sampled essential media space the more statistically reliable the p-value would be, and also the larger the media space the more sampled essential media are needed to satisfy reliable statistics. This may be problematic for genes that are essential for growth in a small fraction of the media space.

3) R script ‘predictMedia’ - an R function used for predicting growth medium composition.

Usage:

predictMedia(‘count_table_file’, ‘media_file’, threshold)

count_table_file : count table file from output of wgni2table.pl script.

media_file : output file contains predicted statistical results of growth medium composition.

threshold : a p-value threshold used for counting the number of weak GNIs for each nutrient, the smaller

this threshold the more conservative the predicted results could be.

Example:

For above count table results, run R command:

predictMedia(‘table_wgni’, ‘media’, 1e-7)

get output:

|  | Presence | Absence | Sum | Pval_Pre | Pval_Abs |
| --- | --- | --- | --- | --- | --- |
| M1_xt | 1 | 2 | 3 | 0.9167 | 0.5 |
| M2_xt | 5 | 0 | 5 | 0.0040 | 1 |
| M3_xt | 2 | 1 | 3 | 0.5 | 0.9167 |
| M4_xt | 0 | 1 | 1 | 1 | 0.5 |

Explanation:

Presence : number of weak positive GNIs for each nutrient; Absence : number of weak negative GNIs for each nutrient; Sum = Presence + Absence; Pval_Pre : p-value indicating the statistical significance of presence; Pval_Abs : p-value indicating the statistical significance of absence. The smaller the p-value the more significant the presence/absence would be. We can set a cut-off of p-value (such as 0.05) used for judging if the nutrient present or absent.

4. Usage for differential producibility analysis (DPA)

Synopsis

./sfba -i model_file -X external_tag -j gexp_file -u dpaplot_file -p problem_type -b problem_file [-c] [-f output_file]

Description

-j gexp_file : Input gene expression data file (only for dpasig)

Gene expression data should be in the form of log2 ratios of treatment and reference sample signals,

no processing of data was conducted; Here is an example:

Gene Array1 Array2

g1 2.5 -1.8

g2 1.3 - 1.5

g3 -1.6 1.6

…

Where, first column consists of gene names. From second column, each column is associated with a microarray name and their gene expression ratios. Columns are tab-delimited, with ‘NA’ denoting empty value. This file is only used for problem dpasig.

-u dpaplot_file : the output file of problem dpaplot (only for dpasig)

This file is only used for problem dpasig. This file provides the mapping from genes to metabolite to that will be used to calculate the metabolite signals.

-p problem_type : analysis for certain problem

The analysis includes:

dpaplot : DPA analysis of producibility plot using GLPK library

dpaplotGrb: DPA analysis of producibility plot using Gurobi library

dpasig : DPA analysis of metabolite signals

-b problem_file : input problem file

Problem file follows the format of FBA described in MUFINS manual, excepting follows.

![array name] - for problem dpaplot, just leave it empty; for problem dpasig, we can designate microarray names of interest, if not set all microarrays in gexp_file will be used. The array names are single space-delimited.

Example

Run following commands:

./sfba -i model2.sfba -X _xt -p dpaplot -c -f out_dpaplot

./sfba -j gexp_ratio -u out_dpaplot -p dpasig -b problem_dpasig -c -f out_dpapsig

Output for dpaplot

| Metabolites to genes: | | | |  |  |
| --- | --- | --- | --- | --- | --- |
|  |  |  |  |  |  |
| M3 | g4 |  |  |  |  |
| M4 | g4 | g6 | g3 | g2 |  |
| M5 | g1 | g3 |  |  |  |
| M6 | g6 | g3 | g2 |  |  |
| M7 | g1 | g4 | g6 | g3 | g2 |
| M8 | g6 | g2 |  |  |  |
| M_BIOMASS | g1 | g2 |  |  |  |
| /// |  |  |  |  |  |
|  |  |  |  |  |  |
| Genes to metabolites: | | | | |  |
|  |  |  |  |  |  |
| Gene: | g1 |  |  |  |  |
| KO Reactions: | R1 |  |  |  |  |
| Metabolites: | M5 | M7 | M_BIOMASS |  |  |
|  |  |  |  |  |  |
| Gene: | g2 |  |  |  |  |
| KO Reactions: | R6 |  |  |  |  |
| Metabolites: | M4 | M6 | M7 | M8 | M_BIOMASS |
|  |  |  |  |  |  |
| Gene: | g3 |  |  |  |  |
| KO Reactions: | R5 |  |  |  |  |
| Metabolites: | M4 | M5 | M6 | M7 |  |
|  |  |  |  |  |  |
| Gene: | g4 |  |  |  |  |
| KO Reactions: | R2 | R3 |  |  |  |
| Metabolites: | M3 | M4 | M7 |  |  |
|  |  |  |  |  |  |
| Gene: | g6 |  |  |  |  |
| KO Reactions: | R4 |  |  |  |  |
| Metabolites: | M4 | M6 | M7 | M8 |  |
| /// |  |  |  |  |  |
|  |  |  |  |  |  |
| Matrix of producibility of wildtype and knockouts: | | | | | |
|  |  |  |  |  |  |
|  | WildType | g1 | g4 | g6 | g3 |
| M1 | 100 | 100 | 100 | 100 | 100 |
| M2 | 100 | 100 | 100 | 100 | 100 |
| M3 | 200 | 200 | 100 | 200 | 200 |
| M4 | 200 | 200 | 100 | 100 | 150 |
| M5 | 25 | 0 | 25 | 25 | 0 |
| M6 | 100 | 100 | 100 | 50 | 50 |
| M7 | 75 | 50 | 50 | 25 | 50 |
| M8 | 25 | 25 | 25 | 12.5 | 25 |
| M_BIOMASS | 50 | 0 | 50 | 50 | 50 |
| ; |  |  |  |  |  |

Explanation:

The output of dpaplot is represented as four parts of information about producibility plot. Here, the model used for this analysis is the same as the model in GNI analysis. The first part is for Metabolites to genes, which showing the mapping from metabolites to its essential gene sets. For each line, the first column is the name of a metabolite which having essential genes. The other columns are the set of gene names associated with this metabolite, where the names are tab-delimited. By this simple format, the mapping information can be easily read by dpasig problem, where the output of dpaplot will be used as input file for problem dpasig. The second part of Genes to metabolites shows the mapping from gene to its affected metabolites. The predicted results for each gene are delimited by a space line, and only the genes having knockout reactions are listed. Explanation of output format is as follows. Gene: name of gene; KO Reactions: knockout reactions associated with this gene; Metabolites: a set of metabolites associated with this gene. The fourth part present an matrix of producibility plot. The matrix is represented as metabolites in rows by genes in columns, where first column corresponds names of metabolites and first row corresponds wildtype and names of genes. The Matrix of producibility shows the maximal fluxes towards a metabolite in wild-type mode and the maximal flux in gene-knockout models.

Output for dpasig

| Metabolite signals from microarray data: Array1 Array2 | | | | | |
| --- | --- | --- | --- | --- | --- |
|  | |  |  |  |  |
| Signals for up-regulated genes: | | | |  |  |
|  |  | |  |  |  |
|  | Array1 | | Array2 |  |  |
| M3 | 1.8 | | 2.2 |  |  |
| M4 | 1.55 | | 2 |  |  |
| M5 | 2.5 | | 1.6 |  |  |
| M6 | 1.3 | | 1.8 |  |  |
| M7 | 1.8 | | 2 |  |  |
| M8 | 1.3 | | 2 |  |  |
| M_BIOMASS | 1.9 | | 0 |  |  |
| /// |  | |  |  |  |
|  |  | |  |  |  |
| Signals for down-regulated genes: | | | | |  |
|  |  | |  |  |  |
|  | Array1 | | Array2 |  |  |
| M3 | 0 | | 0 |  |  |
| M4 | -1.8 | | -1.5 |  |  |
| M5 | -1.6 | | -1.8 |  |  |
| M6 | -1.8 | | -1.5 |  |  |
| M7 | -1.8 | | -1.65 |  |  |
| M8 | -2 | | -1.5 |  |  |
| M_BIOMASS | 0 | | -1.65 |  |  |
| ; |  | |  |  |  |

Explanation:

Above results give the metabolite signals which are calculated as median of expression ratios of essential genes for each metabolite. In terms of whether expression ratios being positive or negative, two signals for each metabolite can be obtained which separately shown as two matrices in output, they are Signals for up-regulated genes and Signals for down-regulated genes. The matrix is represented as metabolites in rows by microarray in columns, where first column corresponds names of metabolites and first row corresponds names of microarrays, here zero means no gene can be found for this metabolite signal.

5. Add signalling constrains on model

Cellular signalling is important part in controlling the metabolic network. In MUFINS, you are able to add signalling constrains to control reaction fluxes. Signalling constrains were implemented in pure linear formulation based on paper ‘A Linearized Constraint-Based Approach for Modeling Signaling Networks’.

Steps of adding signalling constrains are explained as follows:

1) Define the signalling species (activators/inhibitors). You can let an existing metabolite becoming activator/inhibitor by adding a transfer reaction. You can also use external activator/inhibitor by adding a transport reaction. You can also add an inhibitor directly using an existing metabolite.

In example model, transfer reaction S1 defines an activator aM1 which is provided by M1 with upper bound flux of 45; using M5 as inhibitor for R4 so that flux of R4 is bounded by production flux of M5. Note that here the production of metabolite is only for non-reversible reactions which can produce this metabolite.

2) Add signalling species into target reactions. You can add activators/inhibitors on the right-hand of target reaction equation as following formats:

+ & Activator

+ ~ Intensity Inhibitor

where symbol ‘&’ and ‘~’ indicates activator and inhibitor respectively; For inhibitor you can set inhibition intensity, so the sum of the flux of target reaction and the multiplication of intensity and inhibitor production flux should be less than the maximum flux of target reaction. One reaction can have multiple activators/inhibitors and one activator/inhibitor can affect multiple reactions. Note that here inhibited target reactions are assumed in forward direction.

In example model, reaction R4 is regulated by activator aM1 and inhibitor iM3 with intensity of 10.

Example model

| R4 | M6 + & aM1 + ~ 3 M5 = M7 | 0 | 100 | g6 | #Reaction 4 |
| --- | --- | --- | --- | --- | --- |
| S1 | M1 = aM1 | 0 | 20 |  | #M1 -> activator aM1 |
| R1 | M1 + 2 M2 = M5 + M6 | 0 | 100 | g1 | #Reaction 1 |
| R2 | M3 = 2 M1 + M7 | -100 | 100 | g4 | #Reaction 2 |
| R3 | M4 = M1 + M7 | -100 | 100 | g4 | #Reaction 3 |
| R5 | 2 M6 = M5 + M8 | -100 | 100 | g3 | #Reaction 5 |
| R6 | M7 = M5 + M8 | -100 | 100 | g2 | #Reaction 6 |
| BIOMASS | 2 M5 + M8 = M_BIOMASS | 0 | 1000 |  | #Biomass |
| GROWTH | M_BIOMASS = M_BIOMASS_xt | 0 | 1000 |  | #Growth |
| NR1 | M1_xt = M1 | 0 | 100 |  | #Nutrient M1 |
| NR2 | M2_xt = M2 | 0 | 100 |  | #Nutrient M2 |
| NR3 | M3_xt = M3 | 0 | 100 |  | #Nutrient M3 |
| NR4 | M4_xt = M4 | 0 | 100 |  | #Nutrient M4 |

Run FBA by maximizing the R4 flux with command:

./sfba -i model_sig.sfba -X _xt -p fba -o R4 -c

We will get following results:

#20: OPTIMAL (R4)

BIOMASS 20 #Biomass

GROWTH 20 #Growth

NR1 40 #Nutrient M1

NR2 40 #Nutrient M2

NR3 0 #Nutrient M3

NR4 0 #Nutrient M4

R1 20 #Reaction 1

R2 -0 #Reaction 2

R3 -0 #Reaction 3

R4 20 #Reaction 4

R5 0 #Reaction 5

R6 20 #Reaction 6

S1 20 #M1 -> activator aM1

aM1__col 20 #

Explanation:

Without signalling constraints, reaction R4 can get maximum flux of 50. However, R4 can be inhibited by M5 by the amount of flux of inhibitor’s production flux multiplied by its intensity that is 20 × 3 = 60, so R4 can only be activated by amount of flux of 100 – 60 = 40. Reaction R4 can only be activated by activator aM1 by the amount of flux of 20 as upper bound flux of aM1 = 20, so overall R4 can get maximum flux of 20.

# References

1. Orth, J.D., I. Thiele, and B.O. Palsson, *What is flux balance analysis?* Nature Biotechnology, 2010. **28**(3): p. 245-248.

2. Feist, A.M., et al., *A genome-scale metabolic reconstruction for Escherichia coli K-12 MG1655 that accounts for 1260 ORFs and thermodynamic information.* Molecular Systems Biology, 2007. **3**.

3. Bordbar, A., et al., *Model-driven multi-omic data analysis elucidates metabolic immunomodulators of macrophage activation.* Molecular Systems Biology, 2012. **8**.

4. Fisher, C.P., et al., *QSSPN: Dynamic Simulation of Molecular Interaction Networks Describing Gene Regulation, Signalling and Whole-Cell Metabolism in Human Cells.* Bioinformatics, 2013. **29**(24): p. 3181-3190.

5. Puchalka, J. and A.M. Kierzek, *Bridging the gap between stochastic and deterministic regimes in the kinetic simulations of the biochemical reaction networks.* Biophysical Journal, 2004. **86**(3): p. 1357-1372.

6. Rohr, C., W. Marwan, and M. Heiner, *Snoopy-a unifying Petri net framework to investigate biomolecular networks.* Bioinformatics, 2010. **26**(7): p. 974-975.

7. Kolodkin, A., et al., *Optimization of stress response through the nuclear receptor-mediated cortisol signalling network.* Nature Communications, 2013. **4**: p. 1972.

8. Thiele, I., et al., *A community-driven global reconstruction of human metabolism.* Nature Biotechnology, 2013. **31**(5): p. 419.

9. Gille, C., et al., *HepatoNet1: a comprehensive metabolic reconstruction of the human hepatocyte for the analysis of liver physiology.* Molecular Systems Biology, 2010. **6**: p. 411.

10. Jain, M., et al., *Metabolite Profiling Identifies a Key Role for Glycine in Rapid Cancer Cell Proliferation.* Science, 2012. **336**(6084): p. 1040-1044.
